# Combined Methylphenidate and Selective Serotonin Reuptake Inhibitors in Adults With Attention-Deficit/Hyperactivity Disorder

**DOI:** 10.1001/jamanetworkopen.2024.38398

**Published:** 2024-10-09

**Authors:** Dong Yun Lee, Chungsoo Kim, Yunmi Shin, Rae Woong Park

**Affiliations:** 1 Department of Biomedical Informatics, Ajou University School of Medicine, Suwon, South Korea; 2 Department of Medical Sciences, Graduate School of Ajou University, Suwon, South Korea; 3 Section of Cardiovascular Medicine, Department of Internal Medicine, Yale University School of Medicine, New Haven, Connecticut; 4 Department of Psychiatry, Ajou University School of Medicine, Suwon, South Korea; 5 Department of Biomedical Sciences, Ajou University Graduate School of Medicine, Suwon, South Korea

## Abstract

**Importance:**

Depression is a common comorbidity of adult attention-deficit/hyperactivity disorder (ADHD), and the combination of methylphenidate and selective serotonin reuptake inhibitors (SSRIs) is a frequently prescribed treatment. However, there is limited clinical evidence on the safety of this medication combination in adults with ADHD.

**Objective:**

To evaluate the safety of administering a combination of SSRI and methylphenidate in adults with ADHD and comorbid depression.

**Design, Setting, and Participants:**

This cohort study obtained data from a nationwide claims database in South Korea from January 2016 to February 2021. Participants were adults aged 18 years or older with a diagnosis of ADHD and depressive disorder who were prescribed methylphenidate. Comparisons of 4 groups who received prescriptions were conducted: (1) SSRI plus methylphenidate (hereafter, SSRI) group vs methylphenidate-only group and (2) methylphenidate plus fluoxetine (hereafter, fluoxetine) group vs methylphenidate plus escitalopram (hereafter, escitalopram) group (compared to find a preferable treatment option). Data analysis was conducted between July and December 2023.

**Exposures:**

New users of the methylphenidate and SSRI combination among adults with both ADHD and depressive disorder.

**Main Outcomes and Measures:**

A total of 17 primary and secondary outcomes, including neuropsychiatric and other events, were assessed, with respiratory tract infection used as a control outcome. Groups were matched at a 1:1 ratio using a propensity score to balance confounders. A Cox proportional hazards regression model was used to calculate hazard ratio (HRs) and 95% CIs. Subgroup analysis by sex and sensitivity analyses in varying epidemiologic settings were conducted.

**Results:**

The study included 17 234 adults with ADHD (mean [SD] age at study entry, 29.4 [10.8] years; 9079 females [52.7%]). There was no difference in the risk of outcomes between the methylphenidate-only and SSRI groups, except for a lower risk of headache in the SSRI group (HR, 0.50; 95% CI, 0.24-0.99). In sensitivity analyses of fluoxetine vs escitalopram, the risk of hypertension (HR: 1:n matching, 0.26; 95% CI, 0.08-0.67) and hyperlipidemia (HR: 1:n matching, 0.23; 95% CI, 0.04-0.81) was lower in the fluoxetine group than in the escitalopram group.

**Conclusions and Relevance:**

Results of this study revealed no significant increase in adverse event risk associated with use of SSRI plus methylphenidate vs methylphenidate alone in adults with ADHD and comorbid depression. Instead, the combination was associated with a lower risk of headache.

## Key Points

**Question:**

Is the combination of methylphenidate and selective serotonin reuptake inhibitors (SSRIs) safe in adults with attention-deficit/hyperactivity disorder (ADHD)?

**Findings:**

In this cohort study of 17 234 adults with ADHD, use of SSRIs combined with methylphenidate demonstrated a safe profile and was associated with a reduced risk of headaches.

**Meaning:**

The findings of this study suggest that combining SSRI and methylphenidate is not associated with increased risk of adverse events in adults with ADHD and comorbid depression.

## Teaser

This cohort study assesses 17 outcomes associated with use of combined methylphenidate with selective serotonin reuptake inhibitors (SSRIs) in adults with attention-deficit/hyperactivity disorder (ADHD) and depressive disorder.

## Introduction

Adult attention-deficit/hyperactivity disorder (ADHD) affects 2.5% of adults worldwide and is associated with severe functional and psychosocial impairments accompanied by substantial personal and societal costs.^1,2,3^ Symptoms of ADHD usually begin in childhood,^4^ but in adulthood the clinical presentation of ADHD encompasses a broader range of emotional dysregulation and functional impairments.^5^ Both stimulant and nonstimulant medications are advised for the pharmacological management of ADHD, with substantially greater use among adults in numerous countries.^6,7,8^ According to previous studies, depression often coexists with adult ADHD, having a prevalence of coexistence between 18.6% and 53.3%.^9,10^ Adults with both ADHD and depression experience more severe illness trajectories than adults with ADHD alone.^11^ A selective serotonin reuptake inhibitor (SSRI) may be added to the stimulant medication for adults with ADHD and comorbid depression.^12^ Although there is limited interaction information, stimulants are known to increase the plasma concentration of SSRIs, which may have adverse effects.^13,14^

There is a lack of large-scale safety research on the combined use of methylphenidate and SSRIs despite the high rate of comorbid depression in adult ADHD. A small-scale randomized clinical trial evaluating the safety of the combined methylphenidate and SSRI reported adverse effects, such as headache, weight loss, palpitations, and tremors.^15^ Animal studies have reported that augmenting an SSRI with methylphenidate may affect methylphenidate addiction or increase the susceptibility to cocaine use.^16,17^ Considering that depressive symptoms substantially mediate the relationship between ADHD and quality of life,^18^ evaluating the safety of combination therapy could be crucial to developing a plan of treatment.

Therefore, we aimed to evaluate the safety of administering a combination of SSRI and methylphenidate in adults with ADHD and comorbid depression, extending the previous ASSURE study (Adolescent ADHD and SSRI Use in Real-World Data).^19^ We also conducted a head-to-head assessment of the safety profiles of different SSRIs, specifically fluoxetine and escitalopram.

## Methods

### Study Design and Data Source

This retrospective observational cohort study used the Health Insurance Review and Assessment Service (HIRA), a nationwide claims database in South Korea, from January 2016 to February 2021 (eMethods in Supplement 1). The Ajou University Medical Center Institutional Review Board approved the study and waived the informed consent requirement because deidentified data were used. Analyses of deidentified data were performed in accordance with local laws and regulations. We followed the Strengthening the Reporting of Observational Studies in Epidemiology (STROBE) reporting guideline.

### Study Population and Exposure

All detailed code lists are available in eAppendix 2 in Supplement 1. For the study population, we identified adults (aged ≥18 years) with ADHD and depressive disorder diagnoses who were prescribed methylphenidate. To ensure minimal validity for initial diagnosis and baseline covariates, we excluded patients who had been enrolled in the HIRA database for less than 1 year before the first date of the methylphenidate prescription (eFigure 1 in Supplement 1). Patients who were prescribed nonstimulant ADHD medication (atomoxetine, bupropion, or clonidine) were also excluded. The population was divided into 4 study groups: the methylphenidate-only group, the SSRI plus methylphenidate group (hereafter, SSRI group), the methylphenidate plus fluoxetine group (hereafter, fluoxetine group), and the methylphenidate plus escitalopram group (hereafter, escitalopram group). The SSRI group included approved ingredients in South Korea: fluoxetine, escitalopram, sertraline, and paroxetine. We performed analyses for the sertraline and paroxetine groups, as described in the study protocol (eAppendix 1 in Supplement 1), but excluded these analyses because they did not meet the confounder adjustment criteria due to the small sample size.

The methylphenidate-only group was composed of patients who were prescribed methylphenidate for the first time and were antidepressant naive according to their medical history. The SSRI group was composed of patients who were prescribed both methylphenidate and SSRI for the first time and excluded those prescribed antidepressants other than SSRIs. The fluoxetine and escitalopram groups included patients who were prescribed fluoxetine or escitalopram only, respectively, without other SSRIs in the SSRI group. The schematic visualization for the study groups is presented in eFigure 1 in Supplement 1. In all groups, the date of the first prescription for methylphenidate served as the index date. However, for the SSRI, fluoxetine, and escitalopram groups, the index date was restricted to cases where the date of the first prescription for methylphenidate was the same as the date of the first prescription for the SSRIs.

### Outcomes and Follow-Up

All 17 outcomes were defined according to diagnostic codes in the SNOMED-CT (Systematized Nomenclature of Medicine–Clinical Terms) classification (eAppendix 2 in Supplement 1). Primary neuropsychiatric outcomes included anxiety disorder, tic disorder, mania, sleep disorder, and ADHD-related hospitalization. The ADHD-related hospitalization was defined as any hospitalization with both ADHD diagnosis and individual psychotherapy but without hospitalization in the previous 2 weeks. Secondary safety outcomes included tremor, headache, seizure, dizziness, arrhythmia, hypertension, anemia, hyperlipidemia, traumatic injury, and gastrointestinal events (abdominal pain, constipation, and nausea or vomiting). All study outcomes were limited to new-onset events except traumatic injury, gastrointestinal events, and ADHD-related hospitalization. We also validated the results through analysis, using respiratory tract infection as a control outcome.

Patients were followed up from the next day of the index date to either the last date of observed treatment (main analysis using the as-treated approach), last observation in the HIRA database, end point occurrence, or censoring event, whichever occurred first. Each treatment was considered as continued if the patient received a new prescription within 30 days of the last date of the previous prescription. Treatment discontinuation was defined as the time of the last prescription after which no additional prescriptions were given within 30 days, and the discontinued date was defined as the 30 days after the last administration. Censoring events were defined as events in which patients were exposed to comparator treatment or other antidepressants (eFigure 1 in Supplement 1). Censoring that occurred in 1 group was independent of the censoring of matched patients in the other group.

### Statistical Analysis

All categorical variables were denoted as frequency and percentage. Continuous variables are presented as mean and SD or median and IQR. Baseline characteristics were ascertained within a 12-month preindex period. A propensity score (PS) was calculated for adjusting the confounding bias between 2 study groups,^20^ and for estimating empirical equipoise. We deemed 2 groups as comparable when over 50% of the patients in each comparative pair had propensity scores ranging from 0.25 to 0.75.^21^ We used Lasso logistic regression to estimate the PS by age group (in 5-year increments), sex, year of the index date, Charlson Comorbidity Index (range: 0-33, with higher scores indicating higher mortality rate), and all coded information of the diagnosis and drug. Diagnosis and drug use were dichotomized, and if a patient had no code, they were considered to have no disease or no prescription. We developed 2 separate PS models for each comparison: (1) methylphenidate-only vs SSRI and (2) fluoxetine vs escitalopram. Study groups were matched 1:1 based on the PS. We deemed a variable as balanced if its absolute standardized mean difference (ASMD) was less than 0.1. Outcome incidence rates (IRs) per 1000 person-years were estimated. A Cox proportional hazards regression model was used to calculate hazard ratios (HRs) with 95% CIs. Only the treatment exposure was included as a covariate in the Cox proportional hazards regression model. Kaplan-Meier curve and log-rank test were used to derive the cumulative incidence and compare between-group differences. Two-sided *P* < .05 indicated statistical significance. A subgroup analysis by sex was conducted.

Sensitivity analyses were performed in the following analysis settings: PS adjustment, follow-up strategy, and study population. We modified the PS adjustment methods as 1 to maximum (1:n) matching or stratification with 5 strata. We also changed the follow-up strategy to the intention-to-treat (ITT) approach to estimate the outcome of being observed under a given treatment regardless of nonadherence.^18^ In the ITT strategy, we included only patients who were observed for 1 year and were followed up to the study end date or the outcome occurrence. Additionally, we changed the definition of the study population. First, we allowed a 30-day gap between the methylphenidate and SSRI. The index date was based on the first prescription of SSRI, with the methylphenidate prescription falling within 30 days before the first SSRI prescription. Second, we expanded the methylphenidate-only group to include patients who received any ADHD medication, such as atomoxetine, bupropion, and clonidine. Third, we expanded the SSRI group to include patients who received any antidepressants (antidepressants group). Details of the antidepressant group are provided in eAppendixes 1 and 2 in Supplement 1.

All analyses were performed using R, version 4.1.0, and its open-source statistical packages (R Project for Statistical Computing).^22^ Data analysis was conducted between July and December 2023.

## Results

### Cohort Characteristics

This analysis included 17 234 patients with ADHD, of whom 9873 (57.3%) were assigned to the SSRI group and 7361 (42.7%) to the methylphenidate-only group (eFigure 2 in Supplement 1). The mean (SD) age at study entry was 29.4 (10.8) years, with 8155 individuals (47.3%) identifying as male and 9079 (52.7%) as female. The median (IQR) follow-up time was 87 (48-259) days for the SSRI group and 55 (36-172) days for the methylphenidate-only group. After matching, the median (IQR) follow-up time was 88 (49-273) days for the SSRI group and 51 (35-167) days for the methylphenidate-only group. The SSRI group was divided into the escitalopram group (5150 [52.2%]) and the fluoxetine group (2791 [28.3%]). All study group pairs were comparable based on the empirical equipoise (eFigure 3 in Supplement 1).

Baseline characteristics of the overall study population before and after PS matching are presented in Table 1 and eTable 1 in Supplement 1. After PS matching, all baseline characteristics were balanced between 5181 matched pairs for the SSRI group and the methylphenidate-only group (all ASMD <0.10) (Table 1; eFigures 4 and 5 in Supplement 1). The proportions of males were 48.9% in the SSRI group and 49.2% in the methylphenidate-only group, whereas the proportions of females were 51.1% and 50.8%, respectively. In both groups, most patients ranged in age from 18 to 39 years (86.1% and 85.8%, respectively). The mean (SD) doses of initial methylphenidate prescription were 17.8 (10.3) mg in the SSRI group and 17.7 (12.2) mg in the methylphenidate-only group (ASMD = 0.01).

**Table 1.** Comparisons of Baseline Characteristics, Comorbidities, and Concomitant Drugs in Adults With ADHD and Comorbid Depression After Propensity Score Matching

Characteristic	Group, No. (%)		ASMD	Group, No. (%)		ASMD
	SSRI (n = 5181)	MPH-only (n = 5181)		Fluoxetine (n = 2577)	Escitalopram (n = 2577)	
Sociodemographic						
Sex						
Male	2533 (48.9)	2549 (49.2)	0	1023 (39.7)	1023 (39.7)	0
Female	2648 (51.1)	2632 (50.8)	0	1554 (60.3)	1554 (60.3)	0
Age, y						
18-39	4460 (86.1)	4445 (85.8)	0	2250 (87.3)	2247 (87.2)	0.01
40-64	637 (12.3)	653 (12.6)	0	320 (12.4)	307 (11.9)	0.06
≥65	84 (1.6)	83 (1.6)	0	7 (0.3)	23 (0.9)	0.08
Ethnicity: Korean	5181 (100.0)	5181 (100.0)	0	2577 (100.0)	2577 (100.0)	0
Index year						
2017	596 (11.5)	580 (11.2)	0	332 (12.9)	353 (13.7)	0.02
2018	1015 (19.6)	969 (18.7)	0.02	530 (20.6)	528 (20.5)	0
2019	1503 (29.0)	1518 (29.3)	0	760 (29.5)	771 (29.9)	0.01
2020	2067 (39.9)	2114 (40.8)	0.02	955 (37.1)	925 (35.9)	0.03
Psychiatric comorbidity						
SUD	114 (2.2)	110 (2.2)	0	72 (2.8)	62 (2.4)	0.03
Conduct disorder	52 (1.0)	49 (1.0)	0	23 (0.9)	26 (1.0)	0.02
Personality disorder	88 (1.7)	93 (1.8)	0	70 (2.7)	62 (2.4)	0.02
ASD	26 (0.5)	28 (0.5)	0	23 (0.9)	21 (0.8)	0
Intellectual disability	52 (1.0)	57 (1.1)	0.01	21 (0.8)	23 (0.9)	0.01
Medication use						
Anticholinergics	41 (0.8)	47 (0.9)	0.01	18 (0.7)	15 (0.6)	0.01
Antiepileptics	497 (9.6)	503 (9.7)	0	260 (10.1)	247 (9.6)	0.02
Antipsychotics	855 (16.5)	876 (16.9)	0.01	526 (20.4)	500 (19.4)	0.02
Anxiolytics	1192 (23.0)	1243 (24.0)	0.02	959 (37.2)	894 (34.7)	0.05
Initial prescribed dose of MPH, mean (SD), mg	17.8 (10.3)	17.7 (12.2)	0.01	17.6 (11.4)	16.3 (9.8)	0.09

Abbreviations: ADHD, attention-deficit/hyperactivity disorder; ASD, autism spectrum disorder; ASMD, absolute standardized mean difference; MPH, methylphenidate; SSRI, selective serotonin reuptake inhibitor; SUD, substance use disorder.

In the comparison between SSRI types (fluoxetine vs escitalopram), all baseline characteristics were balanced (all ASMDs <0.1; Table 1). The mean (SD) dose of initial methylphenidate prescription was 17.6 (11.4) mg in the fluoxetine group and 16.3 (9.8) mg in the escitalopram group (ASMD = 0.09).

### Outcome Assessment

**Figure 1.**
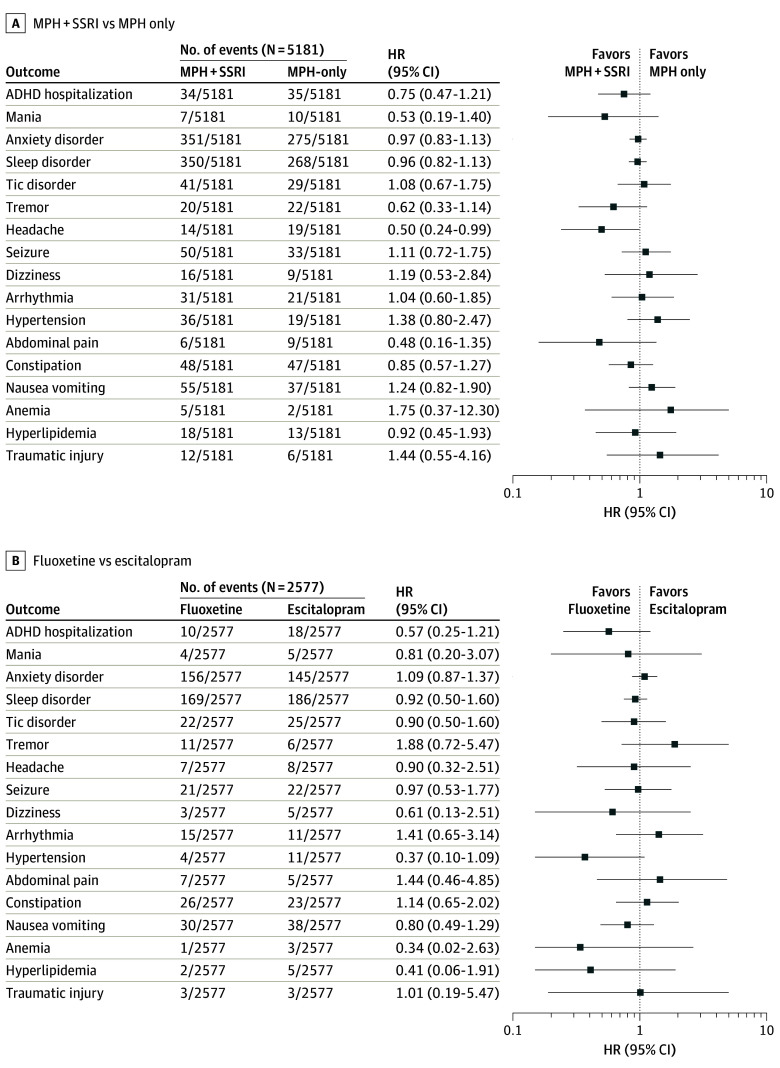
Forest Plots of Outcome Estimates of Selective Serotonin Reuptake Inhibitor (SSRI) Plus Methylphenidate (MPH) vs MPH-Only Groups and Fluoxetine vs Escitalopram Groups Neuropsychiatric outcomes include attention-deficit/hyperactivity disorder (ADHD) hospitalization, mania, anxiety disorder, sleep disorder, and tic disorder. Error bars represent 95% CIs.

Outcome risks, except for the headache risk, did not significantly differ between the SSRI and methylphenidate-only groups (Table 2; Figure 1). Specifically, the SSRI group had lower risk of headache than the methylphenidate-only group (14 vs 19 cases; HR, 0.50 [95% CI, 0.24-0.99]). However, some outcomes that were not statistically significant (eg, anemia and traumatic injury) had wide CIs, indicating uncertainty in the results. The number of events, person-years, and IRs are presented in eTable 2 in Supplement 1. The control outcome did not show statistically significant differences in any setting, including the sensitivity analyses.

**Table 2.** Risk of Outcome Events for the SSRI Plus Methylphenidate vs Methylphenidate-Only Groups and Fluoxetine vs Escitalopram Groups

Outcome	Incidence rate by group^a^		HR (95% CI)	Incidence rate by group^a^		HR (95% CI)
	SSRI (n = 5181)	MPH-only (n = 5181)		Fluoxetine (n = 2577)	Escitalopram (n = 2577)	
Primary						
Mania	3.11	7.02	0.53 (0.19-1.40)	4.14	5.04	0.81 (0.20-3.07)
Anxiety disorder	256.05	298.09	0.97 (0.83-1.13)	275.19	252.33	1.09 (0.87-1.37)
Sleep disorder	206.89	244.25	0.96 (0.82-1.13)	240.13	256.66	0.92 (0.50-1.60)
Tic disorder	18.52	20.63	1.08 (0.67-1.75)	23.32	25.88	0.90 (0.50-1.60)
ADHD hospitalization^b^	15.08	24.69	0.75 (0.47-1.21)	10.33	18.26	0.57 (0.25-1.21)
Secondary						
Tremor	8.95	15.52	0.62 (0.33-1.14)	11.51	6.06	1.88 (0.72-5.47)
Headache	6.24	13.41	0.50 (0.24-0.99)^c^	7.29	8.12	0.90 (0.32-2.51)
Seizure	22.90	23.74	1.11 (0.72-1.75)	22.13	22.75	0.97 (0.53-1.77)
Dizziness	7.13	6.34	1.19 (0.53-2.84)	3.13	5.09	0.61 (0.13-2.51)
Arrhythmia	13.97	14.90	1.04 (0.60-1.85)	15.67	11.16	1.41 (0.65-3.14)
Hypertension	16.16	13.41	1.38 (0.80-2.47)	4.16	11.23	0.37 (0.10-1.09)
Abdominal pain	2.65	6.31	0.48 (0.16-1.35)	7.24	5.04	1.44 (0.46-4.85)
Constipation	21.37	33.15	0.85 (0.57-1.27)	27.01	23.40	1.14 (0.65-2.02)
Nausea or vomiting	24.51	26.00	1.24 (0.82-1.90)	31.37	38.81	0.80 (0.49-1.29)
Anemia	2.21	1.40	1.75 (0.37-12.30)	1.03	3.03	0.34 (0.02-2.63)
Hyperlipidemia	8.01	9.15	0.92 (0.45-1.93)	2.07	5.06	0.41 (0.06-1.91)
Traumatic injury	5.31	4.20	1.44 (0.55-4.16)	3.10	3.02	1.01 (0.19-5.47)
Control outcome^d^	2.21	1.40	1.61 (0.34-11.27)	2.06	3.03	0.75 (0.10-4.53)

Abbreviations: ADHD, attention-deficit/hyperactivity disorder; HR, hazard ratio; MPH, methylphenidate; SSRI, selective serotonin reuptake inhibitor.

^a^ Incidence rates were calculated as case per 1000 person-years.

^b^ Hospitalization indicates a hospitalization with the presence of an ADHD diagnosis.

^c^ Statistically significant.

^d^ Control outcome indicates respiratory tract infection.

In the comparison between SSRI types, none of the outcome risks were statistically significantly different. In contrast to previous findings in adolescents,^19^ we found no difference in tic disorder between the fluoxetine and escitalopram groups (22 vs 25 cases; HR, 0.90 [95% CI, 0.50-1.60]) (Table 2; eTable 3 in Supplement 1).

In the subgroup analyses, there was a significant difference in tremor between the male subgroups in the SSRI and methylphenidate-only groups (HR, 0.23; 95% CI, 0.05-0.77); however, none of the comparisons between the male subgroups in the fluoxetine and escitalopram group were significantly different (eTables 4 and 5 in Supplement 1). For the female subgroups, there were significant differences in headache (HR, 0.35; 95% CI, 0.14-0.83) and hypertension (HR, 3.24; 95% CI, 1.31-9.73) between the SSRI and methylphenidate-only groups. Unlike the male subgroups, the female subgroups showed a significant difference in hypertension (HR, 0.31; 95% CI, 0.09-0.88) between the fluoxetine and escitalopram groups (eTable 5 in Supplement 1).

### Sensitivity Analyses

**Figure 2.**
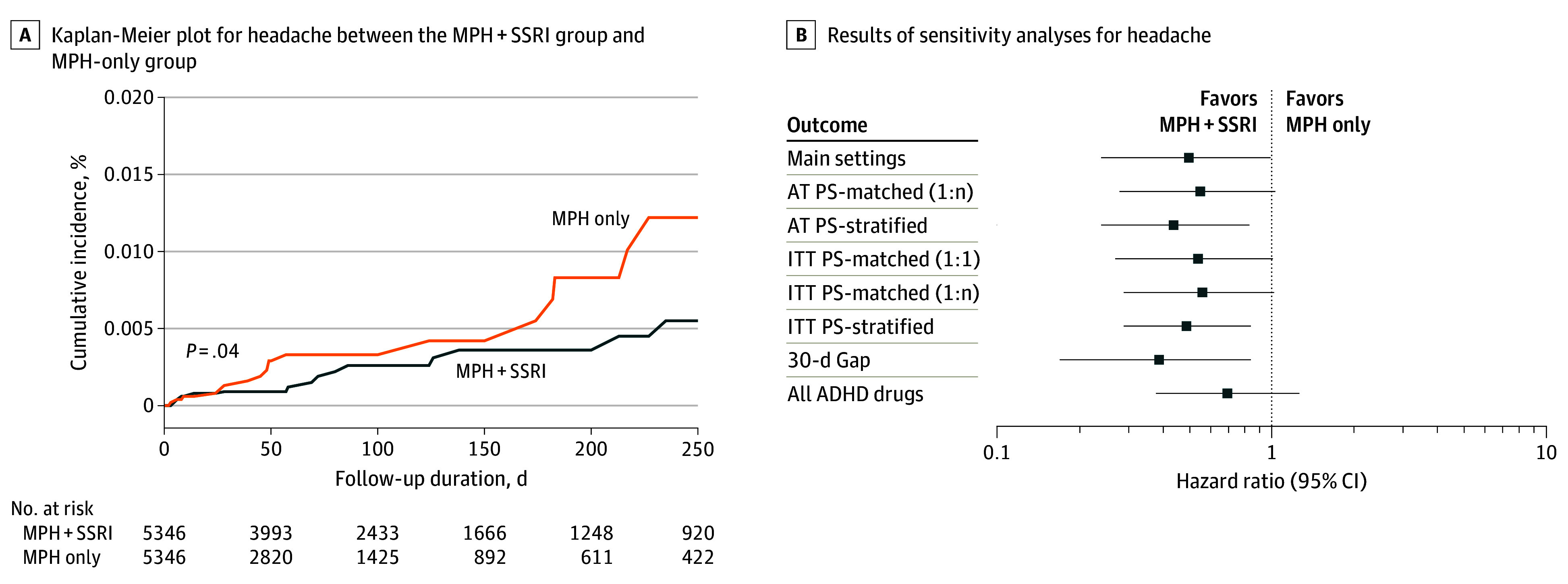
Comparison of Headache Between the Selective Serotonin Reuptake Inhibitor (SSRI) Plus Methylphenidate (MPH) Group and MPH-Only Group AT indicates as treated; PS, propensity score; ITT, intention to treat; and 1:n, 1 to maximum matching. Error bars represent 95% CIs.

The baseline characteristics of the overall study population before and after PS matching according to sensitivity analysis are presented in eTables 6, 7, and 10 in Supplement 1). The overall sensitivity analysis results are shown in eTables 8, 9, and 11 in Supplement 1). The risk of headache was consistently lower in the SSRI group than in the methylphenidate-only group (stratification: HR, 0.45 [95% CI, 0.24-0.83]; ITT with stratification: HR, 0.49 [95% CI, 0.29-0.84]) (eTable 8 in Supplement 1). Figure 2 shows a Kaplan-Meier curve of the main and sensitivity analyses for headache. For the other outcomes, no consistent results were confirmed by various sensitivity analysis settings.

Between the fluoxetine and escitalopram groups, there was no difference in the main setting, but there were significant differences in hypertension and hyperlipidemia between the groups consistently across several settings. Specifically, the risk of hypertension (HR: 1:n matching, 0.26 [95% CI, 0.08-0.67]; stratification, 0.38 [95% CI, 0.15-0.82]; ITT with 1:1 matching, 0.39 [95% CI, 0.15-0.89]; ITT with 1:n matching, 0.37 [95% CI, 0.15-0.78]; ITT with stratification, 0.41 [95% CI, 0.17-0.88]) and the risk of hyperlipidemia (HR: 1:n matching, 0.23 [95% CI, 0.04-0.81]; stratification, 0.22 [95% CI, 0.03-0.82]; ITT with 1:1 matching, 0.18 [95% CI, 0.03-0.68]; ITT with 1:n matching, 0.15 [95% CI, 0.02-0.50]; ITT with stratification, 0.32 [95% CI, 0.09-0.88]) were lower in the fluoxetine group than in the escitalopram group (eTable 9 in Supplement 1). The antidepressant group showed a lower risk of tremor (IR, 7.52 vs 15.09; HR, 0.51 [95% CI, 0.28-0.92]) and headache (IR, 6.17 vs 13.85; HR, 0.51 [95% CI, 0.27-0.95]) than the methylphenidate-only group (eTable 11 in Supplement 1).

## Discussion

In this comprehensive, nationwide retrospective cohort study, we examined the risk of neuropsychiatric and other safety outcomes based on the presence or types of SSRI prescribed for adults with ADHD and depressive disorder. We found that combined SSRIs and methylphenidate demonstrated a safe profile in the study group and was associated with a reduced risk of headaches. In the comparison of fluoxetine with escitalopram, most of the differences in safety were not significant. However, the risks of hyperlipidemia and hypertension were significantly higher in the escitalopram group than the fluoxetine group, with consistency in some analytic settings.

In this study, the risks of all outcomes were not significantly greater in the SSRI group than in the methylphenidate-only group. The findings align with those of the previous ASSURE study,^19^ in which SSRIs plus methylphenidate had generally safe profiles in adolescents with ADHD and depression. Given that there are more reports of antidepressant adverse effects in children than in adults,^23^ the finding that the concomitant use of SSRI and methylphenidate is safe in adults (similar to a study in adolescents^19^) seems reasonable. Patkar et al^24^ also showed that the combination of methylphenidate and therapeutic doses of antidepressants was safe and tolerable. However, of the outcomes that were not statistically significant, anemia and traumatic injury had wide CIs, indicating uncertainty in the results. There are possible explanations for this finding. For example, it is possible that anemia is associated with SSRIs given that SSRIs can induce platelet dysfunction and thus may increase bleeding risk.^25^ Although ADHD medication has been reported to reduce the risk of traumatic injury, it has also been reported to increase the risk when used with SSRIs.^26^ These results need to be interpreted with caution and warrant future research with larger datasets. In this study, the risk of headaches was lower when SSRIs were added to methylphenidate. The association between ADHD and headache may be mediated by internalization symptoms and sleep disturbances.^27,28,29^ Considering that antidepressants can play a role in effective reduction of emotional symptoms and sleep problems,^30,31^ improvements in the mediated symptoms may have been a factor in the difference in headache risk between the SSRI and methylphenidate-only groups. Additionally, fluoxetine and paroxetine have been reported to be moderately effective for migraine.^32,33,34^ Enhancing serotonin levels may be advantageous for headache prevention under the migraine pathophysiological process.^35,36^ Although headache is frequently noted as an adverse effect of antidepressants, a meta-analysis found that it is seldom linked to the use of antidepressants.^37^ In addition to headache, the risk of tremor decreased when SSRIs were combined with methylphenidate in several settings of this study. Patients with functional tremor may benefit from antidepressants, especially if they have current (or a history of) depression or anxiety.^38^ Both the SSRI and methylphenidate-only groups in this study comprised patients diagnosed with depression, suggesting that antidepressants may have had a role in decreasing tremor. Further studies that account for the dose response are needed.^39^

The subgroup analysis by sex showed no difference in most safety outcomes for the combination of SSRI and methylphenidate except for the risks of headache, tremor, and hypertension. In both sexes, the risks of tremor and headache tended to be low in the SSRI group. However, the risk of tremor was significant in males only, and the risk of headache was significant in females only. Although the prevalence of tremor was independent of sex, the duration was shorter in females than in males.^40^ Migraines were twice as prevalent in females, and females with ADHD were more sensitive to pain than males.^41,42^ These factors might explain the differences between sexes; however, since tremor and headache showed similar patterns, the reduced number of patients might have had a greater impact. Compared with males, females using SSRIs and methylphenidates together were found to have an increased risk of hypertension. Previous studies have reported no substantial changes in blood pressure when SSRIs and methylphenidate were combined.^15,43^ However, sex differences in hypertension do exist; in particular, females exhibited higher rates of awareness, treatment, and control compared with males.^44^ Another hypothesis is that SSRIs may be associated with metabolic risk,^45^ which is closely related to hypertension.^46^ Considering that females with depression have a higher metabolic risk than males,^47^ the sex difference in metabolic risk may have played a role in the increased risk of hypertension with SSRI use in females. In particular, eating disorders associated with weight gain occur more frequently in females than in males, and sex steroid hormones have been identified as a biological mechanism in this context.^48^ Given the paucity of studies examining sex differences in the association of the SSRI plus methylphenidate combination with blood pressure,^49^ the findings in this study warrant further confirmation.

The risk of hyperlipidemia was lower in the fluoxetine group than in the escitalopram group in the sensitivity analysis. In a prospective study, fluoxetine use was associated with reductions in weight-related biomarkers, including triglycerides. Compared with fluoxetine, escitalopram was associated with increased serum triglyceride levels after 16 weeks of use.^46^ A meta-analysis also found that fluoxetine was associated with reduced triglycerides in patients with diabetes.^50^ However, fluoxetine has 5-HT_2C_ receptor antagonist properties that affect feeding, glucose homeostasis, and the energy efficiency of physical activity, which may explain the difference in hyperlipidemia risk between the 2 groups.^51^ Furthermore, the risk of hypertension was lower in the fluoxetine group than in the escitalopram group. Use of SSRIs has a known minimal association with autonomic system activity and reduced blood pressure^52^; therefore, another explanation to consider is the association between hyperlipidemia and hypertension. It has been reported that elevated total cholesterol and non–high-density lipoprotein cholesterol are associated with an increased risk of hypertension.^53^ Considering that fluoxetine may be associated with decreased risk of hyperlipidemia, it is possible that a similar mechanism could also be associated with the reduced risk of hypertension for fluoxetine than for escitalopram. Nevertheless, these findings necessitate further investigation.

### Limitations

This study has several limitations that should be considered when interpreting the findings. First, there were challenges in availability of some data. For example, we could not consider the socioeconomic status and severity of diseases in the confounder adjustment due to unavailability. Specifically, the clinical assessments for the type and severity of ADHD or depressive disorders could not be conducted because it is difficult to directly identify symptoms of ADHD or depression from claims data. The difference in the follow-up period between the methylphenidate-only group and the SSRI group may be associated with the difference in clinical severity. Although we tried to adjust a large number of variables, there may be potential bias due to the difference in baseline severity. Second, there was uncertainty in some of the findings due to the small sample size and the outcome occurrence. Specifically, some of the nonsignificant results showed wide 95% CI ranges. The reason we reported these data is that the number of patients and the outcomes were already ascertained and unmodifiable in the observational study, and the results were from a representative database covering all of South Korea. The data will be important for meta-analysis and for generating research hypothesis in future studies. Third, in terms of generalizability, the study did not cover cases wherein SSRIs were administered first and methylphenidate was added and did not make direct comparisons of sertraline with paroxetine. We did not plan to examine such cases, which require further study. Comparisons of sertraline and paroxetine were planned in the protocol but could not be included due to insufficient confounding adjustment. Fourth, the follow-up period was short, and there was a difference between the SSRI and methylphenidate groups in the main as-treated approach. Differences in the follow-up period in the 2 groups may bias the estimates; however, it was difficult to confirm this bias by comparing it with the results of the ITT approach due to the numerous nonsignificant results. In a future study, we may consider matched censoring approaches.

## Conclusions

In this cohort study of adults with ADHD, there was no significant increase in risks of adverse events associated with use of the combination of SSRI plus methylphenidate vs methylphenidate alone in adults with ADHD and comorbid depression. The risk of headache was also lower with the concomitant use of SSRI and methylphenidate than use of methylphenidate alone. No significant difference in most adverse events was found between fluoxetine and escitalopram, but fluoxetine may be associated with improved outcomes for hypertension and hyperlipidemia.

## Notes

Supplement 1. **eAppendix 1.** The ASSURE-Extend Study Protocol **eAppendix 2.** The List of Code for Study Variables Used in Definition **eMethods.** Study Design and Database **eFigure 1.** Schematic Visualization for the Cohort Definition and As-Treated Follow-up Strategy **eFigure 2.** Flow Diagram Between the SSRI Group and the MPH-Only Group **eTable 1.** Baseline Characteristics, Comorbidities, and Concomitant Drugs in Adult ADHD Patients With Depression Before the Propensity Score Matching **eFigure 3.** Empirical Equipoise Between the Comparison Groups **eFigure 4.** Scatter Plots Between Before and After the Propensity Score Adjustment Between the SSRI and MPH-Only Groups **eFigure 5.** Scatter Plots Between Before and After the Propensity Score Adjustment Between the Fluoxetine and Escitalopram Groups **eTable 2.** Incidence of Outcome Events Between the SSRI and MPH-Only Groups **eTable 3.** Incidence of Outcome Events Between the Fluoxetine and Escitalopram Groups **eTable 4.** Risk of Outcome Events Between the SSRI and MPH Alone Groups in the Subgroup Analysis by Sex **eTable 5.** Risk of Outcome Events Between the Fluoxetine and Escitalopram Groups in the Subgroup Analysis by Sex **eTable 6.** Comparisons of Baseline Characteristics, Comorbidities, and Concomitant Drugs Between the SSRI and MPH-Only Groups After Propensity Score Matching in Sensitivity Analyses **eTable 7.** Comparisons of Baseline Characteristics, Comorbidities, and Concomitant Drugs Between the Fluoxetine and Escitalopram Groups After Propensity Score Matching in Sensitivity Analyses **eTable 8.** Results of Sensitivity Analyses Between the SSRI and MPH-Only Groups **eTable 9.** Results of Sensitivity Analyses Between the Fluoxetine and Escitalopram Groups **eTable 10.** Comparisons of Baseline Characteristics, Comorbidities, and Concomitant Drugs Between the Antidepressants and MPH-Only Groups After Propensity Score Matching in Sensitivity Analyses **eTable 11.** Results of Sensitivity Analyses Between the Antidepressants and MPH-Only Groups
eReferences

Supplement 2.
Data Sharing Statement
