# Supplementary material for: Combined Methylphenidate and Selective Serotonin Reuptake Inhibitors in Adults With Attention-Deficit/Hyperactivity Disorder
Source: JAMA Netw Open. 2024 Oct 9;7(10):e2438398. doi: 10.1001/jamanetworkopen.2024.38398 (PMC11581539; doi:10.1001/jamanetworkopen.2024.38398)
Supplement: Supplement 1. — eAppendix 1. The ASSURE-Extend Study Protocol eAppendix 2. The List of Code for Study Variables Used in Definition eMethods. Study Design and Database eFigure 1. Schematic Visualization for the Cohort Definition and As-Treated Follow-up Strategy eFigure 2. Flow Diagram Between the SSRI Group and the MPH-Only Group eTable 1. Baseline Characteristics, Comorbidities, and Concomitant Drugs in Adult ADHD Patients With Depression Before the Propensity Score Matching eFigure 3. Empirical Equipoise Between the Comparison Groups eFigure 4. Scatter Plots Between Before and After the Propensity Score Adjustment Between the SSRI and MPH-Only Groups eFigure 5. Scatter Plots Between Before and After the Propensity Score Adjustment Between the Fluoxetine and Escitalopram Groups eTable 2. Incidence of Outcome Events Between the SSRI and MPH-Only Groups eTable 3. Incidence of Outcome Events Between the Fluoxetine and Escitalopram Groups eTable 4. Risk of Outcome Events Between the SSRI and MPH Alone Groups in the Subgroup Analysis by Sex eTable 5. Risk of Outcome Events Between the Fluoxetine and Escitalopram Groups in the Subgroup Analysis by Sex eTable 6. Comparisons of Baseline Characteristics, Comorbidities, and Concomitant Drugs Between the SSRI and MPH-Only Groups After Propensity Score Matching in Sensitivity Analyses eTable 7. Comparisons of Baseline Characteristics, Comorbidities, and Concomitant Drugs Between the Fluoxetine and Escitalopram Groups After Propensity Score Matching in Sensitivity Analyses eTable 8. Results of Sensitivity Analyses Between the SSRI and MPH-Only Groups eTable 9. Results of Sensitivity Analyses Between the Fluoxetine and Escitalopram Groups eTable 10. Comparisons of Baseline Characteristics, Comorbidities, and Concomitant Drugs Between the Antidepressants and MPH-Only Groups After Propensity Score Matching in Sensitivity Analyses eTable 11. Results of Sensitivity Analyses Between the Antidepressants and MPH-Only Groups eReferences [file jamanetwopen-e2438398-s001.pdf]

Supplemental Online Content

Lee DY, Kim C, Shin Y, Park RW. Combined methylphenidate and selective serotonin reuptake inhibitors in adults with attention-deficit/hyperactivity disorder. *JAMA Netw Open*. 2024;7(10):e2438398. doi:10.1001/jamanetworkopen.2024.38398

eAppendix 1. The ASSURE-Extend Study Protocol
eAppendix 2. The List of Code for Study Variables Used in Definition
eMethods. Study Design and Database
eFigure 1. Schematic Visualization for the Cohort Definition and As-Treated Follow-up Strategy
eFigure 2. Flow Diagram Between the SSRI Group and the MPH-Only Group
eTable 1. Baseline Characteristics, Comorbidities, and Concomitant Drugs in Adult ADHD Patients With Depression Before the Propensity Score Matching
eFigure 3. Empirical Equipoise Between the Comparison Groups
eFigure 4. Scatter Plots Between Before and After the Propensity Score Adjustment Between the SSRI and MPH-Only Groups
eFigure 5. Scatter Plots Between Before and After the Propensity Score Adjustment Between the Fluoxetine and Escitalopram Groups
eTable 2. Incidence of Outcome Events Between the SSRI and MPH-Only Groups
eTable 3. Incidence of Outcome Events Between the Fluoxetine and Escitalopram Group
eTable 4. Risk of Outcome Events Between the SSRI and MPH Alone Groups in the Subgroup Analysis by Sex
eTable 5. Risk of Outcome Events Between the Fluoxetine and Escitalopram Groups in the Subgroup Analysis by Sex
eTable 6. Comparisons of Baseline Characteristics, Comorbidities, and Concomitant Drugs Between the SSRI and MPH-Only Groups After Propensity
eTable 7. Comparisons of Baseline Characteristics, Comorbidities, and Concomitant Drugs Between the Fluoxetine and Escitalopram Groups After Propensity Score Matching in Sensitivity Analyses
eTable 8. Results of Sensitivity Analyses Between the SSRI and MPH-Only Groups
eTable 9. Results of Sensitivity Analyses Between the Fluoxetine and Escitalopram Groups
eTable 10. Comparisons of Baseline Characteristics, Comorbidities, and Concomitant Drugs Between the Antidepressants and MPH-Only Groups After Propensity Score Matching in Sensitivity Analyses
eTable 11. Results of Sensitivity Analyses Between the Antidepressants and MPH-Only Groups
eReferences

This supplemental material has been provided by the authors to give readers additional information about their work.

eAppendix 1. The ASSURE-Extend Study Protocol

© 2024 Lee DY et al. *JAMA Network Open*

AJOU UNIVERSITY

Comparative Effectiveness and Safety of Selective Serotonin Reuptake Inhibitors in Adult Attention-Deficit/Hyperactivity Disorder and comorbid depression : The ASSURE-Extend Study Protocol

Version: 1.0

Date: March 2, 2023

Authors:

Dong Yun Lee, MD*, Department of Biomedical Informatics, Ajou University School of Medicine, Suwon, South Korea

Chungsoo Kim, PharmD*, Department of Biomedical Sciences, Ajou University Graduate School of Medicine, Suwon, South Korea

Yunmi Shin, MD[t], Department of Psychiatry, Ajou University School of Medicine

Rae Woong Park, MD, PhD[t], Department of Biomedical Informatics, Ajou University Graduate School of Medicine, Suwon, South Korea

* Contributed equally as a co-first author

[t] Contributed equally as a co-corresponding author

Corresponding author:

Yunmi Shin, MD, Professor

Department of Psychiatry, Ajou University School of Medicine, Suwon, South Korea

ymshin@ajou.ac.kr

Rae Woong Park, MD, PhD, Professor

Department of Biomedical Informatics, Ajou University School of Medicine, Suwon, South Korea

Comparative Effectiveness Research Protocol

© 2024 Lee DY et al. *JAMA Network Open*

veritas@ajou.ac.kr

Financial supports: This work is supported by the Health Insurance Review & Assessment Service (HIRA}. The views expressed are those of the author(s} and not necessarily those of the HIRA.

© 2024 Lee DY et al. *JAMA Network Open*

1 List of abbreviations

ADHD	Attention-Deficit/Hyperactivity Disorder
MPH	Methylphenidate
SSRI	Selective Serotonin Reuptake Inhibitor

2 Abstract

Attention-deficit/hyperactivity disorder (ADHD} is one of the most common neurobehavioral disorders[1]. Recently, more and more cases of ADHD persisting into adulthood or new-onset ADHD at adulthood suggest that a new approach is needed to manage ADHD. Unlike children, adults can have many deficits in higher-level executive functioning and emotional control and have many comorbid diseases due to diverse environmental exposures.[2, 3] Establishing treatment strategies according to comorbidities in ADHD patients is important, but the related evidence is weak. Most of Adult with ADHD also have many comorbidities such as anxiety disorder, depressive disorder, substance abuse, and autism spectrum disorder.[4-8]

Especially, ADHD is closely related to depressive disorder. There are previous studies on high comorbidity rate, biological linkage or causality and its clinical outcomes.[9-13] When establishing a treatment strategy for ADHD patients with depression, the clinical hurdles for the use of antidepressants are concerns about changes in the patients' condition (i.e., suicidality[14-17], etc.} and an increase in adverse effects.[16]

Although the first-line treatment for ADHD and depressive disorder is recommended in different guidelines,[18, 19] the evidence for effectiveness and safety evaluation of concomitant use of those drugs is sparse. Therefore, in this study, we aimed to evaluate the real-world evidence for comparative effectiveness and safety of the co-use of selective serotonin reuptake inhibitors (SSRIs}, the fist recommended drug for depression, in ADHD patients (Adolescent ADHD and SSRI Use in Real-world Data - Extend to Adult: ASSURE Extend study}. We also aimed to evaluate the outcome systemically through comparison between user vs non-user, between SSRI ingredient level as head-to-head study.

3 Amendments and Updates

0.1	16 February 2023	C Kim	Initial draft
0.9	25 February 2023	C Kim	Finalize draft
1.0	2 March 2023	C Kim	Release version 1

© 2024 Lee DY et al. *JAMA Network Open*

4 Rationale and Background

The most used drug for the treatment of ADHD is psychostimulant, which includes MPH, dextroamphetamine, and lisdexamfetamine, for about 90% of the total ADHD prescription.[20, 21] MPH effectively ameliorate the symptoms of ADHD and MPH has the best safety/coverage ratio than other ADHD drugs although adverse events including affective symptoms and weight loss.[16, 22]

The prevalence of depression is 16-26% in ADHD,[23] and these patients takes ADHD medications and antidepressants together according to the clinical guidelines.[18, 19] It has been reported that antidepressant resistance occurs a lot in patients with ADHD, therefore a higher intensity treatment should be prescribed.[24, 25] However, there are some concerns for co-medication with antidepressant for ADHD, which are due to adverse events of such as suicidal behaviors.[14-17] In addition, the possibility of increased adverse events due to the drug interactions between ADHD medications and antidepressants have been reported in previous studies. For example, as most of the available antidepressants results in an increase in the synaptic availability of serotonin or norepinephrine, MPH also increases monoamines postsynaptically, as well as increasing additive or synergistic effects and finally increasing adverse reactions like serotonin syndrome.[26-28] Also specific antidepressants induce gene regulation related in MPH, there are concerns about coadministration of them.[29, 30]

In general, guidelines for antidepressants applied to patients with ADHD are applied according to age, but there are few studies in which effectiveness and safety was evaluated according to the presence and type of antidepressants in ADHD patients considering the interaction with MPH. Hence, we aimed to conduct comparative effectiveness research to establish real-world evidence for the safety of MPH and SSRIs in patients with ADHD.

5 Study Objectives

5.1 Objectives

The overall goal of this study is conducting comparative effectiveness research to establish evidence for effectiveness and safety of concomitant antidepressant in adult patients with attention-deficit/hyperactivity disorder.

The primary objective is comparing the risk of safety outcomes which include neuropsychiatric events, cardiovascular events, and other events during concomitant use of methylphenidate and SSRIs among adult patients with ADHD.

The secondary objective is comparing the risk of effectiveness outcomes which include psychiatric hospitalisation during concomitant use of methylphenidate and SSRIs among adult patients with ADHD.

© 2024 Lee DY et al. *JAMA Network Open*

5.2 Primary Hypothesis

There are no differences in the risk of safety outcomes among subjects with or without SSRIs used to treat comorbid depression in adults with ADHD.

There are no differences in the risk of safety outcomes among subjects between SSRI ingredients (Escitalopram, fluoxetine, sertraline, and paroxetine} used to treat comorbid depression in adults with ADHD.

5.3 Secondary Hypothesis

There are no differences in the risk of effectiveness outcomes among subjects with or without SSRIs used to treat comorbid depression in adults with ADHD.

There are no differences in the risk of effectiveness outcomes among subjects between SSRI ingredients (Escitalopram, fluoxetine, sertraline, and paroxetine} used to treat comorbid depression in adults with ADHD.

6 Research methods

6.1 Study Design

6.1.1 Overview

This study will be a retrospective, observational cohort study. By 'retrospective' we mean the study will use data already collected at the start of the study. By 'observational' we mean no intervention will take place in the course of this study. By 'cohort study' we mean two cohorts, a treatment and comparator cohort, will be followed from index date (start of first exposure} to specific end date, and assessed for the occurrence of the outcomes of interest.

For primary analysis, the treatment cohort will be users of any SSRIs with MPH. The comparator cohort will be no users of SSRIs (MPH alone user}. For both groups we restrict to people with first ADHD and depression diagnoses. For secondary analysis, the treatment cohort will be user of specific ingredient of SSRIs with MPH and the comparator cohort will be another ingredient with MPH (e.g., fluoxetine vs escitalopram, etc.}.

The baseline characteristics will be investigated. For minimizing confounding bias between study cohorts, propensity score adjustment will be conducted. The primary outcome of is neuropsychiatric events. The Cox proportional hazard models will be used to assess the hazard ratios between the two exposure cohorts.

© 2024 Lee DY et al. *JAMA Network Open*

6.2 Study population

6.2.1 Primary study population

The primary study population is designed for a comparative analysis of users of concomitant SSRI and MPH, and users of MPH alone. This population will include all subjects in the database who meet the following criteria: (Note: the index date refers to the day of the first prescription of the SSRI for the SSRI group, or the day of the first MPH prescription for the MPH alone group}.

- Adolescents who prescribed MPH for ADHD and have depressive disorder
  - □18 years old adults
  - ADHD diagnosis for the first time in the patient's history on or before the index date
  - Depressive disorder diagnosis for the first time in the patient's history on or before the index date
  - At least 365 days of observation time prior to the index date
  - No other ADHD medications such as atomoxetine, clonidine, or bupropion.

6.2.2 Secondary study population

The secondary study population is intended for a comparative analysis of SSRI ingredients. This population will include all subjects in the database who meet the following criteria (Note: the index date refers to the days of the first prescription of the SSRIs}

- Adults who prescribed MPH for ADHD and prescribed any SSRI for depressive disorder.
  - □18 years old adults
  - ADHD diagnosis for the first time in the patient's history on or before the index date
  - Depressive disorder diagnosis for the first time in the patient's history on or before the index date
  - At least 365 days of observation time prior to the index date
  - No other ADHD medications such as atomoxetine, clonidine, and bupropion
  - No other antidepressant drugs except the target ingredient before the index date

6.2.3 Study population for sensitivity analyses

In South Korea, there are other treatment options for ADHD treatment such as atomoxetine, clonidine, besides MPH. A sensitivity analysis including the corresponding options will be conducted. The study population for the sensitivity analysis will be included who meet the following criteria: (note: the index date is the day of the first prescription of SSRI}

- Adults who prescribed ADHD medications and have depressive disorder
  - □18 years old adults
  - ADHD diagnosis for the first time in the patient's history on or before the index date

© 2024 Lee DY et al. *JAMA Network Open*

- Depressive disorder diagnosis for the first time in the patient's history on or before the index date
- At least 365 days of observation time prior to the index date

6.3 Exposures

6.3.1 Comparison summary

Type	Treatment (target)	Comparator (reference)
**Main analysis**	SSRI + MPH	MPH alone
	Fluoxetine + MPH	Escitalopram + MPH
	Sertraline + MPH	Escitalopram + MPH
	Paroxetine + MPH	Escitalopram + MPH
	Sertraline + MPH	Fluoxetine + MPH
	Paroxetine + MPH	Fluoxetine + MPH
	Sertraline + MPH	Paroxetine + MPH
**Sensitivity analysis 1.** Expand MPH to any of ADHD medications	SSRI + ADHD medication	ADHD medication alone
	Fluoxetine + ADHD medication	Escitalopram + ADHD medication
	Sertraline + ADHD medication	Escitalopram + ADHD medication
	Paroxetine + ADHD medication	Escitalopram + ADHD medication
	Sertraline + ADHD medication	Fluoxetine + ADHD medication
	Paroxetine + ADHD medication	Fluoxetine + ADHD medication
	Sertraline + ADHD medication	Paroxetine + ADHD medication
**Sensitivity analysis 2.** Allllow 30 days gap between the two drugs	SSRI + MPH (within gap)	MPH alone
	Fluoxetine + MPH (within gap)	Escitalopram + MPH (within gap)
	Sertraline + MPH (within gap)	Escitalopram + MPH (within gap)
	Paroxetine + MPH (within gap)	Escitalopram + MPH (within gap))
	Sertraline + MPH (within gap)	Fluoxetine + MPH (within gap)
	Paroxetine + MPH (within gap)	Fluoxetine + MPH (within gap)
	Sertraline + MPH (within gap)	Paroxetine + MPH (within gap)
	SSRI + ADHD med (within gap)	ADHD medication alone
	Fluoxetine + ADHD med (within gap)	Escitalopram + ADHD med (within gap)
	Sertraline + ADHD med (within gap)	Escitalopram + ADHD med (within gap)
	Paroxetine + ADHD med (within gap)	Escitalopram + ADHD med (within gap)
	Sertraline + ADHD med (within gap)	Fluoxetine + ADHD med (within gap)
	Paroxetine + ADHD med (within gap)	Fluoxetine + ADHD med (within gap)
	Sertraline + ADHD med (within gap)	Paroxetine + ADHD med (within gap)
**Sensitivity analysis 3.** Expand SSRI to antidepressants	Antidepressant + MPH	MPH alone
	Antidepressant + ADHD med	ADHD medication alone
	Antidepressant + MPH (within gap)	MPH alone
	Antidepressant + ADHD med (within gap)	ADHD medication alone

6.3.2 Treatment 1: new SSRI user with MPH

- **Cohort Entry Events**

  People with continuous observation of 365 days before event may enter the cohort when observing any of the following:

© 2024 Lee DY et al. *JAMA Network Open*

- Any SSRI prescription for the first time in the person's history (index date}
- with age greater or equal to 18
- having at least 1 prescription of MPH at the date of index date

Limit cohort entry events to the earliest event per person.

- **Inclusion Criteria**

1. Patients with ADHD
   - Entry events having at least 1 diagnosis of ADHD for the first time in the person's history, starting anytime on or before the index date
2. Patients with depression
   - Entry events having at least 1 diagnosis of depression for the first time in the person's history, starting anytime on or before the index date.
3. Patients without antidepressants prior to the index date
   - Entry events having no prescription of the antidepressant, starting any time prior to the 1 day before the index date.
4. Patients without other ADHD medications
   - Entry events having no prescription of other ADHD medications.

- **Cohort Exit**

The cohort end date will be based on a continuous exposure to SSRI and MPH allowing 30 days between prescriptions, adding 30 days after exposure ends using the days supply and exposure end date for exposure duration.

The patient exits the cohort when encountering any of the following events:

- Other antidepressants except SSRIs

6.3.3 Treatment 2: new fluoxetine user with MPH

- **Cohort Entry Events**

People with continuous observation of 365 days before event may enter the cohort.

when observing any of the following:

- Any fluoxetine prescription for the first time in the person's history (index date}
- with age greater or equal to 18
- having at least 1 prescription of MPH at the date of index date

Limit cohort entry events to the earliest event per person.

- **Inclusion Criteria**

1. Patients with ADHD
   - Entry events having at least 1 diagnosis of ADHD for the first time in the person's history, starting anytime on or before the index date
2. Patients with depression
   - Entry events having at least 1 diagnosis of depression for the first time in the person's history, starting anytime on or before the index date.
3. Patients without antidepressants prior to the index date
   - Entry events having no prescription of the antidepressant, starting any time prior to 1 day before the index date.

© 2024 Lee DY et al. *JAMA Network Open*

4. Patients without other ADHD medications
   - Entry events having no prescription of other ADHD medications.

- **Cohort Exit**

  The cohort end date will be based on a continuous exposure to fluoxetine and MPH allowing 30 days between prescriptions, adding 30 days after exposure ends using the days supply and exposure end date for exposure duration.

  The patient exits the cohort when encountering any of the following events:
  - Other antidepressant without fluoxetine

6.3.4 Treatment 3: new sertraline user with MPH

- **Cohort Entry Events**

  People with continuous observation of 365 days before event may enter the cohort

  when observing any of the following:
  - Any sertraline prescription for the first time in the person's history (index date}
  - with age greater or equal to 18
  - having at least 1 prescription of MPH at the date of index date

  Limit cohort entry events to the earliest event per person.

- **Inclusion Criteria**

1. Patients with ADHD
   - Entry events having at least 1 diagnosis of ADHD for the first time in the person's history, starting anytime on or before the index date
2. Patients with depression
   - Entry events having at least 1 diagnosis of depression for the first time in the person's history, starting anytime on or before the index date.
3. Patients without antidepressants prior to the index date
   - Entry events having no prescription of the antidepressant, starting any time prior to 1 day before the index date.
4. Patients without other ADHD medications
   - Entry events having no prescription of other ADHD medications.

- **Cohort Exit**

  The cohort end date will be based on a continuous exposure to sertraline and MPH allowing 30 days between prescriptions, adding 30 days after exposure ends using the days supply and exposure end date for exposure duration

  The patient exits the cohort when encountering any of the following events:
  - Other antidepressant without sertraline

6.3.5 Treatment 4: new paroxetine user with MPH

- **Cohort Entry Events**

  People with continuous observation of 365 days before event may enter the cohort.

  when observing any of the following:

© 2024 Lee DY et al. *JAMA Network Open*

- Any paroxetine prescription for the first time in the person's history (index date}
- with age greater or equal to 18
- having at least 1 prescription of MPH at the date of index date

Limit cohort entry events to the earliest event per person.

- **Inclusion Criteria**

1. Patients with ADHD
   - Entry events having at least 1 diagnosis of ADHD for the first time in the person's history, starting anytime on or before the index date.
2. Patients with depression
   - Entry events having at least 1 diagnosis of depression for the first time in the person's history, starting anytime on or before the index date.
3. Patients without antidepressants prior to the index date
   - Entry events having no prescription of the antidepressant, starting any time prior to 1 day before the index date.
4. Patients without other ADHD medications
   - Entry events having no prescription of other ADHD medications.

- **Cohort Exit**

The cohort end date will be based on a continuous exposure to paroxetine and MPH allowing 30 days between prescriptions, adding 30 days after exposure ends using the days supply and exposure end date for exposure duration.

The patient exits the cohort when encountering any of the following events:

- Other antidepressant without paroxetine

6.3.6 Sensitivity treatment 1: new SSRI user with ADHD medication

- **Cohort Entry Events**

People with continuous observation of 365 days before event may enter the cohort when observing any of the following:

- Any SSRI prescription for the first time in the person's history (index date}
- with age greater or equal to 18
- having at least 1 prescription of ADHD medication at the date of index date

Limit cohort entry events to the earliest event per person.

- **Inclusion Criteria**

1. Patients with ADHD
   - Entry events having at least 1 diagnosis of ADHD for the first time in the person's history, starting anytime on or before the index date
2. Patients with depression
   - Entry events having at least 1 diagnosis of depression for the first time in the person's history, starting anytime on or before the index date.
3. Patients without antidepressants prior to the index date
   - Entry events having no prescription of the antidepressant, starting any time prior to 1 day before the index date.

© 2024 Lee DY et al. *JAMA Network Open*

- **Cohort Exit**

The cohort end date will be based on a continuous exposure to SSRI and ADHD medication allowing 30 days between prescriptions, adding 30 days after exposure ends using the days supply and exposure end date for exposure duration

The patient exits the cohort when encountering any of the following events:

- Other antidepressants except SSRIs

6.3.7 Sensitivity treatment 2: new fluoxetine user with ADHD medication

- **Cohort Entry Events**

People with continuous observation of 365 days before event may enter the cohort when observing any of the following:

- Any fluoxetine prescription for the first time in the person's history (index date}
- with age greater or equal to 18
- having at least 1 prescription of ADHD medication at the date of index date

Limit cohort entry events to the earliest event per person.

- **Inclusion Criteria**

1. Patients with ADHD
   - Entry events having at least 1 diagnosis of ADHD for the first time in the person's history, starting anytime on or before the index date
2. Patients with depression
   - Entry events having at least 1 diagnosis of depression for the first time in the person's history, starting anytime on or before the index date.
3. Patients without antidepressants prior to the index date
   - Entry events having no prescription of the antidepressant, starting any time prior to the index date.

- **Cohort Exit**

The cohort end date will be based on a continuous exposure to fluoxetine and ADHD medication allowing 30 days between prescriptions, adding 30 days after exposure ends using the days supply and exposure end date for exposure duration

The patient exits the cohort when encountering any of the following events:

- Other antidepressant except fluoxetine

6.3.8 Sensitivity treatment 2: new sertraline user with ADHD medication

- **Cohort Entry Events**

People with continuous observation of 365 days before event may enter the cohort when observing any of the following:

- Any sertraline prescription for the first time in the person's history (index date}
- with age greater or equal to 18
- having at least 1 prescription of ADHD medication at the date of index date

Limit cohort entry events to the earliest event per person.

© 2024 Lee DY et al. *JAMA Network Open*

- **Inclusion Criteria**
1. Patients with ADHD
   - Entry events having at least 1 diagnosis of ADHD for the first time in the person's history, starting anytime on or before the index date
2. Patients with depression
   - Entry events having at least 1 diagnosis of depression for the first time in the person's history, starting anytime on or before the index date.
3. Patients without antidepressants prior to the index date
   - Entry events having no prescription of the antidepressant, starting any time prior to the index date.

- **Cohort Exit**

The cohort end date will be based on a continuous exposure to sertraline and ADHD medication allowing 30 days between prescriptions, adding 30 days after exposure ends using the days supply and exposure end date for exposure duration

The patient exits the cohort when encountering any of the following events:

- Other antidepressants except sertraline

6.3.9 Sensitivity treatment 3: new paroxetine user with ADHD medication

- **Cohort Entry Events**

People with continuous observation of 365 days before event may enter the cohort when observing any of the following:

- Any paroxetine prescription for the first time in the person's history (index date}
- with age greater or equal to 18
- having at least 1 prescription of ADHD medication at the date of index date

Limit cohort entry events to the earliest event per person.

- **Inclusion Criteria**
1. Patients with ADHD
   - Entry events having at least 1 diagnosis of ADHD for the first time in the person's history, starting anytime on or before the index date
2. Patients with depression
   - Entry events having at least 1 diagnosis of depression for the first time in the person's history, starting anytime on or before the index date.
3. Patients without antidepressants prior to the index date
   - Entry events having no prescription of the antidepressant, starting any time prior to the index date.

- **Cohort Exit**

The cohort end date will be based on a continuous exposure to paroxetine and ADHD medication allowing 30 days between prescriptions, adding 30 days after exposure ends using the days supply and exposure end date for exposure duration

The patient exits the cohort when encountering any of the following events:

© 2024 Lee DY et al. *JAMA Network Open*

- Other antidepressants except paroxetine

6.3.10 Sensitivity treatment 5: new SSRI user with MPH (allowing 30 days gap between treatments)

- **Cohort Entry Events**

People with continuous observation of 365 days before event may enter the cohort when observing any of the following:

- Any SSRI prescription for the first time in the person's history (index date}
- with age greater or equal to 18
- having at least 1 prescription of MPH starting between 30 days before and 0 days after the index date

Limit cohort entry events to the earliest event per person.

- **Inclusion Criteria**

1. Patients with ADHD
   - Entry events having at least 1 diagnosis of ADHD for the first time in the person's history, starting anytime on or before the index date
2. Patients with depression
   - Entry events having at least 1 diagnosis of depression for the first time in the person's history, starting anytime on or before the index date.
3. Patients without antidepressants prior to the index date
   - Entry events having no prescription of the antidepressant, starting any time prior to the index date.
4. Patients without other ADHD medications
   - Entry events having no prescription of other ADHD medications

- **Cohort Exit**

The cohort end date will be based on a continuous exposure to SSRI and MPH allowing 30 days between prescriptions, adding 30 days after exposure ends using the days supply and exposure end date for exposure duration

The patient exits the cohort when encountering any of the following events:

- Other antidepressants except SSRIs

6.3.11 Sensitivity treatment 6: new fluoxetine user with MPH (allowing 30 days gap between two treatment)

- **Cohort Entry Events**

People with continuous observation of 365 days before event may enter the cohort when observing any of the following:

- Any fluoxetine prescription for the first time in the person's history (index date}
- with age greater or equal to 18
- having at least 1 prescription of MPH starting between 30 days before and 0 days after the index date

Limit cohort entry events to the earliest event per person.

© 2024 Lee DY et al. *JAMA Network Open*

- **Inclusion Criteria**

  1. Patients with ADHD
     - Entry events having at least 1 diagnosis of ADHD for the first time in the person's history, starting anytime on or before the index date
  2. Patients with depression
     - Entry events having at least 1 diagnosis of depression for the first time in the person's history, starting anytime on or before the index date.
  3. Patients without antidepressants prior to the index date
     - Entry events having no prescription of the antidepressant, starting any time prior to the index date.
  4. Patients without other ADHD medications
     - Entry events having no prescription of other ADHD medications

- **Cohort Exit**

  The cohort end date will be based on a continuous exposure to fluoxetine and MPH allowing 30 days between prescriptions, adding 30 days after exposure ends using the days supply and exposure end date for exposure duration

  The patient exits the cohort when encountering any of the following events:

  - Other antidepressants except fluoxetine

6.3.12 Sensitivity treatment 7: new sertraline user with MPH (allowing 30 days gap between two treatment)

- **Cohort Entry Events**

  People with continuous observation of 365 days before event may enter the cohort

  when observing any of the following:

  - Any sertraline prescription for the first time in the person's history (index date}
  - with age greater or equal to 18
  - having at least 1 prescription of MPH starting between 30 days before and 0 days after the index date

  Limit cohort entry events to the earliest event per person.

- **Inclusion Criteria**

  1. Patients with ADHD
     - Entry events having at least 1 diagnosis of ADHD for the first time in the person's history, starting anytime on or before the index date
  2. Patients with depression
     - Entry events having at least 1 diagnosis of depression for the first time in the person's history, starting anytime on or before the index date.
  3. Patients without antidepressants prior to the index date
     - Entry events having no prescription of the antidepressant, starting any time prior to the index date.
  4. Patients without other ADHD medications
     - Entry events having no prescription of other ADHD medications

© 2024 Lee DY et al. *JAMA Network Open*

- **Cohort Exit**

The cohort end date will be based on a continuous exposure to sertraline and MPH allowing 30 days between prescriptions, adding 30 days after exposure ends using the days supply and exposure end date for exposure duration

The patient exits the cohort when encountering any of the following events:

- Other antidepressants except sertraline

6.3.13 Sensitivity treatment 8: new paroxetine user with MPH (allowing 30 days gap between

- **Cohort Entry Events**

People with continuous observation of 365 days before event may enter the cohort when observing any of the following:

- Any paroxetine prescription for the first time in the person's history (index date}
- with age greater or equal to 18
- having at least 1 prescription of MPH starting between 30 days before and 0 days after the index date

Limit cohort entry events to the earliest event per person.

- **Inclusion Criteria**

1. Patients with ADHD
   - Entry events having at least 1 diagnosis of ADHD for the first time in the person's history, starting anytime on or before the index date
2. Patients with depression
   - Entry events having at least 1 diagnosis of depression for the first time in the person's history, starting anytime on or before the index date.
3. Patients without antidepressants prior to the index date
   - Entry events having no prescription of the antidepressant, starting any time prior to the index date.
4. Patients without other ADHD medications
   - Entry events having no prescription of other ADHD medications

- **Cohort Exit**

The cohort end date will be based on a continuous exposure to paroxetine and MPH allowing 30 days between prescriptions, adding 30 days after exposure ends using the days supply and exposure end date for exposure duration

The patient exits the cohort when encountering any of the following events:

- Other antidepressants except paroxetine

6.3.14 Sensitivity treatment 9: new SSRI user with ADHD medication (allowing 30 days gap between treatments)

- **Cohort Entry Events**

People with continuous observation of 365 days before event may enter the cohort

© 2024 Lee DY et al. *JAMA Network Open*

when observing any of the following:

- Any SSRI prescription for the first time in the person's history (index date}
- with age greater or equal to 18
- having at least 1 prescription of ADHD medication starting between 30 days before and 0 days after the index date

Limit cohort entry events to the earliest event per person.

- **Inclusion Criteria**

1. Patients with ADHD
   - Entry events having at least 1 diagnosis of ADHD for the first time in the person's history, starting anytime on or before the index date
2. Patients with depression
   - Entry events having at least 1 diagnosis of depression for the first time in the person's history, starting anytime on or before the index date.
3. Patients without antidepressants prior to the index date
   - Entry events having no prescription of the antidepressant, starting any time prior to the index date.

- **Cohort Exit**

The cohort end date will be based on a continuous exposure to SSRI and ADHD medications allowing 30 days between prescriptions, adding 30 days after exposure ends using the days supply and exposure end date for exposure duration

The patient exits the cohort when encountering any of the following events:

- Other antidepressants except SSRIs

6.3.15 Sensitivity treatment 10: new fluoxetine user with ADHD medication (allowing 30 days gap between treatments)

- **Cohort Entry Events**

People with continuous observation of 365 days before event may enter the cohort

when observing any of the following:

- Any fluoxetine prescription for the first time in the person's history (index date}
- with age greater or equal to 18
- having at least 1 prescription of ADHD medication starting between 30 days before and 0 days after the index date

Limit cohort entry events to the earliest event per person.

- **Inclusion Criteria**

1. Patients with ADHD
   - Entry events having at least 1 diagnosis of ADHD for the first time in the person's history, starting anytime on or before the index date
2. Patients with depression
   - Entry events having at least 1 diagnosis of depression for the first time in the person's history, starting anytime on or before the index date.
3. Patients without antidepressants prior to the index date

© 2024 Lee DY et al. *JAMA Network Open*

- Entry events having no prescription of the antidepressant, starting any time prior to the index date.

- **Cohort Exit**

The cohort end date will be based on a continuous exposure to fluoxetine and MPH allowing 30 days between prescriptions, adding 30 days after exposure ends using the days supply and exposure end date for exposure duration

The patient exits the cohort when encountering any of the following events:

- Other antidepressants except fluoxetine

6.3.16 Sensitivity treatment 11: new sertraline user with ADHD medication (allowing 30 days gap between treatments)

- **Cohort Entry Events**

People with continuous observation of 365 days before event may enter the cohort when observing any of the following:

- Any sertraline prescription for the first time in the person's history (index date}
- with age greater or equal to 18
- having at least 1 prescription of ADHD medication starting between 30 days before and 0 days after the index date

Limit cohort entry events to the earliest event per person.

- **Inclusion Criteria**

1. Patients with ADHD
   - Entry events having at least 1 diagnosis of ADHD for the first time in the person's history, starting anytime on or before the index date
2. Patients with depression
   - Entry events having at least 1 diagnosis of depression for the first time in the person's history, starting anytime on or before the index date.
3. Patients without antidepressants prior to the index date
   - Entry events having no prescription of the antidepressant, starting any time prior to the index date.

- **Cohort Exit**

The cohort end date will be based on a continuous exposure to sertraline and MPH allowing 30 days between prescriptions, adding 30 days after exposure ends using the days supply and exposure end date for exposure duration

The patient exits the cohort when encountering any of the following events:

- Other antidepressants except sertraline

6.3.17 Sensitivity treatment 12: new paroxetine user with ADHD medication (allowing 30 days gap between treatments)

- **Cohort Entry Events**

People with continuous observation of 365 days before event may enter the cohort

© 2024 Lee DY et al. *JAMA Network Open*

when observing any of the following:

- Any paroxetine prescription for the first time in the person's history (index date}
- with age greater or equal to 18
- having at least 1 prescription of ADHD medication starting between 30 days before and 0 days after the index date

Limit cohort entry events to the earliest event per person.

- **Inclusion Criteria**

1. Patients with ADHD
   - Entry events having at least 1 diagnosis of ADHD for the first time in the person's history, starting anytime on or before the index date
2. Patients with depression
   - Entry events having at least 1 diagnosis of depression for the first time in the person's history, starting anytime on or before the index date.
3. Patients without antidepressants prior to the index date
   - Entry events having no prescription of the antidepressant, starting any time prior to the index date.

- **Cohort Exit**

The cohort end date will be based on a continuous exposure to paroxetine and MPH allowing 30 days between prescriptions, adding 30 days after exposure ends using the days supply and exposure end date for exposure duration

The patient exits the cohort when encountering any of the following events:

- Other antidepressants except paroxetine

6.3.18 Sensitivity treatment 13: new antidepressant user with MPH

- **Cohort Entry Events**

People with continuous observation of 365 days before event may enter the cohort

when observing any of the following:

- Any antidepressant prescription for the first time in the person's history (index date}
- with age greater or equal to 18
- having at least 1 prescription of MPH at the date of index date

Limit cohort entry events to the earliest event per person.

- **Inclusion Criteria**

1. Patients with ADHD
   - Entry events having at least 1 diagnosis of ADHD for the first time in the person's history, starting anytime on or before the index date
2. Patients with depression
   - Entry events having at least 1 diagnosis of depression for the first time in the person's history, starting anytime on or before the index date.
3. Patients without antidepressants prior to the index date
   - Entry events having no prescription of the antidepressant, starting any time prior to the index date.

© 2024 Lee DY et al. *JAMA Network Open*

4. Patients without other ADHD medications
   - Entry events having no prescription of other ADHD medications

- **Cohort Exit**

The cohort end date will be based on a continuous exposure to antidepressant and MPH allowing 30 days between prescriptions, adding 30 days after exposure ends using the days supply and exposure end date for exposure duration

The patient exits the cohort when encountering any of the following events:

- No censoring in this cohort

6.3.19 Sensitivity treatment 14: new antidepressant user with ADHD medication

- **Cohort Entry Events**

People with continuous observation of 365 days before event may enter the cohort when observing any of the following:

- Any antidepressant prescription for the first time in the person's history (index date}
- with age greater or equal to 18
- having at least 1 prescription of ADHD medication at the date of index date

Limit cohort entry events to the earliest event per person.

- **Inclusion Criteria**

1. Patients with ADHD
   - Entry events having at least 1 diagnosis of ADHD for the first time in the person's history, starting anytime on or before the index date
2. Patients with depression
   - Entry events having at least 1 diagnosis of depression for the first time in the person's history, starting anytime on or before the index date.
3. Patients without antidepressants prior to the index date
   - Entry events having no prescription of the antidepressant, starting any time prior to the index date.

- **Cohort Exit**

The cohort end date will be based on a continuous exposure to antidepressant and ADHD medication allowing 30 days between prescriptions, adding 30 days after exposure ends using the days supply and exposure end date for exposure duration

The patient exits the cohort when encountering any of the following events:

- No censoring in this cohort

6.3.20 Sensitivity treatment 15: new antidepressant user with MPH (allowing 30 days gap between treatments)

- **Cohort Entry Events**

People with continuous observation of 365 days before event may enter the cohort when observing any of the following:

© 2024 Lee DY et al. *JAMA Network Open*

- Any antidepressant prescription for the first time in the person's history (index date}
- with age greater or equal to 18
- having at least 1 prescription of MPH starting between 30 days before and 0 days after the index date

Limit cohort entry events to the earliest event per person.

- **Inclusion Criteria**

1. Patients with ADHD
   - Entry events having at least 1 diagnosis of ADHD for the first time in the person's history, starting anytime on or before the index date
2. Patients with depression
   - Entry events having at least 1 diagnosis of depression for the first time in the person's history, starting anytime on or before the index date.
3. Patients without antidepressants prior to the index date
   - Entry events having no prescription of the antidepressant, starting any time prior to the index date.
4. Patients without other ADHD medications
   - Entry events having no prescription of other ADHD medications

- **Cohort Exit**

The cohort end date will be based on a continuous exposure to antidepressant and MPH allowing 30 days between prescriptions, adding 30 days after exposure ends using the days supply and exposure end date for exposure duration

The patient exits the cohort when encountering any of the following events:

- No censoring in this cohort

6.3.21 Sensitivity treatment 16: new antidepressant user with ADHD medication (allowing 30 days gap between treatments)

- **Cohort Entry Events**

People with continuous observation of 365 days before event may enter the cohort when observing any of the following:

- Any antidepressant prescription for the first time in the person's history (index date}
- with age greater or equal to 18
- having at least 1 prescription of ADHD medication starting between 30 days before and 0 days after the index date

Limit cohort entry events to the earliest event per person.

- **Inclusion Criteria**

1. Patients with ADHD
   - Entry events having at least 1 diagnosis of ADHD for the first time in the person's history, starting anytime on or before the index date
2. Patients with depression
   - Entry events having at least 1 diagnosis of depression for the first time in the person's history, starting anytime on or before the index date.

© 2024 Lee DY et al. *JAMA Network Open*

3. Patients without antidepressants prior to the index date
   - Entry events having no prescription of the antidepressant, starting any time prior to the index date.

- **Cohort Exit**

The cohort end date will be based on a continuous exposure to antidepressant and ADHD medication allowing 30 days between prescriptions, adding 30 days after exposure ends using the days supply and exposure end date for exposure duration

The patient exits the cohort when encountering any of the following events:

- No censoring in this cohort

6.3.22 Comparator 1: new MPH alone user

- **Cohort Entry Events**

People with continuous observation of 365 days before event may enter the cohort when observing any of the following:

- Any MPH prescription for the first time in the person's history (index date}
- with age greater or equal to 18

Limit cohort entry events to the earliest event per person.

- **Inclusion Criteria**

1. Patients with ADHD
   - Entry events having at least 1 diagnosis of ADHD for the first time in the person's history, starting anytime on or before the index date
2. Patients with depression
   - Entry events having at least 1 diagnosis of depression for the first time in the person's history, starting anytime on or before the index date.
3. Patients without antidepressants prior to the index date
   - Entry events having no prescription of the antidepressant, starting any time prior to the index date.
4. Patients without other ADHD medications
   - Entry events having no prescription of other ADHD medications

- **Cohort Exit**

The cohort end date will be based on a continuous exposure to MPH allowing 30 days between prescriptions, adding 30 days after exposure ends using the days supply and exposure end date for exposure duration.

The patient exits the cohort when encountering any of the following events:

- Any antidepressant exposure

6.3.23 Comparator 2: new escitalopram user with MPH

- **Cohort Entry Events**

People with continuous observation of 365 days before event may enter the cohort when observing any of the following:

© 2024 Lee DY et al. *JAMA Network Open*

- Any escitalopram prescription for the first time in the person's history (index date}
- with age greater or equal to 18
- having at least 1 prescription of MPH at the date of index date

Limit cohort entry events to the earliest event per person.

- **Inclusion Criteria**

1. Patients with ADHD
   - Entry events having at least 1 diagnosis of ADHD for the first time in the person's history, starting anytime on or before the index date
2. Patients with depression
   - Entry events having at least 1 diagnosis of depression for the first time in the person's history, starting anytime on or before the index date.
3. Patients without antidepressants prior to the index date
   - Entry events having no prescription of the antidepressant, starting any time prior to the index date.
4. Patients without other ADHD medications
   - Entry events having no prescription of other ADHD medications

- **Cohort Exit**

The cohort end date will be based on a continuous exposure to escitalopram and MPH allowing 30 days between prescriptions, adding 30 days after exposure ends using the days supply and exposure end date for exposure duration

The patient exits the cohort when encountering any of the following events:

- Other antidepressants except escitalopram

6.3.24 Comparator 2: new fluoxetine user with MPH

- **Cohort Entry Events**

People with continuous observation of 365 days before event may enter the cohort when observing any of the following:

- Any fluoxetine prescription for the first time in the person's history (index date}
- with age greater or equal to 18
- having at least 1 prescription of MPH at the date of index date

Limit cohort entry events to the earliest event per person.

- **Inclusion Criteria**

1. Patients with ADHD
   - Entry events having at least 1 diagnosis of ADHD for the first time in the person's history, starting anytime on or before the index date
2. Patients with depression
   - Entry events having at least 1 diagnosis of depression for the first time in the person's history, starting anytime on or before the index date.
3. Patients without antidepressants prior to the index date
   - Entry events having no prescription of the antidepressant, starting any time prior to the index date.

© 2024 Lee DY et al. *JAMA Network Open*

4. Patients without other ADHD medications
   - Entry events having no prescription of other ADHD medications

- **Cohort Exit**

  The cohort end date will be based on a continuous exposure to fluoxetine and MPH allowing 30 days between prescriptions, adding 30 days after exposure ends using the days supply and exposure end date for exposure duration

  The patient exits the cohort when encountering any of the following events:
  - Other antidepressants except fluoxetine

6.3.25 Comparator 3: new paroxetine user with MPH

- **Cohort Entry Events**

  People with continuous observation of 365 days before event may enter the cohort when observing any of the following:
  - Any paroxetine prescription for the first time in the person's history (index date}
  - with age greater or equal to 18
  - having at least 1 prescription of MPH at the date of index date

  Limit cohort entry events to the earliest event per person.

- **Inclusion Criteria**

  1. Patients with ADHD
     - Entry events having at least 1 diagnosis of ADHD for the first time in the person's history, starting anytime on or before the index date
  2. Patients with depression
     - Entry events having at least 1 diagnosis of depression for the first time in the person's history, starting anytime on or before the index date.
  3. Patients without antidepressants prior to the index date
     - Entry events having no prescription of the antidepressant, starting any time prior to the index date.
  4. Patients without other ADHD medications
     - Entry events having no prescription of other ADHD medications

- **Cohort Exit**

  The cohort end date will be based on a continuous exposure to paroxetine and MPH allowing 30 days between prescriptions, adding 30 days after exposure ends using the days supply and exposure end date for exposure duration

  The patient exits the cohort when encountering any of the following events:
  - Other antidepressants except paroxetine

6.3.26 Sensitivity comparator 1: new ADHD medication alone user

- **Cohort Entry Events**

  People with continuous observation of 365 days before event may enter the cohort when observing any of the following:

© 2024 Lee DY et al. *JAMA Network Open*

- Any ADHD medication prescription for the first time in the person's history (index date}
- with age greater or equal to 18

Limit cohort entry events to the earliest event per person.

- **Inclusion Criteria**

1. Patients with ADHD
    - Entry events having at least 1 diagnosis of ADHD for the first time in the person's history, starting anytime on or before the index date
2. Patients with depression
    - Entry events having at least 1 diagnosis of depression for the first time in the person's history, starting anytime on or before the index date.
3. Patients without antidepressants prior to the index date
    - Entry events having no prescription of the antidepressant, starting any time prior to the index date.

- **Cohort Exit**

The cohort end date will be based on a continuous exposure to ADHD medication allowing 30 days between prescriptions, adding 30 days after exposure ends using the days supply and exposure end date for exposure duration

The patient exits the cohort when encountering any of the following events:

- No censoring in this cohort

6.3.27 Sensitivity comparator 2: new escitalopram user with ADHD medication

- **Cohort Entry Events**

People with continuous observation of 365 days before event may enter the cohort when observing any of the following:

- Any escitalopram prescription for the first time in the person's history (index date}
- with age greater or equal to 18
- having at least 1 prescription of ADHD medication at the date of index date

Limit cohort entry events to the earliest event per person.

- **Inclusion Criteria**

1. Patients with ADHD
    - Entry events having at least 1 diagnosis of ADHD for the first time in the person's history, starting anytime on or before the index date
2. Patients with depression
    - Entry events having at least 1 diagnosis of depression for the first time in the person's history, starting anytime on or before the index date.
3. Patients without antidepressants prior to the index date
    - Entry events having no prescription of the antidepressant, starting any time prior to the index date.

- **Cohort Exit**

© 2024 Lee DY et al. *JAMA Network Open*

The cohort end date will be based on a continuous exposure to escitalopram and ADHD medication allowing 30 days between prescriptions, adding 30 days after exposure ends using the days supply and exposure end date for exposure duration

The patient exits the cohort when encountering any of the following events:

- Other antidepressants except escitalopram

6.3.28 Sensitivity comparator 3: new fluoxetine user with ADHD medication

- **Cohort Entry Events**

People with continuous observation of 365 days before event may enter the cohort when observing any of the following:

- Any fluoxetine prescription for the first time in the person's history (index date}
- with age greater or equal to 18
- having at least 1 prescription of ADHD medication at the date of index date

Limit cohort entry events to the earliest event per person.

- **Inclusion Criteria**

1. Patients with ADHD
   - Entry events having at least 1 diagnosis of ADHD for the first time in the person's history, starting anytime on or before the index date
2. Patients with depression
   - Entry events having at least 1 diagnosis of depression for the first time in the person's history, starting anytime on or before the index date.
3. Patients without antidepressants prior to the index date
   - Entry events having no prescription of the antidepressant, starting any time prior to the index date.

- **Cohort Exit**

The cohort end date will be based on a continuous exposure to fluoxetine and ADHD medication allowing 30 days between prescriptions, adding 30 days after exposure ends using the days supply and exposure end date for exposure duration

The patient exits the cohort when encountering any of the following events:

- Other antidepressants except fluoxetine

6.3.29 Sensitivity comparator 4: new paroxetine user with ADHD medication

- **Cohort Entry Events**

People with continuous observation of 365 days before event may enter the cohort when observing any of the following:

- Any paroxetine prescription for the first time in the person's history (index date}
- with age greater or equal to 18
- having at least 1 prescription of ADHD medication at the date of index date

Limit cohort entry events to the earliest event per person.

- **Inclusion Criteria**

© 2024 Lee DY et al. *JAMA Network Open*

1. Patients with ADHD
   - Entry events having at least 1 diagnosis of ADHD for the first time in the person's history, starting anytime on or before the index date
2. Patients with depression
   - Entry events having at least 1 diagnosis of depression for the first time in the person's history, starting anytime on or before the index date.
3. Patients without antidepressants prior to the index date
   - Entry events having no prescription of the antidepressant, starting any time prior to the index date.

- **Cohort Exit**

The cohort end date will be based on a continuous exposure to paroxetine and ADHD medication allowing 30 days between prescriptions, adding 30 days after exposure ends using the days supply and exposure end date for exposure duration

The patient exits the cohort when encountering any of the following events:

- Other antidepressants except paroxetine

6.3.30 Sensitivity comparator 5: new escitalopram user with MPH (allowing 30 days gap between two medications)

- **Cohort Entry Events**

People with continuous observation of 365 days before event may enter the cohort when observing any of the following:

- Any escitalopram prescription for the first time in the person's history (index date}
- with age greater or equal to 18
- having at least 1 prescription of MPH starting between 30 days before and 0 days after the index date

Limit cohort entry events to the earliest event per person.

- **Inclusion Criteria**

1. Patients with ADHD
   - Entry events having at least 1 diagnosis of ADHD for the first time in the person's history, starting anytime on or before the index date
2. Patients with depression
   - Entry events having at least 1 diagnosis of depression for the first time in the person's history, starting anytime on or before the index date.
3. Patients without antidepressants prior to the index date
   - Entry events having no prescription of the antidepressant, starting any time prior to the index date.
4. Patients without other ADHD medications
   - Entry events having no prescription of other ADHD medications

- **Cohort Exit**

© 2024 Lee DY et al. *JAMA Network Open*

The cohort end date will be based on a continuous exposure to escitalopram and MPH allowing 30 days between prescriptions, adding 30 days after exposure ends using the days supply and exposure end date for exposure duration

The patient exits the cohort when encountering any of the following events:

- Other antidepressants except escitalopram

6.3.31 Sensitivity comparator 6: new fluoxetine user with MPH (allowing 30 days gap between two medications)

- **Cohort Entry Events**

People with continuous observation of 365 days before event may enter the cohort when observing any of the following:

- Any fluoxetine prescription for the first time in the person's history (index date}
- with age greater or equal to 18
- having at least 1 prescription of MPH starting between 30 days before and 0 days after the index date

Limit cohort entry events to the earliest event per person.

- **Inclusion Criteria**

1. Patients with ADHD
   - Entry events having at least 1 diagnosis of ADHD for the first time in the person's history, starting anytime on or before the index date
2. Patients with depression
   - Entry events having at least 1 diagnosis of depression for the first time in the person's history, starting anytime on or before the index date.
3. Patients without antidepressants prior to the index date
   - Entry events having no prescription of the antidepressant, starting any time prior to the index date.
4. Patients without other ADHD medications
   - Entry events having no prescription of other ADHD medications

- **Cohort Exit**

The cohort end date will be based on a continuous exposure to fluoxetine and MPH allowing 30 days between prescriptions, adding 30 days after exposure ends using the days supply and exposure end date for exposure duration

The patient exits the cohort when encountering any of the following events:

- Other antidepressants except fluoxetine

6.3.32 Sensitivity comparator 7: new paroxetine user with MPH (allowing 30 days gap between two medications)

- **Cohort Entry Events**

People with continuous observation of 365 days before event may enter the cohort when observing any of the following:

- Any paroxetine prescription for the first time in the person's history (index date}

© 2024 Lee DY et al. *JAMA Network Open*

- with age greater or equal to 18
- having at least 1 prescription of MPH starting between 30 days before and 0 days after the index date

Limit cohort entry events to the earliest event per person.

- **Inclusion Criteria**

1. Patients with ADHD
   - Entry events having at least 1 diagnosis of ADHD for the first time in the person's history, starting anytime on or before the index date
2. Patients with depression
   - Entry events having at least 1 diagnosis of depression for the first time in the person's history, starting anytime on or before the index date.
3. Patients without antidepressants prior to the index date
   - Entry events having no prescription of the antidepressant, starting any time prior to the index date.
4. Patients without other ADHD medications
   - Entry events having no prescription of other ADHD medications

- **Cohort Exit**

The cohort end date will be based on a continuous exposure to paroxetine and MPH allowing 30 days between prescriptions, adding 30 days after exposure ends using the days supply and exposure end date for exposure duration

The patient exits the cohort when encountering any of the following events:

- Other antidepressants except paroxetine

6.3.33 Sensitivity comparator 8: new escitalopram user with ADHD medication (allowing 30 days gap between two medications)

- **Cohort Entry Events**

People with continuous observation of 365 days before event may enter the cohort when observing any of the following:

- Any escitalopram prescription for the first time in the person's history (index date}
- with age greater or equal to 18
- having at least 1 prescription of ADHD medication starting between 30 days before and 0 days after the index date

Limit cohort entry events to the earliest event per person.

- **Inclusion Criteria**

1. Patients with ADHD
   - Entry events having at least 1 diagnosis of ADHD for the first time in the person's history, starting anytime on or before the index date
2. Patients with depression
   - Entry events having at least 1 diagnosis of depression for the first time in the person's history, starting anytime on or before the index date.
3. Patients without antidepressants prior to the index date

© 2024 Lee DY et al. *JAMA Network Open*

- Entry events having no prescription of the antidepressant, starting any time prior to the index date.

- **Cohort Exit**

The cohort end date will be based on a continuous exposure to escitalopram and ADHD medication allowing 30 days between prescriptions, adding 30 days after exposure ends using the days supply and exposure end date for exposure duration

The patient exits the cohort when encountering any of the following events:

- Other antidepressants except escitalopram

6.3.34 Sensitivity comparator 9: new fluoxetine user with ADHD medication (allowing 30 days gap between two medications)

- **Cohort Entry Events**

People with continuous observation of 365 days before event may enter the cohort when observing any of the following:

- Any fluoxetine prescription for the first time in the person's history (index date}
- with age greater or equal to 18
- having at least 1 prescription of ADHD medication starting between 30 days before and 0 days after the index date

Limit cohort entry events to the earliest event per person.

- **Inclusion Criteria**

1. Patients with ADHD
   - Entry events having at least 1 diagnosis of ADHD for the first time in the person's history, starting anytime on or before the index date
2. Patients with depression
   - Entry events having at least 1 diagnosis of depression for the first time in the person's history, starting anytime on or before the index date.
3. Patients without antidepressants prior to the index date
   - Entry events having no prescription of the antidepressant, starting any time prior to the index date.

- **Cohort Exit**

The cohort end date will be based on a continuous exposure to fluoxetine and ADHD medication allowing 30 days between prescriptions, adding 30 days after exposure ends using the days supply and exposure end date for exposure duration

The patient exits the cohort when encountering any of the following events:

- Other antidepressants except fluoxetine

6.3.35 Sensitivity comparator 10: new paroxetine user with ADHD medication (allowing 30 days gap between two medications)

- **Cohort Entry Events**

People with continuous observation of 365 days before event may enter the cohort

© 2024 Lee DY et al. *JAMA Network Open*

when observing any of the following:

- Any paroxetine prescription for the first time in the person's history (index date}
- with age greater or equal to 18
- having at least 1 prescription of ADHD medication starting between 30 days before and 0 days after the index date

Limit cohort entry events to the earliest event per person.

- **Inclusion Criteria**

1. Patients with ADHD
   - Entry events having at least 1 diagnosis of ADHD for the first time in the person's history, starting anytime on or before the index date
2. Patients with depression
   - Entry events having at least 1 diagnosis of depression for the first time in the person's history, starting anytime on or before the index date.
3. Patients without antidepressants prior to the index date
   - Entry events having no prescription of the antidepressant, starting any time prior to the index date.

- **Cohort Exit**

The cohort end date will be based on a continuous exposure to paroxetine and ADHD medication allowing 30 days between prescriptions, adding 30 days after exposure ends using the days supply and exposure end date for exposure duration

The patient exits the cohort when encountering any of the following events:

- Other antidepressants except paroxetine

6.4 Outcomes

6.4.1 Primary Outcomes

- **Neuropsychiatric events**

Primary outcomes are neuropsychiatric events that include as below. All conditions could be detected by diagnostic codes.

Abnormal gait	ADHD hospitalisation	Agitation	Anorexia
Anxiety	Appetite loss	Delirium	Dizziness
Dystonia	Eating disorder	Epilepsy	Extrapyramidal symptoms
Gambling	Insomnia	Mania	Parkinsonism (drug-induced}
Schizophrenia related hospitalisation	Seizure	Sleep disorder	Substance abuse
Suicidal event	Tremor	Psychosis	

© 2024 Lee DY et al. *JAMA Network Open*

6.4.2 Secondary Outcomes

- **Secondary outcomes**

  Secondary outcomes are other safety events. Those are including each individual event as below. All conditions could be detected by diagnostic codes.

Abdominal pain	Accident	Acute respiratory failure	Anemia
Arrhythmia	Asthma outcome	Atrial fibrillation	Bleeding
Cardiomyopathy	Cerebrovascular disease	Chronic kidney disease	Diarrhea
Essential hypertension	Fatigue	Fever	Gynecomastia
Headache	Heart failure	Hyperlipidemia	Hyperprolactinemia
Hypo/hyperthyroidism	Hyponatremia	Hypotension	Ischemic heart disease
Liver disease	Myocardial infarction	Myocarditis	Nasopharyngitis
Nausea vomiting	Obesity	Osteoporosis	Thrombocytopenia
Traumatic injury	Type 2 diabetes mellitus	Upper respiratory tract infection or pneumonia	

6.5 Covariates

6.5.1 Propensity score covariates

Propensity scores (PS} will be used as an analytic strategy to reduce potential confounding due to imbalance between the treatment and comparator cohorts in baseline covariates[31]. The propensity score is the probability of a patient being classified in the treatment cohort vs. the comparator cohort, given a set of observed covariates. All covariates that occur in fewer than 0.1% of the persons between the treatment and comparator cohorts combined will be excluded prior to model fitting for computational efficiency. Large-scale propensity score matching methods will be applied[32].

The types of baseline covariates used to fit the propensity score model will be:

- Demographics
  - Sex
  - Age group (5-year bands}
  - Index year
- Aggregated conditions by SNOMED
  - In prior 365d
- Aggregated drugs codes by ATC/Ingredient levels
  - In prior 365d
  - Overlapping index date
- Charlson comorbidity index

Specific covariates which composed of exposures are excluded from the propensity score model.

© 2024 Lee DY et al. *JAMA Network Open*

6.5.2 Other variables

None

7 Data Sources

The analyses will be performed using the national ADHD dataset from the Health Insurance Review and Assessment Service of South Korea. This claim database includes data on Korean patients with a diagnosis of ADHD or a prescription for an ADHD drug from 2016 to February 2021. Since Korea's health insurance system is a single national insurance system, this database includes all citizens and includes information on diagnosis, prescription, examination, surgery, and treatment listed in the national reimbursement list.

The database has been transformed into the OMOP Common Data Model, version 5.3.1. The complete specification for OMOP Common Data Model, version 5.3.1 is available at: https://github.com/OHDSI/CommonDataModel.

8 Data Analysis Plan

8.1 Epidemiological consideration

8.1.1 Calculation of time-at-risk

- Primary analyses: As-treated risk window
  To avoid time-dependent bias, as-treated risk window is considered as the primary analysis outcome windows, of which time-at-risk starts on initiation of concomitant medications (antidepressant or ADHD medications} and ends when the treatment ends.
- Secondary analyses: As-treated risk window
  As-treated risk window is considered as the primary analysis outcome windows, of which time-at-risk starts on initiation of antidepressant treatment and ends when the treatment ends.
- Sensitivity analyses: Intention-to-treat risk window
  Risk window starts from 1 day to last observation after the index date.

8.1.2 Reducing bias

- Preventing bias from left censoring of data
  In order to prevent bias in the first visit and first prescription due to left censoring, the patients diagnosed and prescribed for the first year of the data period will not be used.
- Preventing bias from time-related settings
  In order to reduce time-related bias, sensitivity analysis will be additionally performed in addition to the main analysis. Sensitivity analyses according to time-at-risk setting (As-treated or

© 2024 Lee DY et al. *JAMA Network Open*

Intention-to-treat} and different gap durations between the concomitant drugs will be performed (e.g., between MPH and SSRI: 30 days, 0 days}.

- Preventing bias from reverse causality
  In order to avoid reverse causality due to outcome variables, especially related to symptoms, additional sensitivity analysis will be conducted in which symptomatic patients are removed and compared.

8.2 Model specification

In this study, we compare the treatment cohort with the comparator cohort for the hazards of outcome during the time-at-risk by applying a Cox proportional hazards model. A pre-specified *P*<0.05 was considered statistically significant for all two-sided tests.

The time-to-event of outcome among patients in the treatment and comparator cohorts is determined by calculating the number of days from the start of the time-at-risk window (the cohort start date}, until the earliest event among 1} the first occurrence of the outcome, 2} the end of the time-at-risk window, and 3} the end of the observation period that spans the time-at-risk start.

8.2.1 Statistical model for analyses

Propensity scores will be used as an analytic strategy to reduce potential confounding due to imbalance between the target and comparator cohorts in baseline covariates. The propensity score is estimated for each patient, using the predicted probability from a regularized logistic regression model, fit with a Laplace prior (LASSO} and the regularization hyperparameter selected by optimizing the likelihood in a 10-fold cross validation using 10 replications per fold, a starting variance of 0.01 and a tolerance of 2e-7. Covariates to be used in the propensity score model are listed in section 7.5.1.

- Primary analysis: After estimating the PS, stratification (PS stratification} will be performed. The number of strata will be 5. The outcome model will be fitted using an unconditional Cox regression, with only the treatment variable as predictor.

- Sensitivity analysis: After estimating the PS, matching (1:1 and 1: maximum [1: n] matching} will be performed. A caliper of 0.2 times the standard deviation of the propensity score distribution, and a greedy matching will be used. The outcome model will be fitted using an unconditioned Cox regression, with only the treatment variable as predictor.

8.3 Analyses to perform

The following analyses will be performed:

- 7 main comparisons
- 25 sensitivity comparisons
- 60 outcomes

© 2024 Lee DY et al. *JAMA Network Open*

- 2 types of outcomes (prevalent case and incident case}
- 2 time-at-risk definitions: As-treated risk window, Intention-to-treat risk window.
- 3 adjustments: PS stratification, 1:1 PS matching, 1: n PS matching

The total number of analyses is 23,040 (32 comparisons x 120 outcomes x 2 TAR x 3 PS methods}.

8.4 Output

Covariate balance will be summarized in tabular form by showing the mean value (percentage for categorical} for all baseline covariates in the target and comparator cohort, with the associated standardized mean difference computed for each covariate.

Once the propensity score model is fit, we will plot the propensity score distribution of the target and comparator cohorts to evaluate the comparability of the two cohorts. The plot will be scaled to the preference score, normalizing for any imbalance in cohort size. The covariates selected within the propensity score model, with associated coefficients will also be reported. A plot showing the preference score distributions for both cohorts after matching will be provided. Covariate balance will be evaluated by plotting the standardized mean difference of each covariate before propensity score matching against the standardized mean difference for each covariate after propensity score matching.

An attrition diagram (study flowchart} will be provided to detail the loss of patients from the original target cohort and comparator cohort to the subpopulations that remain after all design considerations have been applied.

The final outcome model, a Cox proportional hazards model, will be summarized by providing the hazards ratio and associated 95% confidence interval. The number of persons, amount of time-at-risk, and number of outcomes in each cohort will also be reported.

8.5 Quality control

We will evaluate the PS by

- Inspection of the fitted PS model for large coefficients (indicative of model-misspecification} and predictors that we cannot explain (post-hoc}.
- Inspection of the PS distribution.
- Evaluation of covariate balance after matching using the standardized difference in means between treatment and comparator cohort before and after matching. Standardized differences greater than 0.1 will be reported and investigated.

We will investigate the outcome model by

- Inspection of the fitted outcome model for large coefficients and predictors that we cannot explain (post-hoc}.

© 2024 Lee DY et al. *JAMA Network Open*

The error distribution estimated using the negative controls will be used to estimate residual bias after adjustments.

8.6 Strengths and Limitations of the Research Methods

Strength

- Cohort studies allow direct estimation of incidence rates following exposure of interest, and the new-user design can capture early events following treatment exposures while avoiding confounding from previous treatment effects. New use allows for a clear exposure index date.
- PS matching and full outcome models allow balancing on a large number of baseline potential confounders.

Limitations

- Even though many potential confounders will be included in this study, there may be residual bias due to unmeasured or mis-specified confounders.

9 Protection of Human Subjects

The study is using only de-identified data. Confidentiality of patient records will be maintained at all times. All study reports will contain aggregate data only and will not identify individual patients or physicians.

10 Plans for Disseminating and Communicating Study Results

The study protocol will be submitted for publication to an online repository before initiation of the study. Analytic codes will be posted on the online repository after completion of the study. At least one paper describing the study and its results will be written and submitted for publication to a peer-reviewed scientific journal.

11 References

1. Sayal K, Prasad V, Daley D, Ford T, Coghill D. ADHD in children and young people: prevalence, care pathways, and service provision. The Lancet Psychiatry. 2018;5(2}:175-86.
2. Adler LA, Faraone SV, Spencer TJ, Berglund P, Alperin S, Kessler RC. The structure of adult ADHD. International Journal of Methods in Psychiatric Research. 2017;26(1}:e1555.
3. Fredriksen M, Dahl AA, Martinsen EW, Klungsoyr O, Faraone SV, Peleikis DE. Childhood and persistent ADHD symptoms associated with educational failure and long-term occupational disability in adult ADHD. ADHD Attention Deficit and Hyperactivity Disorders. 2014;6(2}:87-99.
4. Katzman MA, Bilkey TS, Chokka PR, Fallu A, Klassen LJ. Adult ADHD and comorbid disorders: clinical implications of a dimensional approach. BMC Psychiatry. 2017;17(1}:302.
5. Tsang TW, Kohn MR, Efron D, Clarke SD, Clark CR, Lamb C, et al. Anxiety in Young People With ADHD: Clinical and Self-Report Outcomes. Journal of Attention Disorders. 2012;19(1}:18-26.

© 2024 Lee DY et al. *JAMA Network Open*

6. Eyre O, Riglin L, Leibenluft E, Stringaris A, Collishaw S, Thapar A. Irritability in ADHD: association with later depression symptoms. European Child & Adolescent Psychiatry. 2019;28(10}:1375-84.
7. van Emmerik-van Oortmerssen K, van de Glind G, van den Brink W, Smit F, Crunelle CL, Swets M, et al. Prevalence of attention-deficit hyperactivity disorder in substance use disorder patients: a meta-analysis and meta-regression analysis. Drug Alcohol Depend. 2012;122(1-2}:11-9.
8. Antshel KM, Zhang-James Y, Faraone SV. The comorbidity of ADHD and autism spectrum disorder. Expert Review of Neurotherapeutics. 2013;13(10}:1117-28.
9. Biederman J, Ball SW, Monuteaux MC, Mick E, Spencer TJ, McCreary M, et al. New Insights Into the Comorbidity Between ADHD and Major Depression in Adolescent and Young Adult Females. Journal of the American Academy of Child & Adolescent Psychiatry. 2008;47(4}:426-34.
10. Posner J, Siciliano F, Wang Z, Liu J, Sonuga-Barke E, Greenhill L. A multimodal MRI study of the hippocampus in medication-naive children with ADHD: What connects ADHD and depression? Psychiatry Research: Neuroimaging. 2014;224(2}:112-8.
11. Riglin L, Leppert B, Dardani C, Thapar AK, Rice F, O'Donovan MC, et al. ADHD and depression: investigating a causal explanation. Psychological Medicine. 2021;51(11}:1890-7.
12. Fenesy MC, Lee SS. Childhood ADHD and Executive Functioning: Unique Predictions of Early Adolescent Depression. Research on Child and Adolescent Psychopathology. 2021.
13. Blackman GL, Ostrander R, Herman KC. Children with ADHD and Depression: A Multisource, Multimethod Assessment of Clinical, Social, and Academic Functioning. Journal of Attention Disorders. 2005;8(4}:195-207.
14. Jick H, Kaye JA, Jick SS. Antidepressants and the Risk of Suicidal Behaviors. JAMA. 2004;292(3}:338-43.
15. Levy T, Kronenberg S, Crosbie J, Schachar RJ. Attention-deficit/hyperactivity disorder (ADHD} symptoms and suicidality in children: The mediating role of depression, irritability and anxiety symptoms. Journal of Affective Disorders. 2020;265:200-6.
16. Solmi M, Fornaro M, Ostinelli EG, Zangani C, Croatto G, Monaco F, et al. Safety of 80 antidepressants, antipsychotics, anti-attention-deficit/hyperactivity medications and mood stabilizers in children and adolescents with psychiatric disorders: a large scale systematic meta-review of 78 adverse effects. World Psychiatry. 2020;19(2}:214-32.
17. Sun S, Kuja-Halkola R, Faraone SV, D'Onofrio BM, Dalsgaard S, Chang Z, et al. Association of Psychiatric Comorbidity With the Risk of Premature Death Among Children and Adults With Attention-Deficit/Hyperactivity Disorder. JAMA Psychiatry. 2019;76(11}:1141-9.
18. Wolraich ML, Hagan JF, Jr, Allan C, Chan E, Davison D, Earls M, et al. Clinical Practice Guideline for the Diagnosis, Evaluation, and Treatment of Attention-Deficit/Hyperactivity Disorder in Children and Adolescents. Pediatrics. 2019;144(4}.
19. Cheung AH, Zuckerbrot RA, Jensen PS, Laraque D, Stein REK, GROUP G-PS, et al. Guidelines for Adolescent Depression in Primary Care (GLAD-PC}: Part II. Treatment and Ongoing Management. Pediatrics. 2018;141(3}.
20. Raman SR, Man KKC, Bahmanyar S, Berard A, Bilder S, Boukhris T, et al. Trends in attention-deficit hyperactivity disorder medication use: a retrospective observational study using population-based databases. The Lancet Psychiatry. 2018;5(10}:824-35.
21. Bachmann CJ, Wijlaars LP, Kalverdijk LJ, Burcu M, Glaeske G, Schuiling-Veninga CCM, et al. Trends in ADHD medication use in children and adolescents in five western countries, 2005-2012. European Neuropsychopharmacology. 2017;27(5}:484-93.
22. Schachar RJ, Tannock R, Cunningham C, Corkum PV. Behavioral, Situational, and Temporal Effects of Treatment of ADHD With Methylphenidate. Journal of the American Academy of Child & Adolescent Psychiatry. 1997;36(6}:754-63.

© 2024 Lee DY et al. *JAMA Network Open*

23. Sonnby K, Aslund C, Leppert J, Nilsson KW. Symptoms of ADHD and depression in a large adolescent population: Co-occurring symptoms and associations to experiences of sexual abuse. Nordic Journal of Psychiatry. 2011;65(5}:315-22.
24. Sakai C, Tsuji T, Nakai T, Namba Y, Mishima H, Fujiwara M, et al. Change in Antidepressant Use After Initiation of ADHD Medication in Japanese Adults with Comorbid Depression: A Real-World Database Analysis. Neuropsychiatr Dis Treat. 2021;17:3097-108.
25. Chen M-H, Pan T-L, Hsu J-W, Huang K-L, Su T-P, Li C-T, et al. Attention-deficit hyperactivity disorder comorbidity and antidepressant resistance among patients with major depression: A nationwide longitudinal study. European Neuropsychopharmacology. 2016;26(11}:1760-7.
26. Nevels RM, Weiss NH, Killebrew AE, Gontkovsky ST, editors. Methylphenidate and Its Underrecognized , Under-explained , and Serious Drug Interactions : A Review of the Literature with Heightened Concerns2013.
27. Ishii M, Tatsuzawa Y, Yoshino A, Nomura S. Serotonin syndrome induced by augmentation of SSRI with methylphenidate. Psychiatry and Clinical Neurosciences. 2008;62(2}:246-.
28. Methylphenidate/sertraline interaction. Reactions Weekly. 2008;1200(1}:24-.
29. Steiner H, Van Waes V, Marinelli M. Fluoxetine Potentiates Methylphenidate-Induced Gene Regulation in Addiction-Related Brain Regions: Concerns for Use of Cognitive Enhancers? Biological Psychiatry. 2010;67(6}:592-4.
30. Van Waes V, Ehrlich S, Beverley JA, Steiner H. Fluoxetine potentiation of methylphenidate-induced gene regulation in striatal output pathways: Potential role for 5-HT1B receptor. Neuropharmacology. 2015;89:77-86.
31. ROSENBAUM PR, RUBIN DB. The central role of the propensity score in observational studies for causal effects. Biometrika. 1983;70(1}:41-55.
32. Tian Y, Schuemie MJ, Suchard MA. Evaluating large-scale propensity score performance through real-world and synthetic data experiments. International journal of epidemiology. 2018;47(6}:2005-14.

© 2024 Lee DY et al. *JAMA Network Open*

13 Appendix: Code Set for Definitions

All codes are available in ATHENA (athena.ohdsi.org)

1. Attention-Deficit/Hyperactivity Disorder

Concept Id	Concept Name	Domain	Vocabulary	Excluded	Descendants	Mapped
438409	Attention deficit hyperactivity disorder	Condition	SNOMED	NO	YES	NO
4047120	Disorders of attention and motor control	Condition	SNOMED	NO	YES	NO

2. Methylphenidate

Concept Id	Concept Name	Domain	Vocabulary	Excluded	Descendants	Mapped
705944	methylphenidate	Drug	RxNorm	NO	YES	NO

3. Anti-ADHD drugs

Concept Id	Concept Name	Domain	Vocabulary	Excluded	Descendants	Mapped
705944	methylphenidate	Drug	RxNorm	NO	YES	NO
21604757	methylphenidate, oral	Drug	ATC	NO	YES	NO
742185	Atomoxetine	Drug	RxNorm	NO	YES	NO
21604762	Atomoxetine; oral	Drug	ATC	NO	YES	NO
21600398	Clonidine; systemic	Drug	ATC	NO	YES	NO

4. Other Anti-ADHD drugs for methylphenidate

Concept Id	Concept Name	Domain	Vocabulary	Excluded	Descendants	Mapped
742185	Atomoxetine	Drug	RxNorm	NO	YES	NO
21604762	Atomoxetine; oral	Drug	ATC	NO	YES	NO
21600398	Clonidine; systemic	Drug	ATC	NO	YES	NO

5. Depression

Concept Id	Concept Name	Domain	Vocabulary	Excluded	Descendants	Mapped
440383	Depressive disorder	Condition	SNOMED	NO	YES	NO
442306	Adjustment disorder with depressed mood	Condition	SNOMED	NO	YES	NO
4175329	Organic mood disorder of depressed type	Condition	SNOMED	NO	YES	NO
436665	Bipolar disorder	Condition	SNOMED	YES	YES	NO

6. Antidepressant

Concept ID	Concept Name	Domain	Vocabulary	Exclude	Descendants	Mapped
710062	amitriptyline	Drug	RxNorm	NO	YES	NO
21604696	amitriptyline; systemic	Drug	ATC	NO	YES	NO
750982	bupropion	Drug	RxNorm	NO	YES	NO
21604741	bupropion; oral	Drug	ATC	NO	YES	NO
797617	citalopram	Drug	RxNorm	NO	YES	NO
21604712	citalopram; systemic	Drug	ATC	NO	YES	NO
717607	desvenlafaxine	Drug	RxNorm	NO	YES	NO

© 2024 Lee DY et al. *JAMA Network Open*

21604751	desvenlafaxine; oral	Drug	ATC	NO	YES	NO
738156	doxepin	Drug	RxNorm	NO	YES	NO
21604699	doxepin; systemic	Drug	ATC	NO	YES	NO
715259	duloxetine	Drug	RxNorm	NO	YES	NO
21604749	duloxetine; oral	Drug	ATC	NO	YES	NO
715939	escitalopram	Drug	RxNorm	NO	YES	NO
21604718	escitalopram; oral	Drug	ATC	NO	YES	NO
755695	fluoxetine	Drug	RxNorm	NO	YES	NO
21604711	fluoxetine; oral	Drug	ATC	NO	YES	NO
778268	imipramine	Drug	RxNorm	NO	YES	NO
21604689	imipramine; systemic	Drug	ATC	NO	YES	NO
725131	mirtazapine	Drug	RxNorm	NO	YES	NO
21604740	mirtazapine; oral	Drug	ATC	NO	YES	NO
721724	nortriptyline	Drug	RxNorm	NO	YES	NO
21604697	nortriptyline; systemic	Drug	ATC	NO	YES	NO
722031	paroxetine	Drug	RxNorm	NO	YES	NO
21604713	paroxetine; oral	Drug	ATC	NO	YES	NO
739138	sertraline	Drug	RxNorm	NO	YES	NO
21604714	sertraline; oral	Drug	ATC	NO	YES	NO
21604693	trimipramine; systemic	Drug	ATC	NO	YES	NO
743670	venlafaxine	Drug	RxNorm	NO	YES	NO
21604745	venlafaxine; oral	Drug	ATC	NO	YES	NO

7. Selective serotonin reuptake inhibitor

Concept Id	Concept Name	Domain	Vocabulary	Excluded	Descendants	Mapped
797617	citalopram	Drug	RxNorm	NO	YES	NO
21604712	citalopram; systemic	Drug	ATC	NO	YES	NO
715939	escitalopram	Drug	RxNorm	NO	YES	NO
21604718	escitalopram; oral	Drug	ATC	NO	YES	NO
755695	fluoxetine	Drug	RxNorm	NO	YES	NO
21604711	fluoxetine; oral	Drug	ATC	NO	YES	NO
739138	sertraline	Drug	RxNorm	NO	YES	NO
21604714	sertraline; oral	Drug	ATC	NO	YES	NO
722031	paroxetine	Drug	RxNorm	NO	YES	NO
21604713	paroxetine; oral	Drug	ATC	NO	YES	NO

8. Escitalopram

Concept Id	Concept Name	Domain	Vocabulary	Excluded	Descendants	Mapped
715939	escitalopram	Drug	RxNorm	NO	YES	NO
21604718	escitalopram; oral	Drug	ATC	NO	YES	NO

9. Fluoxetine

Concept Id	Concept Name	Domain	Vocabulary	Excluded	Descendants	Mapped
755695	fluoxetine	Drug	RxNorm	NO	YES	NO
21604711	fluoxetine; oral	Drug	ATC	NO	YES	NO

© 2024 Lee DY et al. *JAMA Network Open*

10. Sertraline

Concept Id	Concept Name	Domain	Vocabulary	Excluded	Descendants	Mapped
739138	sertraline	Drug	RxNorm	NO	YES	NO
21604714	sertraline; oral	Drug	ATC	NO	YES	NO

11. Paroxetine

Concept Id	Concept Name	Domain	Vocabulary	Excluded	Descendants	Mapped
722031	paroxetine	Drug	RxNorm	NO	YES	NO
21604713	paroxetine; oral	Drug	ATC	NO	YES	NO

12. Other Antidepressant except SSRIs

Concept ID	Concept Name	Domain	Vocabulary	Exclude	Descendants	Mapped
710062	amitriptyline	Drug	RxNorm	NO	YES	NO
21604696	amitriptyline; systemic	Drug	ATC	NO	YES	NO
750982	bupropion	Drug	RxNorm	NO	YES	NO
21604741	bupropion; oral	Drug	ATC	NO	YES	NO
797617	citalopram	Drug	RxNorm	NO	YES	NO
21604712	citalopram; systemic	Drug	ATC	NO	YES	NO
717607	desvenlafaxine	Drug	RxNorm	NO	YES	NO
21604751	desvenlafaxine; oral	Drug	ATC	NO	YES	NO
738156	doxepin	Drug	RxNorm	NO	YES	NO
21604699	doxepin; systemic	Drug	ATC	NO	YES	NO
715259	duloxetine	Drug	RxNorm	NO	YES	NO
21604749	duloxetine; oral	Drug	ATC	NO	YES	NO
778268	imipramine	Drug	RxNorm	NO	YES	NO
21604689	imipramine; systemic	Drug	ATC	NO	YES	NO
725131	mirtazapine	Drug	RxNorm	NO	YES	NO
21604740	mirtazapine; oral	Drug	ATC	NO	YES	NO
721724	nortriptyline	Drug	RxNorm	NO	YES	NO
21604697	nortriptyline; systemic	Drug	ATC	NO	YES	NO
21604693	trimipramine; systemic	Drug	ATC	NO	YES	NO
743670	venlafaxine	Drug	RxNorm	NO	YES	NO
21604745	venlafaxine; oral	Drug	ATC	NO	YES	NO

13. Other Antidepressant except escitalopram

Concept ID	Concept Name	Domain	Vocabulary	Exclude	Descendants	Mapped
710062	amitriptyline	Drug	RxNorm	NO	YES	NO
21604696	amitriptyline; systemic	Drug	ATC	NO	YES	NO
750982	bupropion	Drug	RxNorm	NO	YES	NO
21604741	bupropion; oral	Drug	ATC	NO	YES	NO
797617	citalopram	Drug	RxNorm	NO	YES	NO
21604712	citalopram; systemic	Drug	ATC	NO	YES	NO
717607	desvenlafaxine	Drug	RxNorm	NO	YES	NO
21604751	desvenlafaxine; oral	Drug	ATC	NO	YES	NO

© 2024 Lee DY et al. *JAMA Network Open*

738156	doxepin	Drug	RxNorm	NO	YES	NO
21604699	doxepin; systemic	Drug	ATC	NO	YES	NO
715259	duloxetine	Drug	RxNorm	NO	YES	NO
21604749	duloxetine; oral	Drug	ATC	NO	YES	NO
755695	fluoxetine	Drug	RxNorm	NO	YES	NO
21604711	fluoxetine; oral	Drug	ATC	NO	YES	NO
778268	imipramine	Drug	RxNorm	NO	YES	NO
21604689	imipramine; systemic	Drug	ATC	NO	YES	NO
725131	mirtazapine	Drug	RxNorm	NO	YES	NO
21604740	mirtazapine; oral	Drug	ATC	NO	YES	NO
721724	nortriptyline	Drug	RxNorm	NO	YES	NO
21604697	nortriptyline; systemic	Drug	ATC	NO	YES	NO
722031	paroxetine	Drug	RxNorm	NO	YES	NO
21604713	paroxetine; oral	Drug	ATC	NO	YES	NO
739138	sertraline	Drug	RxNorm	NO	YES	NO
21604714	sertraline; oral	Drug	ATC	NO	YES	NO
21604693	trimipramine; systemic	Drug	ATC	NO	YES	NO
743670	venlafaxine	Drug	RxNorm	NO	YES	NO
21604745	venlafaxine; oral	Drug	ATC	NO	YES	NO

14. Other Antidepressant except fluoxetine

Concept ID	Concept Name	Domain	Vocabulary	Exclude	Descendants	Mapped
710062	amitriptyline	Drug	RxNorm	NO	YES	NO
21604696	amitriptyline; systemic	Drug	ATC	NO	YES	NO
750982	bupropion	Drug	RxNorm	NO	YES	NO
21604741	bupropion; oral	Drug	ATC	NO	YES	NO
797617	citalopram	Drug	RxNorm	NO	YES	NO
21604712	citalopram; systemic	Drug	ATC	NO	YES	NO
717607	desvenlafaxine	Drug	RxNorm	NO	YES	NO
21604751	desvenlafaxine; oral	Drug	ATC	NO	YES	NO
738156	doxepin	Drug	RxNorm	NO	YES	NO
21604699	doxepin; systemic	Drug	ATC	NO	YES	NO
715259	duloxetine	Drug	RxNorm	NO	YES	NO
21604749	duloxetine; oral	Drug	ATC	NO	YES	NO
715939	escitalopram	Drug	RxNorm	NO	YES	NO
21604718	escitalopram; oral	Drug	ATC	NO	YES	NO
778268	imipramine	Drug	RxNorm	NO	YES	NO
21604689	imipramine; systemic	Drug	ATC	NO	YES	NO
725131	mirtazapine	Drug	RxNorm	NO	YES	NO
21604740	mirtazapine; oral	Drug	ATC	NO	YES	NO
721724	nortriptyline	Drug	RxNorm	NO	YES	NO
21604697	nortriptyline; systemic	Drug	ATC	NO	YES	NO
722031	paroxetine	Drug	RxNorm	NO	YES	NO
21604713	paroxetine; oral	Drug	ATC	NO	YES	NO
739138	sertraline	Drug	RxNorm	NO	YES	NO
21604714	sertraline; oral	Drug	ATC	NO	YES	NO
21604693	trimipramine; systemic	Drug	ATC	NO	YES	NO
743670	venlafaxine	Drug	RxNorm	NO	YES	NO
21604745	venlafaxine; oral	Drug	ATC	NO	YES	NO

© 2024 Lee DY et al. *JAMA Network Open*

15. Other Antidepressant except sertraline

Concept ID	Concept Name	Domain	Vocabulary	Exclude	Descendants	Mapped
710062	amitriptyline	Drug	RxNorm	NO	YES	NO
21604696	amitriptyline; systemic	Drug	ATC	NO	YES	NO
750982	bupropion	Drug	RxNorm	NO	YES	NO
21604741	bupropion; oral	Drug	ATC	NO	YES	NO
797617	citalopram	Drug	RxNorm	NO	YES	NO
21604712	citalopram; systemic	Drug	ATC	NO	YES	NO
717607	desvenlafaxine	Drug	RxNorm	NO	YES	NO
21604751	desvenlafaxine; oral	Drug	ATC	NO	YES	NO
738156	doxepin	Drug	RxNorm	NO	YES	NO
21604699	doxepin; systemic	Drug	ATC	NO	YES	NO
715259	duloxetine	Drug	RxNorm	NO	YES	NO
21604749	duloxetine; oral	Drug	ATC	NO	YES	NO
715939	escitalopram	Drug	RxNorm	NO	YES	NO
21604718	escitalopram; oral	Drug	ATC	NO	YES	NO
755695	fluoxetine	Drug	RxNorm	NO	YES	NO
21604711	fluoxetine; oral	Drug	ATC	NO	YES	NO
778268	imipramine	Drug	RxNorm	NO	YES	NO
21604689	imipramine; systemic	Drug	ATC	NO	YES	NO
725131	mirtazapine	Drug	RxNorm	NO	YES	NO
21604740	mirtazapine; oral	Drug	ATC	NO	YES	NO
721724	nortriptyline	Drug	RxNorm	NO	YES	NO
21604697	nortriptyline; systemic	Drug	ATC	NO	YES	NO
722031	paroxetine	Drug	RxNorm	NO	YES	NO
21604713	paroxetine; oral	Drug	ATC	NO	YES	NO
21604693	trimipramine; systemic	Drug	ATC	NO	YES	NO
743670	venlafaxine	Drug	RxNorm	NO	YES	NO
21604745	venlafaxine; oral	Drug	ATC	NO	YES	NO

16. Other Antidepressant except paroxetine

Concept ID	Concept Name	Domain	Vocabulary	Exclude	Descendants	Mapped
710062	amitriptyline	Drug	RxNorm	NO	YES	NO
21604696	amitriptyline; systemic	Drug	ATC	NO	YES	NO
750982	bupropion	Drug	RxNorm	NO	YES	NO
21604741	bupropion; oral	Drug	ATC	NO	YES	NO
797617	citalopram	Drug	RxNorm	NO	YES	NO
21604712	citalopram; systemic	Drug	ATC	NO	YES	NO
717607	desvenlafaxine	Drug	RxNorm	NO	YES	NO
21604751	desvenlafaxine; oral	Drug	ATC	NO	YES	NO
738156	doxepin	Drug	RxNorm	NO	YES	NO
21604699	doxepin; systemic	Drug	ATC	NO	YES	NO
715259	duloxetine	Drug	RxNorm	NO	YES	NO
21604749	duloxetine; oral	Drug	ATC	NO	YES	NO
715939	escitalopram	Drug	RxNorm	NO	YES	NO
21604718	escitalopram; oral	Drug	ATC	NO	YES	NO
755695	fluoxetine	Drug	RxNorm	NO	YES	NO
21604711	fluoxetine; oral	Drug	ATC	NO	YES	NO
778268	imipramine	Drug	RxNorm	NO	YES	NO

© 2024 Lee DY et al. *JAMA Network Open*

21604689	imipramine; systemic	Drug	ATC	NO	YES	NO
725131	mirtazapine	Drug	RxNorm	NO	YES	NO
21604740	mirtazapine; oral	Drug	ATC	NO	YES	NO
721724	nortriptyline	Drug	RxNorm	NO	YES	NO
21604697	nortriptyline; systemic	Drug	ATC	NO	YES	NO
739138	sertraline	Drug	RxNorm	NO	YES	NO
21604714	sertraline; oral	Drug	ATC	NO	YES	NO
21604693	trimipramine; systemic	Drug	ATC	NO	YES	NO
743670	venlafaxine	Drug	RxNorm	NO	YES	NO
21604745	venlafaxine; oral	Drug	ATC	NO	YES	NO

17. Abdominal pain

Concept Id	Concept Name	Domain	Vocabulary	Excluded	Descendants	Mapped
200219	Abdominal pain	Condition	SNOMED	NO	YES	NO

18. Abnormal gait

Concept Id	Concept Name	Domain	Vocabulary	Excluded	Descendants	Mapped
437643	Abnormal gait	Observation	SNOMED	NO	YES	NO

19. Accident

Concept Id	Concept Name	Domain	Vocabulary	Excluded	Descendants	Mapped
432532	Accidental event	Observation	SNOMED	NO	YES	NO

20. Acute respiratory failure

Concept Id	Concept Name	Domain	Vocabulary	Excluded	Descendants	Mapped
319049	Acute respiratory failure	Condition	SNOMED	NO	YES	NO

21. ADHD hospitalisation

Concept Id	Concept Name	Domain	Vocabulary	Excluded	Descendants	Mapped
4047120	Disorder of attention and motor control	Condition	SNOMED	NO	YES	NO
438409	Attention deficit hyperactivity disorder	Condition	SNOMED	NO	YES	NO

22. Agitation

Concept Id	Concept Name	Domain	Vocabulary	Excluded	Descendants	Mapped
4168212	Restlessness and agitation	Condition	SNOMED	NO	YES	NO

© 2024 Lee DY et al. *JAMA Network Open*

23. Anemia

Concept Id	Concept Name	Domain	Vocabulary	Excluded	Descendants	Mapped
439777	Anemia	Condition	SNOMED	NO	YES	NO

24. Anorexia

Concept Id	Concept Name	Domain	Vocabulary	Excluded	Descendants	Mapped
436675	Anorexia nervosa	Condition	SNOMED	NO	YES	NO

25. Anxiety

Concept ID	Concept Name	Domain	Vocabulary	Exclude	Descendants	Mapped
442077	Anxiety disorder	Condition	SNOMED	NO	YES	NO
37109206	Anxiety disorder caused by drug	Condition	SNOMED	NO	YES	NO
4199892	Anxiety disorder due to a general medical condition	Condition	SNOMED	NO	YES	NO
434613	Generalized anxiety disorder	Condition	SNOMED	NO	YES	NO
4338031	Mixed anxiety and depressive disorder	Condition	SNOMED	NO	YES	NO
381537	Organic anxiety disorder	Condition	SNOMED	NO	YES	NO
436074	Panic disorder	Condition	SNOMED	NO	YES	NO
4304010	Phobic disorder	Condition	SNOMED	NO	YES	NO

26. Appetite loss

Concept Id	Concept Name	Domain	Vocabulary	Excluded	Descendants	Mapped
442165	Loss of appetite	Observation	SNOMED	NO	YES	NO

27. Arrhythmia

Concept Id	Concept Name	Domain	Vocabulary	Excluded	Descendants	Mapped
4068155	Atrial arrhythmia	Condition	SNOMED	NO	YES	NO
44784217	Cardiac arrhythmia	Condition	SNOMED	NO	YES	NO
4111552	Re-entry ventricular arrhythmia	Condition	SNOMED	NO	YES	NO
4248028	Supraventricular arrhythmia	Condition	SNOMED	NO	YES	NO
315643	Tachyarrhythmia	Condition	SNOMED	NO	YES	NO
444070	Tachycardia	Condition	SNOMED	NO	YES	NO
4185572	Ventricular arrhythmia	Condition	SNOMED	NO	YES	NO

28. Asthma

Concept Id	Concept Name	Domain	Vocabulary	Excluded	Descendants	Mapped

© 2024 Lee DY et al. *JAMA Network Open*

317009	Asthma	Condition	SNOMED	NO	YES	NO

29. Atrial fibrillation

Concept Id	Concept Name	Domain	Vocabulary	Excluded	Descendants	Mapped
4141360	Chronic atrial fibrillation	Condition	SNOMED	NO	NO	NO
4154290	Paroxysmal atrial fibrillation	Condition	SNOMED	NO	NO	NO
4232697	Persistent atrial fibrillation	Condition	SNOMED	NO	NO	NO
36714994	Typical atrial flutter	Condition	SNOMED	NO	NO	NO

30. Bleeding

Concept Id	Concept Name	Domain	Vocabulary	Excluded	Descendants	Mapped
437312	Bleeding	Condition	SNOMED	NO	YES	NO

31. Cardiomyopathy

Concept Id	Concept Name	Domain	Vocabulary	Excluded	Descendants	Mapped
321319	Cardiomyopathy	Condition	SNOMED	NO	NO	NO
320746	Cardiomyopathy associated with another disorder	Condition	SNOMED	NO	NO	NO
4163710	Dilated cardiomyopathy	Condition	SNOMED	NO	NO	NO
318773	Dilated cardiomyopathy secondary to alcohol	Condition	SNOMED	NO	NO	NO
4124693	Hypertrophic cardiomyopathy	Condition	SNOMED	NO	NO	NO
316428	Hypertrophic obstructive cardiomyopathy	Condition	SNOMED	NO	NO	NO
321320	Myocardial degeneration	Condition	SNOMED	NO	NO	NO
4190773	Restrictive cardiomyopathy	Condition	SNOMED	NO	NO	NO

32. Cerebrovascular disease

Concept Id	Concept Name	Domain	Vocabulary	Excluded	Descendants	Mapped
381591	Cerebrovascular disease	Condition	SNOMED	NO	YES	NO

33. Chronic kidney disease

Concept Id	Concept Name	Domain	Vocabulary	Excluded	Descendants	Mapped
46271022	Chronic kidney disease	Condition	SNOMED	NO	YES	NO

34. Constipation

Concept Id	Concept Name	Domain	Vocabulary	Excluded	Descendants	Mapped
75860	Constipation	Condition	SNOMED	NO	YES	NO

© 2024 Lee DY et al. *JAMA Network Open*

35. Coronary heart disease

Concept ID	Concept Name	Domain	Vocabulary	Exclude	Descendants	Mapped
435081	Abnormal findings diagnostic imaging heart+coronary circulat	Condition	SNOMED	NO	NO	NO
43020480	Acquired coronary artery fistula	Condition	SNOMED	NO	NO	NO
321109	Congenital anomaly of coronary artery	Condition	SNOMED	NO	NO	NO
317576	Coronary arteriosclerosis	Condition	SNOMED	NO	NO	NO
42872402	Coronary arteriosclerosis in native artery	Condition	SNOMED	NO	NO	NO
42537730	Coronary artery graft present	Condition	SNOMED	NO	NO	NO
4127089	Coronary artery spasm	Condition	SNOMED	NO	NO	NO
4108215	Coronary thrombosis not resulting in myocardial infarction	Condition	SNOMED	NO	NO	NO

36. Delirium

Concept Id	Concept Name	Domain	Vocabulary	Excluded	Descendants	Mapped
373995	Delirium	Condition	SNOMED	NO	YES	NO

37. Diarrhea

Concept Id	Concept Name	Domain	Vocabulary	Excluded	Descendants	Mapped
196523	Diarrhea	Condition	SNOMED	NO	YES	NO

38. Dizziness

Concept Id	Concept Name	Domain	Vocabulary	Excluded	Descendants	Mapped
4223938	Dizziness	Condition	SNOMED	NO	YES	NO

39. Dystonia

Concept Id	Concept Name	Domain	Vocabulary	Excluded	Descendants	Mapped
375800	Dystonia	Condition	SNOMED	NO	YES	NO

40. Eating disorder

Concept Id	Concept Name	Domain	Vocabulary	Excluded	Descendants	Mapped
439002	Eating disorder	Condition	SNOMED	NO	YES	NO

41. Epilepsy

Concept Id	Concept Name	Domain	Vocabulary	Excluded	Descendants	Mapped
380378	Epilepsy	Condition	SNOMED	NO	YES	NO

© 2024 Lee DY et al. *JAMA Network Open*

42. Extrapyramidal symptoms

Concept Id	Concept Name	Domain	Vocabulary	Excluded	Descendants	Mapped
443782	Tremor	Condition	SNOMED	NO	YES	NO
4171569	Parkinsonism due to drug	Condition	SNOMED	NO	YES	NO
374013	Secondary parkinsonism	Condition	SNOMED	NO	YES	NO
375800	Dystonia	Condition	SNOMED	NO	YES	NO

43. Essential hypertension

Concept Id	Concept Name	Domain	Vocabulary	Excluded	Descendants	Mapped
320128	Essential hypertension	Condition	SNOMED	NO	YES	NO

44. Fatigue

Concept Id	Concept Name	Domain	Vocabulary	Excluded	Descendants	Mapped
439926	Malaise and fatigue	Condition	SNOMED	NO	YES	NO

45. Fever

Concept Id	Concept Name	Domain	Vocabulary	Excluded	Descendants	Mapped
437663	Fever	Condition	SNOMED	NO	YES	NO

46. Gambling

Concept Id	Concept Name	Domain	Vocabulary	Excluded	Descendants	Mapped
4023166	Gambling	Observation	SNOMED	NO	YES	NO
436959	Compulsive gambling	Condition	SNOMED	NO	YES	NO

47. Gynecomastia

Concept Id	Concept Name	Domain	Vocabulary	Excluded	Descendants	Mapped
4168447	Gynecomastia	Condition	SNOMED	NO	YES	NO
79884	Galactorrhea not associated with childbirth	Condition	SNOMED	NO	YES	NO

48. Headache

Concept Id	Concept Name	Domain	Vocabulary	Excluded	Descendants	Mapped
378253	Headache	Condition	SNOMED	NO	YES	NO

49. Heart failure

Concept Id	Concept Name	Domain	Vocabulary	Excluded	Descendants	Mapped
316139	Heart failure	Condition	SNOMED	NO	YES	NO

© 2024 Lee DY et al. *JAMA Network Open*

50. Hyperlipidemia

Concept Id	Concept Name	Domain	Vocabulary	Excluded	Descendants	Mapped
437312	Bleeding	Condition	SNOMED	NO	YES	NO

51. Hyperprolactinemia

Concept Id	Concept Name	Domain	Vocabulary	Excluded	Descendants	Mapped
4030186	Hyperprolactinemia	Condition	SNOMED	NO	YES	NO

52. hypo and hyperthyroidism

Concept Id	Concept Name	Domain	Vocabulary	Excluded	Descendants	Mapped
140673	Hypothyroidism	Condition	SNOMED	NO	YES	NO
4142479	Hyperthyroidism	Condition	SNOMED	NO	YES	NO

53. Hyponatremia

Concept Id	Concept Name	Domain	Vocabulary	Excluded	Descendants	Mapped
435515	Hypo-osmolality and or hyponatremia	Condition	SNOMED	NO	YES	NO

54. Hypotension

Concept Id	Concept Name	Domain	Vocabulary	Excluded	Descendants	Mapped
319041	Orthostatic hypotension	Condition	SNOMED	NO	YES	NO
4112334	Idiopathic hypotension	Condition	SNOMED	NO	YES	NO
4120275	Drug-induced hypotension	Condition	SNOMED	NO	YES	NO

55. Insomnia

Concept Id	Concept Name	Domain	Vocabulary	Excluded	Descendants	Mapped
4102985	Nonorganic insomnia	Condition	SNOMED	NO	YES	NO

56. Ischemic heart disease

Concept Id	Concept Name	Domain	Vocabulary	Excluded	Descendants	Mapped
4185932	Ischemic heart disease	Condition	SNOMED	NO	YES	NO

57. Liver disease

Concept Id	Concept Name	Domain	Vocabulary	Excluded	Descendants	Mapped
4212540	Chronic liver disease	Condition	SNOMED	NO	YES	NO
4243475	Acute hepatitis	Condition	SNOMED	NO	YES	NO

© 2024 Lee DY et al. *JAMA Network Open*

58. Mania

Concept Id	Concept Name	Domain	Vocabulary	Excluded	Descendants	Mapped
35610112	Mania with psychotic features	Condition	SNOMED	NO	YES	NO
4333677	Mania	Condition	SNOMED	NO	YES	NO

59. Myocardial infarction

Concept Id	Concept Name	Domain	Vocabulary	Excluded	Descendants	Mapped
4329847	Myocardial infarction	Condition	SNOMED	NO	YES	NO

60. Myocarditis

Concept Id	Concept Name	Domain	Vocabulary	Excluded	Descendants	Mapped
314383	Myocarditis	Condition	SNOMED	NO	YES	NO

61. Nasopharyngitis

Concept Id	Concept Name	Domain	Vocabulary	Excluded	Descendants	Mapped
4197268	Nasopharyngitis	Condition	SNOMED	NO	YES	NO

62. Nausea and vomiting

Concept Id	Concept Name	Domain	Vocabulary	Excluded	Descendants	Mapped
31967	Nausea	Condition	SNOMED	NO	YES	NO
441408	Vomiting	Condition	SNOMED	NO	YES	NO

63. Obesity

Concept Id	Concept Name	Domain	Vocabulary	Excluded	Descendants	Mapped
433736	Obesity	Condition	SNOMED	NO	YES	NO

64. Osteoporosis

Concept Id	Concept Name	Domain	Vocabulary	Excluded	Descendants	Mapped
80502	Osteoporosis	Condition	SNOMED	NO	YES	NO

65. Parkinsonism (Drug induced}

Concept Id	Concept Name	Domain	Vocabulary	Excluded	Descendants	Mapped
374013	Secondary parkinsonism	Condition	SNOMED	NO	YES	NO
4171569	Parkinsonism due to drug	Condition	SNOMED	NO	YES	NO

© 2024 Lee DY et al. *JAMA Network Open*

66. Psychosis

Concept Id	Concept Name	Domain	Vocabulary	Excluded	Descendants	Mapped
436073	Psychotic disorder	Condition	SNOMED	NO	YES	NO

67. Schizophrenia related hospitalisation

Concept ID	Concept Name	Domain	Vocabulary	Exclude	Descendants	Mapped
4335169	Acute transient psychotic disorder	Condition	SNOMED	NO	YES	NO
432590	Delusional disorder	Condition	SNOMED	NO	YES	NO
4286201	Schizoaffective disorder	Condition	SNOMED	NO	YES	NO
435783	Schizophrenia	Condition	SNOMED	NO	YES	NO
434010	Schizotypal personality disorder	Condition	SNOMED	NO	YES	NO
35207135	Shared psychotic disorder	Condition	ICD10CM	NO	YES	NO
37117049	Substance induced psychotic disorder	Condition	SNOMED	NO	YES	NO

68. Seizure

Concept Id	Concept Name	Domain	Vocabulary	Excluded	Descendants	Mapped
377091	Seizure	Condition	SNOMED	NO	YES	NO

69. Sleep disorder

Concept Id	Concept Name	Domain	Vocabulary	Excluded	Descendants	Mapped
435524	Sleep disorder	Condition	SNOMED	NO	YES	NO

70. Substance abuse

Concept Id	Concept Name	Domain	Vocabulary	Excluded	Descendants	Mapped
4279309	Substance abuse	Condition	SNOMED	NO	YES	NO

71. Suicidal event

Concept Id	Concept Name	Domain	Vocabulary	Excluded	Descendants	Mapped
4219484	Suicide attempt	Observation	SNOMED	NO	YES	NO
4092411	Self-injurious behavior	Condition	SNOMED	NO	YES	NO
4304690	Intentionally harming self	Observation	SNOMED	NO	YES	NO
439235	Self inflicted injury	Condition	SNOMED	NO	YES	NO
435446	Late effect of self inflicted injury	Condition	SNOMED	NO	YES	NO
4152376	Intentional self poisoning	Condition	SNOMED	NO	YES	NO
4152408	H/O: deliberate self harm	Condition	SNOMED	NO	YES	NO
4075235	Drowning self	Condition	SNOMED	NO	YES	NO

© 2024 Lee DY et al. *JAMA Network Open*

72. Thrombocytopenia

Concept Id	Concept Name	Domain	Vocabulary	Excluded	Descendants	Mapped
40321716	Secondary thrombocytopenia	Condition	SNOMED	NO	YES	NO
441264	Primary thrombocytopenia	Condition	SNOMED	NO	YES	NO

73. Traumatic injury

Concept Id	Concept Name	Domain	Vocabulary	Excluded	Descendants	Mapped
440921	Traumatic injury	Condition	SNOMED	NO	YES	NO

74. Tremor

Concept Id	Concept Name	Domain	Vocabulary	Excluded	Descendants	Mapped
443782	Tremor	Condition	SNOMED	NO	YES	NO

75. Type 2 diabetes mellitus

Concept Id	Concept Name	Domain	Vocabulary	Excluded	Descendants	Mapped
201826	Type 2 diabetes mellitus	Condition	SNOMED	NO	YES	NO

76. Upper respiratory tract infection and pneumonia

Concept Id	Concept Name	Domain	Vocabulary	Excluded	Descendants	Mapped
4181583	Upper respiratory infection	Condition	SNOMED	NO	YES	NO
255848	Pneumonia	Condition	SNOMED	NO	YES	NO

© 2024 Lee DY et al. *JAMA Network Open*

© 2024 Lee DY et al. *JAMA Network Open*

eAppendix 2. The list of code for study variables used in definition

Variables	Vocabulary	OMOP Vocabulary Codes
Attention-deficit/hyperactivity disorder	SNOMED	45765796 (Attention deficit hyperactivity disorder, inattentive presentation),<br>44784525 (Attention deficit hyperactivity disorder, predominantly hyperactive impulsive type in remission),<br>44782517 (Attention deficit hyperactivity disorder, predominantly inattentive type in remission),<br>4253962 (Attention deficit hyperactivity disorder, predominantly hyperactive impulsive type),<br>4149904 (Attention deficit hyperactivity disorder, combined type),<br>4149353 (Attention deficit hyperactivity disorder, predominantly inattentive type),<br>4049391 (Undifferentiated attention deficit disorder),<br>4047120 (Disorders of attention and motor control),<br>4041692 (Deficits in attention motor control and perception),<br>440086 (Child attention deficit disorder),<br>438409 (Attention deficit hyperactivity disorder),<br>438132 (Hyperkinetic conduct disorder),<br>437261 (Hyperkinesis with developmental delay)
Anxiety disorder	SNOMED	442077 (Anxiety disorder),<br>37109206 (Anxiety disorder caused by drug),<br>4199892 (Anxiety disorder due to a general medical condition),<br>434613 (Generalized anxiety disorder),<br>4338031 (Mixed anxiety and depressive disorder),<br>381537 (Organic anxiety disorder),<br>436074 (Panic disorder),<br>4304010 (Phobic disorder)
Mania	SNOMED	4333677 (Mania),<br>35610112 (Mania with psychotic features),<br>440078 (Bipolar affective disorder, current episode manic),<br>439256 (Bipolar affective disorder, currently manic, severe, with psychosis),<br>4150985 (Bipolar I disorder, most recent episode hypomanic),<br>4153292 (Schizoaffective disorder, manic type),<br>35610112 (Mania with psychotic features)
Sleep disorder	SNOMED	4102985 (Nonorganic insomnia),<br>439708 (Disorders of initiating and maintaining sleep),<br>435524 (Sleep disorder),<br>442588 (Obstructive sleep apnea syndrome),<br>374905 (Non-organic sleep disorder),<br>313459 (Sleep apnea),<br>4047912 (Hypersomnia of non-organic origin),<br>437854 (Cataplexy and narcolepsy),<br>4232324 (Sleep terror disorder),<br>4108537 (Disorders of excessive somnolence),<br>377535 (Sleep walking disorder),<br>377266 (Non-organic disorder of the sleep-wake schedule),<br>439794 (Central sleep apnea syndrome),<br>435786 (Disorder of sleep-wake cycle),<br>4262580 (Primary sleep apnea of newborn)
Tic disorder	SNOMED	37312584 (Transient motor tic),<br>37119137 (Infection causing tic),<br>37110326 (Tic due to and following infection),<br>36716895 (Primary tic disorder),<br>36716791 (Tic due to developmental disorder),<br>36716790 (Secondary tic disorder),<br>4299573 (Habit tic affecting hair),<br>4299571 (Habit tic),<br>4296290 (Atypical tic disorder),<br>4291439 (Habit tic affecting skin),<br>4250625 (Chronic vocal tic disorder),<br>4226426 (Transient tic disorder, single episode),<br>4210636 (Transient tic disorder),<br>4196373 (Recurrent transient tic disorder),<br>4048017 (Cluster tic syndrome),<br>4047760 (Facial tic disorder),<br>4046101 (Gestural tic disorder),<br>4044062 (Motor tic disorder),<br>4044061 (Drug-induced tic),<br>4043396 (Vocal tic disorder),<br>4034990 (Dysphonia of Gilles de la Tourette's syndrome),<br>762975 (Acute tic disorder),<br>381839 (Tic disorder),<br>379782 (Gilles de la Tourette's syndrome),

		377265 (Transient childhood tic),<br>375221 (Chronic motor tic disorder)
Tremor	SNOMED	46273634 (Tremor due to orthostatic hypotension),<br>42596632 (Head tremor),<br>42596631 (Tremor exaggerated by excitement),<br>42535533 (Rubral tremor),<br>40483671 (Psychogenic tremor),<br>40483375 (Chronic tremor),<br>37396185 (Primary orthostatic tremor),<br>37311986 (Dissociative neurological symptom disorder co-occurrent with tremor),<br>37110509 (Tremor due to central nervous system disease),<br>37110508 (Enhanced physiological tremor),<br>36716789 (Tremor due to drug withdrawal),<br>36716788 (Tremor due to substance abuse),<br>36716787 (Tremor due to metabolic disorder),<br>4334342 (Orthostatic tremor),<br>4321085 (Mercurial tremor),<br>4302311 (Coarse tremor),<br>4284539 (Tremor opiophagorum),<br>4279391 (Arsenical tremor),<br>4278463 (Intermittent tremor),<br>4251034 (Metallic tremor),<br>4232782 (Post-hemiplegic tremor),<br>4222566 (Passive tremor),<br>4222119 (Continuous tremor),<br>4215954 (Benedikt's syndrome),<br>4215654 (O/E - tremor of tongue),<br>4213644 (O/E - tremor outstretched hands),<br>4209594 (Static tremor),<br>4204820 (Parkinsonian tremor),<br>4204051 (Saturnine tremor),<br>4201257 (Thyrotoxic tremor),<br>4177049 (Fine tremor),<br>4173606 (Tremor of palate),<br>4171685 (Darkness tremor),<br>4163369 (Persistent tremor),<br>4148954 (Intention tremor),<br>4145813 (Trembles),<br>4141836 (Dystonic tremor),<br>4138992 (Asterixis),<br>4138028 (Physiological tremor),<br>4100050 (Massive tremor),<br>4096245 (Resting tremor),<br>4082169 (Halothane shakes),<br>4077310 (Toxic tremor),<br>4063335 (O/E - Parkinsonian tremor),<br>4044064 (Isolated vocal tremor),<br>4044063 (Isolated facial tremor),<br>4043397 (Isolated head tremor),<br>4039118 (O/E - intention tremor),<br>4039117 (O/E - fine tremor),<br>4038910 (O/E - coarse tremor - flapping),<br>4008895 (Senile tremor),<br>4008894 (Pill rolling),<br>3183493 (Excessive physiologic tremor),<br>3178103 (Tremor of hands and face),<br>443782 (Tremor)
Anemia	SNOMED	439777 (Anemia)
Arrhythmia	SNOMED	4154290 (Paroxysmal atrial fibrillation),<br>4068155 (Atrial arrhythmia),<br>4275423 (Supraventricular tachycardia),<br>44784217 (Cardiac arrhythmia),<br>320744 (Complete atrioventricular block),<br>4120088 (Cardiac arrest with successful resuscitation),<br>4091901 (Aberrant premature complexes),<br>4141360 (Chronic atrial fibrillation),<br>321042 (Cardiac arrest),<br>4261842 (Sick sinus syndrome),<br>4057008 (Accelerated atrioventricular conduction),<br>4089462 (Ventricular premature complex),<br>4232697 (Persistent atrial fibrillation),<br>444070 (Tachycardia),<br>313792 (Paroxysmal tachycardia),

		314059 (Right bundle branch block),<br>4185572 (Ventricular arrhythmia),<br>318448 (Second degree atrioventricular block),<br>316998 (Left bundle branch block),<br>316135 (Atrioventricular block),<br>4115173 (Atrial premature complex),<br>316999 (Conduction disorder of the heart),<br>320425 (Heart block),<br>437894 (Ventricular fibrillation),<br>314379 (First degree atrioventricular block),<br>313791 (Bundle branch block),<br>4173170 (Neonatal dysrhythmia),<br>36714994 (Typical atrial flutter),<br>4088351 (Junctional premature complex),<br>4111552 (Re-entry ventricular arrhythmia),<br>4166844 (Intraventricular conduction defect),<br>36712986 (Atypical atrial flutter),<br>4295336 (Left anterior fascicular block),<br>4250169 (Bifascicular block),<br>321315 (Trifascicular block),<br>315069 (Congenital heart block)
Abdominal pain	SNOMED	200219 (Abdominal pain),<br>4128083 (Nonspecific abdominal pain),<br>4306292 (Upper abdominal pain),<br>4137754 (Secondary dysmenorrhea),<br>194696 (Dysmenorrhea),<br>197381 (Epigastric pain),<br>4182562 (Lower abdominal pain),<br>40483351 (Tenderness of periumbilical region),<br>197988 (Generalized abdominal pain),<br>4280657 (Primary dysmenorrhea),<br>4322528 (Abdominal colic),<br>198263 (Right upper quadrant pain),<br>193322 (Right lower quadrant pain),<br>201626 (Mittelschmerz),<br>195083 (Left lower quadrant pain),<br>201690 (Renal colic),<br>194175 (Left upper quadrant pain),<br>197981 (Abdominal tenderness),<br>443629 (Generalized abdominal tenderness),<br>194491 (Tenderness of epigastrium),<br>40490986 (Tenderness of right lower quadrant of abdomen),<br>40481920 (Periumbilical pain),<br>194755 (Abdominal tenderness of left lower quadrant),<br>40488954 (Tenderness of right upper quadrant of abdomen),<br>4115549 (Rebound tenderness of right iliac fossa),<br>4149024 (Rebound tenderness),<br>4285744 (Painful bladder spasm)
Constipation	SNOMED	75860 (Constipation),<br>79061 (Slow transit constipation),<br>201905 (Constipation by outlet obstruction)
Dizziness	SNOMED	4223938 (Dizziness)
Headache	SNOMED	46270383 (Headache caused by drug),<br>46270382 (Intractable headache caused by drug),<br>45766090 (Headache due to reversible cerebral vasoconstriction syndrome),<br>44790934 (Increased frequency of headaches),<br>44783586 (Chronic post-concussion headache),<br>44782806 (Medication overuse headache),<br>44782681 (Acute posttraumatic headache),<br>42539583 (Acute headache),<br>42538612 (Persistent headache due to and following injury of head),<br>42538611 (Acute headache due to traumatic injury of head),<br>40483832 (Orthostatic headache),<br>40482953 (Migraine variant with headache),<br>37110728 (Short-lasting unilateral neuralgiform headache attacks with conjunctival injection and tearing syndrome),<br>37016725 (Frequent headache),<br>37016721 (Intermittent headache),<br>36716800 (Headache due to injury of head and neck),<br>4315023 (Ocular headache),<br>4309592 (Aural headache),<br>4271776 (Headache character - finding),<br>4212985 (Temporal headache),

		4195951 (Nasal headache),<br>4193674 (Benign coital headache),<br>4183441 (Drug withdrawal headache),<br>4172302 (Sinus headache),<br>4170790 (O/E -frontal sinus pain),<br>4170789 (On examination - maxillary sinus pain),<br>4158490 (C/O - a headache),<br>4149969 (Cervicogenic headache),<br>4147325 (Frontal headache),<br>4140381 (Occipital headache),<br>4115407 (Frontal sinus pain),<br>4103477 (Maxillary sinus pain),<br>4061404 (Headache caused by oral contraceptive pill),<br>4044237 (Headache associated with substance abuse or withdrawal),<br>4038026 (Shooting headache),<br>4038025 (Aching headache),<br>4037891 (Bilateral headache),<br>4037300 (Parietal headache),<br>4036953 (Morning headache),<br>4036625 (Heavy head),<br>4036624 (Throbbing headache),<br>4036622 (Generalized headache),<br>4030433 (Headache due to intracranial disease),<br>4028205 (Postseizure headache),<br>4012515 (Viral headache),<br>4012241 (Analgesic overuse headache),<br>4012240 (Postpartum headache),<br>4012239 (Headache due to external compression of head),<br>4011768 (Headache due to cold exposure),<br>4011530 (Dental headache),<br>3179785 (Intractable headache),<br>3170444 (Occipital pain),<br>763746 (Postural headache),<br>762083 (Intractable chronic headache following trauma),<br>378253 (Headache),<br>377546 (Chronic post-traumatic headache),<br>376104 (Posttraumatic headache),<br>374342 (Headache associated with sexual activity),<br>373463 (Cough headache syndrome),<br>372313 (Benign exertional headache)
Hypertension	SNOMED	312648 (Benign essential hypertension),<br>317898 (Malignant essential hypertension),<br>320128 (Essential hypertension),<br>4034031 (Benign essential hypertension complicating AND/OR reason for care during pregnancy),<br>4058987 (High-renin essential hypertension),<br>4083723 (Essential hypertension complicating AND/OR reason for care during childbirth),<br>4148205 (Benign essential hypertension complicating AND/OR reason for care during puerperium),<br>4159755 (Labile essential hypertension),<br>4180283 (Systolic essential hypertension),<br>4215640 (Benign essential hypertension complicating AND/OR reason for care during childbirth),<br>4217486 (Essential hypertension in obstetric context),<br>4263067 (Low-renin essential hypertension),<br>4269358 (Benign essential hypertension in obstetric context),<br>4302591 (Essential hypertension complicating AND/OR reason for care during pregnancy),<br>4321603 (Essential hypertension complicating AND/OR reason for care during puerperium),<br>45757787 (Postpartum pre-existing essential hypertension)
Hyperlipidemia	SNOMED	432867 (Hyperlipidemia)
Nausea vomiting	SNOMED	27674 (Nausea and vomiting),<br>26727 (Hematemesis),<br>31967 (Nausea),<br>441408 (Vomiting),<br>436166 (Mild hyperemesis gravidarum),<br>4169915 (Vomiting in newborn),<br>436485 (Hyperemesis gravidarum with metabolic disturbance),<br>440785 (Vomiting of pregnancy),<br>4071070 (Neonatal hematemesis),<br>4102984 (Vomiting associated with other psychological disturbances),

© 2024 Lee DY et al. *JAMA Network Open*

		22666 (Vomiting after gastrointestinal tract surgery),<br>4312477 (Projectile vomiting),<br>30284 (Motion sickness),<br>35625971 (Vomiting during third trimester of pregnancy)
Seizure	SNOMED	377091 (Seizure),<br>380378 (Epilepsy),<br>4310999 (Epilepsy, not refractory),<br>4101747 (Localization-related(focal)(partial)idiopathic epilepsy and epileptic syndromes with seizures of localized onset),<br>444413 (Febrile convulsion),<br>4044082 (Localization-related symptomatic epilepsy),<br>4332304 (Status epilepticus),<br>4274575 (Idiopathic generalized epilepsy),<br>4236312 (Complex partial epileptic seizure),<br>43530626 (Simple partial seizure),<br>762976 (Idiopathic generalized epilepsy, non-refractory),<br>4055361 (Generalized epilepsy),<br>40483317 (Refractory epilepsy),<br>380533 (Convulsions in the newborn),<br>46271795 (Refractory idiopathic generalized epilepsy),<br>4043559 (Complex partial status epilepticus),<br>4309257 (Refractory localization-related epilepsy),<br>4047897 (Epilepsy with grand mal seizures on awakening),<br>373748 (Grand mal status),<br>40483585 (Epilepsy characterized by intractable complex partial seizures),<br>4044221 (Localization-related symptomatic epilepsy with specific precipitant),<br>374023 (Epilepsia partialis continua),<br>762832 (Epilepsia partialis continua, non-refractory),<br>762957 (Tonic-clonic seizure, non-refractory),<br>4311977 (Refractory epilepsia partialis continua),<br>137613 (Eclampsia in pregnancy),<br>443700 (Eclampsia),<br>4046219 (Acquired epileptic aphasia),<br>4179936 (Childhood absence epilepsy),<br>762734 (Childhood absence epilepsy, non-refractory),<br>4116344 (Eclampsia in puerperium),<br>762733 (Childhood absence epilepsy, refractory),<br>765514 (Tonic-clonic seizure, refractory),<br>4034096 (Eclampsia in labor)
Traumatic injury	SNOMED	4094343 (Dislocations/sprains/strains),<br>444187 (Open wound),<br>4058494 (Superficial injuries involving multiple body regions),<br>435082 (Closed fracture of nasal bones),<br>4001336 (Concussion injury of brain),<br>4243161 (Open wound of head),<br>4234112 (Brain injury without open intracranial wound),<br>4014781 (Closed traumatic subdural hemorrhage),<br>4086195 (Superficial injury of head),<br>4096616 (Diffuse brain injury),<br>40480160 (Pathological fracture due to osteoporosis),<br>4096315 (Injury of cervical spine),<br>4335872 (Injury of globe of eye),<br>4307254 (Closed fracture),<br>4264073 (Fracture of tooth),<br>4053584 (Superficial injury),<br>4128914 (Fracture of multiple ribs),<br>4129394 (Fracture of lumbar spine),<br>4011508 (Fracture of skull and facial bones),<br>45757291 (Closed fracture of orbital floor),<br>198892 (Internal injury of abdominal organs without open wound into cavity),<br>437409 (Intracranial injury),<br>435959 (Subarachnoid hemorrhage following injury without open intracranial wound),<br>4052826 (Open wound in mouth),<br>4167105 (Superficial injury of scalp),<br>4095852 (Injury of lumbar spine),<br>4013613 (Fracture of lumbar spine and/or pelvis),<br>252477 (Extradural hemorrhage following injury without open intracranial wound),<br>258895 (Injury of lung without open wound into thorax),<br>4053828 (Fracture of thoracic spine),<br>4237458 (Fracture of clavicle),<br>4138294 (Fracture of mandible),<br>435093 (Closed fracture of femur),<br>4096313 (Multiple injuries),

		4082029 (Fracture of base of skull),<br>4134322 (Fracture of distal end of radius),<br>4002661 (Superficial injury of mouth),<br>77162 (Injury of ankle),<br>437993 (Closed fracture of cervical spine),<br>4226266 (Cervical spinal cord injury),<br>4320628 (Fracture of shaft of tibia),<br>4054063 (Open wound of finger),<br>380859 (Fracture of mandible, closed),<br>4168152 (Fracture of vault of skull),<br>438887 (Closed fracture of shaft of femur),<br>78619 (Contusion of knee),<br>4185758 (Fracture of lower leg),<br>73075 (Contusion of upper limb),<br>435376 (Multiple fractures involving skull and facial bones),<br>195977 (Contusion of trunk),<br>4051004 (Open wound of scalp),<br>4216185 (Open wound of upper limb),<br>381444 (Contusion of eye),<br>4138299 (Fracture of distal end of humerus),<br>4011509 (Fracture of malar and maxillary bones),<br>435940 (Closed fracture of shaft of humerus),<br>4167229 (Superficial injury of upper limb),<br>4194894 (Muscle and tendon injury),<br>433856 (Fracture of neck of femur),<br>4070301 (Fracture of upper end of tibia),<br>40485073 (Injury of cruciate ligament of knee),<br>4136718 (Fracture of proximal end of ulna),<br>441712 (Open wound of ocular adnexa),<br>80232 (Closed fracture of calcaneus),<br>4067768 (Postmenopausal osteoporosis with pathological fracture),<br>81696 (Closed fracture of acetabulum),<br>4133194 (Fracture of distal end of tibia),<br>440228 (Closed fracture of lower end of humerus),<br>81175 (Contusion of chest),<br>440238 (Closed fracture of metatarsal bone),<br>375415 (Injury of head),<br>4015503 (Pertrochanteric fracture),<br>4013604 (Closed fracture lumbar vertebra),<br>442013 (Burn),<br>4057580 (Fracture of phalanx of finger),<br>73925 (Burn of upper limb),<br>4013160 (Closed fracture sacrum),<br>436539 (Closed fracture of patella),<br>441428 (Closed fracture of lateral malleolus),<br>201441 (Traumatic hemothorax without open wound into thorax),<br>253896 (Traumatic pneumothorax without open wound into thorax),<br>4069306 (Idiopathic osteoporosis with pathological fracture),<br>200562 (Traumatic pneumohemothorax without open wound into thorax),<br>441974 (Closed fracture of forearm),<br>4053272 (Injury of spleen),<br>436252 (Closed fracture of shaft of tibia),<br>4319889 (Fracture of scapula),<br>76240 (Closed fracture pubis),<br>4133610 (Fracture of upper end of humerus),<br>4235863 (Spinal cord injury),<br>81426 (Closed fracture of clavicle),<br>436832 (Closed fracture of sternum),<br>373035 (Closed fracture of malar AND/OR maxillary bones),<br>4015350 (Fracture at wrist and/or hand level),<br>4101989 (Closed fracture of shaft of bone of forearm),<br>434500 (Closed fracture of neck of femur),<br>4054908 (Closed injury of kidney),<br>4142905 (Fracture of rib),<br>439162 (Closed fracture of medial malleolus),<br>4134336 (Fracture of tarsal bone),<br>4135749 (Fracture of distal end of femur),<br>444132 (Injury of knee),<br>76611 (Injury of shoulder region),<br>436209 (Fracture of fibula),<br>4168796 (Muscle strain),<br>197751 (Burn of lower limb),<br>4115175 (Pathological fractures - multiple),<br>4219836 (Contusion of ocular adnexa and periocular tissues),

		375404 (Ocular laceration with prolapse AND/OR exposure of intraocular tissue),<br>4166909 (Superficial injury of lower limb),<br>4334734 (Penetrating wound of orbit),<br>4152163 (Injury of eye region),<br>81454 (Injury of finger),<br>4177069 (Closed fracture of base of skull),<br>42536845 (Injury of Achilles tendon),<br>373648 (Superficial injury of eyelid AND/OR periocular area),<br>4016540 (Focal brain injury),<br>4015352 (Fracture of metacarpal bone),<br>78273 (Closed fracture of multiple ribs),<br>74188 (Closed fracture of rib),<br>24818 (Injury of neck),<br>80552 (Closed fracture of scaphoid bone of wrist),<br>4211657 (Closed fracture of lower leg),<br>4049930 (Closed injury of urethra),<br>78605 (Contusion of elbow),<br>4067970 (Open fracture of phalanx of finger),<br>81141 (Closed fracture of ilium),<br>4053604 (Open wound of lower leg),<br>73574 (Nonunion of fracture),<br>437116 (Closed fracture of distal end of radius),<br>4106355 (Superficial injury of lower leg),<br>4013596 (Closed fracture thoracic vertebra),<br>194526 (Injury of trunk),<br>372479 (Laceration of eye),<br>136580 (Dehiscence of surgical wound),<br>4153877 (Post-traumatic wound infection),<br>4293479 (Burn of head AND/OR neck),<br>4130851 (Injury of upper extremity),<br>4136715 (Fracture of proximal end of radius),<br>73649 (Contusion of finger),<br>440538 (Closed fracture of lower end of radius AND ulna),<br>374221 (Injury of brachial plexus),<br>77734 (Contusion of lower leg),<br>435666 (Closed fracture of upper end of tibia),<br>434494 (Closed fracture of upper end of humerus),<br>434502 (Closed fracture of phalanx of foot),<br>4167909 (Injury of nail),<br>441191 (Poisoning due to arthropod venom),<br>80573 (Contusion of ankle),<br>4012455 (Closed fracture distal tibia),<br>73090 (Contusion of foot),<br>4008356 (Multiple fractures of lumbar spine and/or pelvis),<br>4053599 (Open wound of forearm),<br>4054067 (Open wound of foot),<br>196569 (Contusion of abdominal wall),<br>4319151 (Traumatic amputation of finger),<br>4136717 (Fracture of shaft of radius),<br>437689 (Multiple closed fractures of cervical vertebrae),<br>4009610 (Closed fracture proximal femur, subtrochanteric),<br>40485074 (Injury of collateral ligament of knee),<br>74777 (Closed fracture of talus),<br>4145094 (Traumatic tympanic membrane perforation),<br>4183876 (Old rupture of cartilage AND/OR meniscus of knee),<br>197136 (Injury of small intestine without open wound into abdominal cavity),<br>444190 (Injury of upper arm),<br>4209549 (Closed multiple fractures of thoracic spine),<br>4129407 (Open wound of knee),<br>436251 (Closed fracture of proximal end of ulna),<br>444129 (Injury of wrist),<br>4001458 (Fatigue fracture of vertebra),<br>433618 (Open fracture of patella),<br>195401 (Contusion of hip),<br>441980 (Closed fracture of foot),<br>78609 (Crushing injury of upper limb),<br>201996 (Internal injury of abdominal organs with open wound into cavity),<br>195124 (Injury of celiac AND/OR mesenteric arteries),<br>316457 (Mallory-Weiss syndrome),<br>4052818 (Traumatic amputation of ear),<br>4218884 (Fracture of carpal bone),<br>437693 (Open fracture of shaft of tibia),<br>133384 (Burn any degree involving less than 10 percent of body surface),<br>37116489 (Injury of intrathoracic organ),

© 2024 Lee DY et al. *JAMA Network Open*

		74809 (Crushing injury of hand),<br>4034214 (Open wound of ear),<br>442639 (Injury of colon without open wound into abdominal cavity),<br>436445 (Abrasion of tooth),<br>4027460 (Closed pertrochanteric fracture),<br>4062251 (Injury of nerves and spinal cord at neck level),<br>436839 (Closed fracture of metacarpal bone),<br>4134325 (Fracture of shaft of ulna),<br>4095851 (Injury of thoracic spine),<br>4206872 (Closed fracture of great toe),<br>4170635 (Superficial injury of neck),<br>4106172 (Traumatic injury of common peroneal nerve),<br>441973 (Closed fracture of proximal end of radius),<br>44784606 (Burn of cornea and conjunctival sac),<br>73065 (Contusion of genital organ),<br>197163 (Burn of trunk),<br>4047852 (Cephalhematoma due to birth trauma),<br>36717471 (Injury of thoracic spinal cord),<br>36716566 (Penetrating wound of eyeball without foreign body),<br>4108637 (Superficial injury of nose),<br>4052200 (Portal system vein injury),<br>438590 (Brain injury with open intracranial wound),<br>36717576 (Injury of lumbar spinal cord),<br>4019728 (Closed injury of pancreas),<br>444189 (Injury of elbow),<br>4147013 (Closed injury of bladder),<br>433329 (Closed fracture of second cervical vertebra),<br>433064 (Superficial injury of shoulder and upper arm),<br>4062388 (Injury of muscle and tendon at forearm level),<br>37116361 (Accidental wound during procedure),<br>194229 (Crushing injury of lower limb),<br>381732 (Nerve injury),<br>4108638 (Superficial injury of forearm),<br>320563 (Injury of heart without open wound into thorax),<br>4094683 (Chest injury),<br>195682 (Pelvic organ injury without open wound into abdominal cavity),<br>140273 (Contusion of forearm),<br>444130 (Injury of foot),<br>79174 (Open fracture of calcaneus),<br>46273347 (Eyeball wound due to penetrating foreign body),<br>440856 (Open fracture of shaft of femur),<br>4320024 (Traumatic amputation of arm),<br>40492392 (Closed fracture of fibula),<br>133637 (Partial thickness burn of lower limb),<br>193666 (Injury of hip region),<br>4055709 (Superficial injury of trunk),<br>4106678 (Superficial injury of ear region),<br>382014 (Injury of facial nerve),<br>436278 (Contusion of thigh),<br>434140 (Pathological fracture - pelvis and/or thigh),<br>4154163 (Jaw injury),<br>4107362 (Burn of ankle and foot),<br>4296205 (Partial thickness burn),<br>4053602 (Open wound of thigh),<br>4309483 (Dislocations, sprains and strains involving multiple body regions),<br>4166901 (Fracture of phalanx of thumb),<br>35624169 (Closed fracture of coccyx),<br>441177 (Partial thickness burn of upper limb),<br>440548 (Closed fracture of femur, distal end),<br>4011811 (Injury to multiple structures of knee),<br>4059257 (Injury of muscle and tendon at lower leg level),<br>4057044 (Injury of muscle and tendon at neck level),<br>436826 (Closed fracture of shaft of radius),<br>4057177 (Injury of muscle and tendon at ankle and foot level),<br>79938 (Closed fracture of scapula),<br>434770 (Closed fracture of bones of trunk),<br>36715774 (Injury of ulnar nerve at forearm level),<br>443268 (Open wound of abdominal wall),<br>44783028 (Injury of shoulder and upper arm),<br>4096314 (Thumb injury),<br>4264281 (Open fracture),<br>73366 (Crushing injury of lower leg),<br>379157 (Open fracture of orbital floor),<br>4059565 (Chemical burn of eyelid region),

		434497 (Open fracture of shaft of humerus),<br>136853 (Partial thickness burn of trunk),<br>4056714 (Injury of flexor muscle and tendon of thumb at forearm level),<br>444095 (Injury of ureter without open wound into abdominal cavity),<br>40485984 (Traumatic injury of digital nerve of hand),<br>436256 (Open fracture of metatarsal bone),<br>195589 (Traumatic urethral stricture),<br>433917 (Burns of multiple sites),<br>434176 (Open fracture of nasal bones),<br>4130852 (Injury of lower extremity),<br>4056717 (Injury of muscle and tendon at hip and thigh level),<br>36716572 (Focal traumatic hematoma of cerebellum),<br>4148331 (Closed flail chest),<br>432749 (Open fracture of medial malleolus),<br>4170742 (Closed fracture of vertebral column),<br>4022202 (Open wound of trunk),<br>441979 (Open fracture of upper end of tibia),<br>4318111 (Traumatic amputation of lower extremity),<br>4054058 (Traumatic dislocation of joint of wrist),<br>4164345 (Superficial injury of chest),<br>4088051 (Closed fracture of phalanx of finger),<br>4053837 (Open wound of toe),<br>4013612 (Fracture of first cervical vertebra),<br>4208028 (Injury of median nerve distal to forearm),<br>4015971 (Multiple fractures of forearm),<br>4097962 (Open wound of lower limb),<br>37116485 (Open wound of thorax),<br>4102481 (Nerve root and plexus compressions in intervertebral disc disorders),<br>74816 (Contusion of toe),<br>4051005 (Open wound of nose),<br>4001487 (Avulsion of scalp),<br>36674267 (Partial thickness burn of head and/or neck),<br>4129404 (Open wound of upper arm),<br>192753 (Injury of small intestine with open wound into abdominal cavity),<br>75413 (Burn of wrist),<br>4129408 (Open wound of ankle),<br>198019 (Iliac blood vessel injury),<br>437400 (Closed fracture of shaft of ulna),<br>4239520 (Open wound of external genital organs),<br>75426 (Burn of hand),<br>36716584 (Traumatic amputation of part of head),<br>36715776 (Injury of radial nerve at forearm level),<br>4009757 (Multiple fractures of foot),<br>4053597 (Open wound of neck),<br>4053600 (Open wound of elbow),<br>37119157 (Injury of tibial nerve at lower leg level),<br>4085340 (Radiation injury),<br>437998 (Open fracture of lateral malleolus),<br>4067766 (Fracture of bone in neoplastic disease),<br>139200 (Crushing injury of forearm),<br>4308851 (Dislocations, sprains and strains involving thorax with lower back and pelvis),<br>4057183 (Multiple open wounds of lower leg),<br>42537893 (Traumatic injury of vertebral region of back),<br>440851 (Open fracture of forearm),<br>73571 (Pathological fracture),<br>37116602 (Open wound of abdomen),<br>255711 (Optic nerve and pathway injury),<br>432747 (Open fracture of lower end of radius AND ulna),<br>4209550 (Open fracture of great toe),<br>44783029 (Injury of hip and thigh),<br>313062 (Injury of thoracic aorta),<br>4233525 (Crushing injury of face),<br>198645 (Full thickness burn of lower limb),<br>440231 (Closed fracture of multiple sites of metacarpus),<br>36715775 (Injury of median nerve at forearm level),<br>434824 (Injury of cranial nerve),<br>438583 (Open fracture of femur),<br>4017105 (Subdural hemorrhage following open wound of head),<br>4012456 (Open fracture distal tibia),<br>435684 (Injury of popliteal artery),<br>435960 (Extradural hemorrhage following injury with open intracranial wound),<br>45772707 (Closed fracture of phalanx of thumb),<br>315116 (Injury of carotid artery),

		4056713 (Injury of muscle and tendon of abdomen, lower back and pelvis),<br>4191822 (Multiple open wounds of wrist and hand),<br>439215 (Traumatic injury due to event),<br>4136719 (Fracture of shaft of radius and/or ulna),<br>192979 (Obstetric high vaginal laceration),<br>4010385 (Fracture of first metacarpal bone),<br>195958 (Traumatic pneumohemothorax with open wound into thorax),<br>443982 (Traumatic amputation of thumb),<br>4051764 (Contusion, throat),<br>4012753 (Tear of articular cartilage of knee, current),<br>194209 (Digital blood vessel injury),<br>4019341 (Injury of heart with hemopericardium),<br>198623 (Epidermal burn of trunk),<br>4043672 (Injury of nerve of upper extremity),<br>37118664 (Injury of femoral artery),<br>4134932 (Injury of male genital organ),<br>435701 (Injury of trigeminal nerve),<br>433894 (Open fracture of phalanx of foot),<br>4013449 (Traumatic rupture of symphysis pubis),<br>4048135 (Fracture of skull due to birth trauma),<br>36716599 (Injury of radial nerve at upper arm level),<br>4138484 (Chemical burn),<br>260245 (Traumatic hemothorax with open wound into thorax),<br>4062133 (Infection of obstetric surgical wound),<br>197344 (Cesarean wound disruption),<br>4285285 (Closed fracture of multiple sites of phalanges of hand),<br>42536812 (Traumatic rupture of ligament of wrist),<br>4151986 (Injury of patella),<br>4116717 (Injury of artery of lower limb),<br>438024 (Injury of tibial blood vessel),<br>40441585 (Stress fracture),<br>4096479 (Traumatic amputation),<br>380265 (Injury to oculomotor nerve),<br>4054878 (Injury of ulnar nerve at upper arm level),<br>4054910 (Open injury of bladder),<br>37117753 (Traumatic amputation of forearm),<br>4015356 (Closed fracture of thumb metacarpal),<br>201164 (Injury of blood vessels of upper extremity),<br>193344 (Injury of lung with open wound into thorax),<br>36674268 (Epidermal burn of head and/or neck),<br>138612 (Injury of cervical nerve roots),<br>378265 (Open fracture of mandible),<br>198010 (Injury of stomach without open wound into abdominal cavity),<br>4054857 (Injury of ulnar nerve at wrist and hand level),<br>317713 (Injury of blood vessels of thorax),<br>4044402 (Injury of cauda equina),<br>4058370 (Contusion of toe(s) with damage to nail),<br>77131 (Open fracture of talus),<br>438600 (Injury of blood vessels of abdomen AND/OR pelvis),<br>4015743 (Multiple fractures of lower leg),<br>437428 (Crushing injury),<br>43021072 (Open fracture of bone of wrist and/or hand),<br>192763 (Injury of blood vessel),<br>201160 (Injury of colon with open wound into abdominal cavity),<br>444131 (Injury of lower leg),<br>142032 (Partial thickness burn of lower leg),<br>4049931 (Open injury of urethra),<br>193079 (Injury of nerve of trunk),<br>4056428 (Open wound of toe(s) with damage to nail),<br>4050964 (Traumatic injury of deep peroneal nerve),<br>4005907 (Injury of brachial artery),<br>78621 (Crushing injury of knee),<br>36717481 (Injury of nerve at forearm level),<br>4053598 (Open wound of shoulder region),<br>78009 (Traumatic pneumothorax with open wound into thorax),<br>374226 (Burn of eye region),<br>40305987 (Delayed union of fracture),<br>4151985 (Lower back injury),<br>4112302 (Strain of muscle of lower limb),<br>433916 (Sympathetic nerve injury),<br>40491339 (Closed fracture of tarsal bone),<br>4067765 (Drug-induced osteoporosis with pathological fracture),<br>4059258 (Injury of muscle(s) and tendon(s) of peroneal muscle group at lower leg level),

© 2024 Lee DY et al. *JAMA Network Open*

		4050542 (Renal blood vessel injury),<br>133367 (Partial thickness burn of wrist),<br>440511 (Pathological fracture - forearm),<br>435094 (Open fracture of femur, distal end),<br>4186548 (Open fracture of lower leg),<br>194439 (Obstetric perineal wound disruption),<br>4057178 (Injury of muscle and tendon of long extensor muscle of toe at ankle and foot level),<br>435101 (Open fracture of foot),<br>75091 (Closed fracture of carpal bone),<br>441571 (Retained old foreign body following penetrating wound of orbit),<br>4308850 (Dislocations, sprains and strains involving head with neck),<br>4134940 (Injury to blood vessel of neck),<br>4115178 (Fracture malunion - shoulder),<br>4109087 (Strain of muscle of upper limb),<br>4050547 (Injury of radial artery at wrist and hand level),<br>4145607 (Injury of radial artery),<br>382034 (Injury of sciatic nerve),<br>75709 (Full thickness burn of upper limb),<br>4002499 (Decapitation),<br>139970 (Partial thickness burn of thigh),<br>37111384 (Injury of intrinsic muscle of finger),<br>4062391 (Injury of muscle and tendon of long flexor muscle of toe at ankle and foot level),<br>4208505 (Traumatic cerebral edema with open intracranial wound),<br>4052209 (Injury of ulnar artery at wrist and hand level),<br>4003483 (Osteoporosis of disuse with pathological fracture),<br>36674774 (Partial thickness burn of skin of finger),<br>381952 (Injury to brachial plexus as birth trauma),<br>440546 (Open fracture of distal end of radius),<br>443248 (Open fracture of cervical spine),<br>75634 (Fracture malunion),<br>75121 (Burn of lower leg),<br>196307 (Injury of lumbosacral plexus),<br>193830 (Third degree perineal laceration),<br>437121 (Open fracture of lower end of humerus),<br>375716 (Traumatic injury of abducens nerve),<br>4059256 (Injury of multiple muscles and tendons at hip and thigh level),<br>80592 (Crushing injury of ankle),<br>141456 (Chilblains),<br>44784105 (Injury of free lower limb),<br>75092 (Open fracture of multiple sites of phalanges of hand),<br>4289013 (Multiple closed fractures of lower end of femur),<br>197417 (Injury of ureter with open wound into abdominal cavity),<br>4218146 (Closed fracture dislocation of joint of shoulder girdle),<br>134222 (Injury of forearm),<br>74533 (Crushing injury of toe),<br>4149044 (Injury of ulnar artery),<br>4210438 (Closed multiple fractures of clavicle and/or scapula and/or humerus),<br>4319152 (Traumatic amputation of toe),<br>4133009 (Injury of female genital system),<br>200564 (Injury of stomach with open wound into abdominal cavity),<br>4118017 (Pathological fracture - hand),<br>46271082 (Injury of nerve at lower leg level),<br>4107672 (Eyelid burn),<br>437149 (Injury of femoral vein),<br>42538212 (Full thickness burn of head and neck),<br>433076 (Crushing injury of thigh),<br>436206 (Pathological fracture - upper arm),<br>4050679 (Injury of blood vessels at lower leg level),<br>4050089 (Open wound of front wall of thorax),<br>4054859 (Injury of radial nerve at wrist and hand level),<br>433047 (Open fracture of proximal end of ulna),<br>4057176 (Injury of extensor or abductor muscles and tendons of thumb at forearm level),<br>4309929 (Fractures involving multiple body regions),<br>4054909 (Open injury of kidney),<br>4151204 (Injury of toe),<br>434224 (Injury of popliteal vein),<br>141474 (Partial thickness burn of hand),<br>4059259 (Injury of multiple muscles and tendons at lower leg level),<br>135699 (Burn any degree involving 10-19 percent of body surface),<br>4296204 (Epidermal burn of skin),<br>79962 (Crushing injury of elbow),

© 2024 Lee DY et al. *JAMA Network Open*

		4096625 (Injury of vertebral artery), 4161205 (Hematoma of obstetric wound), 433915 (Burn of thigh), 4129406 (Open wound of hip region), 4052213 (Injury to blood vessels of lower limb), 4057170 (Injury of multiple muscles and tendons at shoulder and upper arm level), 138644 (Epidermal burn of lower limb), 4191821 (Multiple open wounds of shoulder and upper arm), 441709 (Subarachnoid hemorrhage following injury with open intracranial wound), 199089 (Laceration of cervix - obstetric), 196469 (Perforation of gallbladder), 4282857 (Closed fracture of upper limb), 4051597 (Multiple superficial injuries of wrist and hand), 81685 (Fracture of clavicle due to birth trauma), 4062231 (Injury of digital nerve of thumb), 373082 (Injury of trochlear nerve), 436250 (Open fracture of upper end of humerus), 136871 (Burn any degree involving 20-29 percent of body surface), 42536804 (Traumatic rupture of ulnar collateral ligament of elbow), 4108466 (Burn of respiratory tract), 4147025 (Epidermal burn of wrist and hand), 198495 (Fourth degree perineal laceration), 439152 (Fracture of malar or maxillary bones, open), 45766960 (Open fracture of phalanx of thumb), 4253764 (Superficial frostbite), 4050091 (Open wound of penis), 74772 (Open fracture of carpal bone), 198911 (Full thickness burn of trunk), 194807 (Injury of inferior vena cava), 36715773 (Injury of median nerve at upper arm level), 4320025 (Traumatic amputation of foot), 4108471 (Burn of digit of hand), 4056716 (Injury of multiple muscles and tendons at forearm level), 201990 (Injury of rectum with open wound into abdominal cavity), 195108 (Injury of multiple intra-abdominal organs without open wound into abdominal cavity), 438903 (Injury of external jugular vein), 436833 (Open fracture of metacarpal bone), 4199590 (Closed fracture of lower limb), 193345 (Pelvic organ injury with open wound into abdominal cavity), 4299128 (Full thickness burn), 138029 (Partial thickness burn of palm), 4054546 (Open wound of back wall of thorax), 4110164 (Injury of vein of upper limb), 4054671 (Corrosion of esophagus), 4053128 (Traumatic amputation, through shoulder), 4019730 (Injury to pancreas - open), 4052655 (Multiple superficial injuries of lower leg), 45757329 (Stress fracture of thoracic vertebra), 4153739 (Open wound of larynx and trachea), 441487 (Frostbite), 43022031 (Partial thickness burn of multiple sites of wrist), 4205651 (Partial thickness burn of multiple sites of hand), 4019263 (Concussion with less than 1 hour loss of consciousness), 42536803 (Traumatic rupture of radial collateral ligament of elbow), 4108318 (Burn of larynx and/or trachea), 436855 (Partial thickness burn of back of hand), 40490826 (Open fracture of base of skull), 433611 (Open fracture of multiple sites of metacarpus), 197435 (Crushing injury of multiple sites of lower limb), 4056457 (Injury of blood vessels at forearm level), 73349 (Injury of rectum without open wound into abdominal cavity), 4050086 (Open wound of thyroid), 73609 (Open fracture of clavicle), 320888 (Injury of internal jugular vein), 196856 (Crushing injury of trunk), 4009644 (Erb-Duchenne palsy as birth trauma), 4115176 (Pathological fracture - ankle and/or foot), 4043679 (Traumatic dislocation of joint), 440893 (Injury of axillary nerve), 4044351 (Injury of nerve of lower extremity), 4013161 (Open fracture sacrum), 4062390 (Injury of muscle(s) and tendon(s) of anterior muscle group at lower leg level),

		4086196 (Superficial injury of throat),<br>4062394 (Multiple open wounds of forearm),<br>4059253 (Injury of muscle and tendon of head),<br>439390 (Perineal laceration during delivery),<br>4058361 (Multiple superficial injuries of hip and/or thigh),<br>381733 (Multiple nerve injury),<br>261738 (Multiple burns of head and neck),<br>4117701 (Pathological fracture - shoulder),<br>4056438 (Injury of multiple blood vessels at neck level),<br>42710047 (Superficial frostbite of upper limb),<br>4209553 (Closed injury of thoracic trachea),<br>42536979 (Burn involving 10-19 percent of body surface, with 15-19 percent of body surface with full thickness burn),<br>42536978 (Burn involving 10-19 percent of body surface, with 10-14 percent of body surface with full thickness burn),<br>436248 (Open fracture of neck of femur),<br>197151 (Injury of abdominal aorta),<br>4164752 (Multiple open fractures of lower end of femur),<br>441423 (Open fracture of proximal end of radius),<br>435138 (Injury of femoral nerve),<br>45757330 (Stress fracture of lumbar vertebra),<br>135700 (Burn any degree involving 30-39 percent of body surface),<br>4055598 (Injury of greater saphenous vein at lower leg level),<br>4054884 (Multiple open wounds of hip and/or thigh),<br>4115179 (Fracture malunion - forearm),<br>192369 (Old laceration of cervix),<br>4013156 (Open fracture thoracic vertebra),<br>438022 (Crushing injury of external genitalia),<br>4059260 (Multiple open wounds of neck),<br>440812 (Pathological fracture - lower leg),<br>4090883 (Nasal septal caudal dislocation),<br>4082319 (Perinatal trauma),<br>78276 (Open fracture of ilium),<br>36716565 (Multiple open wounds of head),<br>435944 (Open fracture of shaft of radius),<br>432480 (Injury of multiple blood vessels of abdomen AND/OR pelvis),<br>438608 (Injury of axillary artery),<br>4152480 (Crush injury of head and neck),<br>4290881 (Birth injury to scalp),<br>4115180 (Fracture malunion - hand),<br>4062387 (Injury of muscle and tendon at thorax level),<br>73373 (Epidermal burn of upper limb),<br>4116723 (Injury of dorsalis pedis artery),<br>4057033 (Injury of nerves at ankle and foot level),<br>4050552 (Injury of vein at forearm level),<br>4020873 (Effects of vibration),<br>4050680 (Injury of blood vessels at ankle and foot level),<br>4177494 (Open fracture of lower limb),<br>37117266 (Injury of tendon of intrinsic muscle of thumb),<br>138315 (Burn any degree involving 40-49 percent of body surface),<br>4054873 (Injury of cutaneous sensory nerve at ankle and foot level),<br>78314 (Burn of thumb),<br>4056453 (Injury of superficial palmar arch),<br>435410 (Burn of palm),<br>42536768 (Superficial injury of posterior chest wall),<br>4062245 (Injury of lateral plantar nerve),<br>4054885 (Open wounds involving multiple regions of lower limb(s)),<br>4052505 (Multiple superficial injuries of shoulder and upper arm),<br>4050699 (Multiple superficial injuries of head),<br>76574 (Full thickness burn of lower leg),<br>4122189 (Fracture malunion - pelvis and/or thigh),<br>4115181 (Fracture malunion - lower leg),<br>4054872 (Injury of medial plantar nerve),<br>4020582 (Frostbite with tissue necrosis),<br>40490827 (Open fracture of fibula),<br>4058225 (Multiple superficial injuries of forearm),<br>4020015 (Superficial frostbite of knee and lower leg),<br>4071066 (Subarachnoid hemorrhage due to birth injury),<br>4052218 (Injury of peroneal artery),<br>36674185 (Burn of eye proper),<br>4180748 (Open fracture of tarsal bone),<br>75943 (Open fracture of pubis),<br>4050389 (Traumatic amputation, below elbow),<br>196284 (Burn of mouth and pharynx),

		134214 (Epidermal burn of wrist),<br>4118909 (Fracture malunion - upper arm),<br>4061839 (Sunburn of first degree),<br>438223 (Sunburn of second degree),<br>198819 (Obstetrical injury to pelvic organ),<br>4056460 (Injury of blood vessel(s) of thumb),<br>4052837 (Open wound of breast),<br>4015981 (Open fracture proximal femur, subtrochanteric),<br>432488 (Burn of back of hand),<br>77124 (Open fracture of scaphoid bone of wrist),<br>4347416 (Cerebral injury due to birth trauma),<br>4113927 (Injury of vein of trunk),<br>4059109 (Injury of cutaneous sensory nerve at forearm level),<br>4206244 (Open fracture of bone),<br>377680 (Facial nerve injury as birth trauma),<br>42536767 (Superficial injury of anterior wall of thorax),<br>4309930 (Fractures involving thorax with lower back and pelvis),<br>4309487 (Crushing injuries of thorax with abdomen, lower back and pelvis with limb(s)),<br>4056718 (Injury of adductor muscle and tendon of thigh),<br>260246 (Injury of bronchus without open wound into thoracic cavity),<br>434769 (Flail chest),<br>77405 (Open fracture of acetabulum),<br>4062246 (Injury of multiple nerves at ankle and foot level),<br>4020584 (Frostbite with tissue necrosis of hip and thigh),<br>439093 (Traumatic lesion during delivery),<br>197343 (First degree perineal laceration),<br>29979 (Crushing injury of neck),<br>4020304 (Frostbite with tissue necrosis of ankle and foot),<br>4308853 (Traumatic amputations involving multiple body regions),<br>4208507 (Open injury of thoracic trachea),<br>441443 (Injury of superior vena cava),<br>138616 (Epidermal burn of hand),<br>43021547 (Full thickness burn of multiple sites of wrist),<br>43021544 (Full thickness burn of multiple sites of hand),<br>4051298 (Traumatic amputation, through wrist),<br>4016550 (Open injury of pleura),<br>4003357 (Epidermal burn of forehead),<br>4001839 (Epidermal burn of cheek),<br>192957 (Perforation of bile duct),<br>43021545 (Burn of multiple sites of wrist),<br>43021543 (Burn of multiple sites of hand),<br>35624484 (Open fracture of coccyx),<br>4059242 (Injury of superficial vein at shoulder and upper arm level),<br>4210437 (Open multiple fracture of thoracic spine),<br>4144835 (Crushing injury of chest),<br>4027522 (Injury of ovary without open wound into abdominal cavity),<br>195976 (Burn of gastrointestinal tract),<br>75689 (Contusion of breast),<br>444063 (Injury of urethra),<br>433073 (Epidermal burn of back of hand),<br>198492 (Second degree perineal laceration),<br>4169252 (Internal injury of abdominal organ),<br>4106203 (Gastrointestinal and digestive injury),<br>4051740 (Multiple superficial injuries of ankle and foot),<br>4051136 (Multiple open wounds of thoracic wall),<br>4009453 (Open fracture of thumb metacarpal),<br>378048 (Burn of ear),<br>198049 (Partial thickness burn of multiple sites of lower limb),<br>138292 (Epidermal burn of thigh),<br>133669 (Epidermal burn of lower leg),<br>77112 (Open fracture of scapula),<br>4174885 (Traumatic pneumohemothorax),<br>4057350 (Crushing injury of skull),<br>4056458 (Injury of multiple blood vessels of upper extremity),<br>72516 (Burn of knee),<br>438290 (Partial thickness burn of neck),<br>435123 (Injury of nerve roots AND/OR spinal plexus of multiple sites),<br>4008222 (Open fracture lumbar vertebra),<br>434499 (Open fracture of sternum),<br>4246695 (Open wound of head AND/OR neck),<br>381445 (Avulsion of eye),<br>136596 (Epidermal burn of palm),<br>4054879 (Injury of multiple nerves at shoulder and upper arm level),

© 2024 Lee DY et al. *JAMA Network Open*

		4003358 (Partial thickness burn of forehead),<br>4001840 (Partial thickness burn of cheek),<br>4050553 (Injury of multiple blood vessels at forearm level),<br>73362 (Crushing injury of multiple sites of upper limb),<br>4059856 (Superficial burn of a single finger),<br>76870 (Full thickness burn of wrist),<br>42536848 (Multiple injuries of lower leg),<br>37396333 (Hand muscle strain),<br>36674775 (Full thickness burn of finger),<br>434544 (Full thickness burn of thigh),<br>134243 (Partial thickness burn of knee),<br>4308852 (Dislocations, sprains and strains involving multiple regions of lower limb(s)),<br>4059110 (Injury of multiple nerves at forearm level),<br>4057038 (Injury of peripheral nerves of neck),<br>440913 (Crushing injury of multiple sites),<br>45766970 (Pathological fracture of humerus due to neoplastic disease),<br>4062253 (Injury of multiple blood vessels at shoulder and upper arm level),<br>4059261 (Multiple open wounds of abdomen, lower back and pelvis),<br>4057798 (Corrosion of ankle and foot),<br>433333 (Open fracture of shaft of ulna),<br>45766818 (Stress fracture of hand),<br>4059858 (Superficial burn of the thumb and finger(s)),<br>4057181 (Multiple injuries of neck),<br>4056571 (Injury of multiple blood vessels at hip and thigh level),<br>441698 (Multiple open fractures of cervical vertebrae),<br>4020585 (Frostbite with tissue necrosis involving multiple body regions),<br>200871 (Injury of multiple intra-abdominal organs with open wound into abdominal cavity),<br>4160362 (Injury of thoracic nerve root),<br>4151955 (Open flail chest),<br>4056003 (Multiple superficial injuries of abdomen, lower back and pelvis),<br>4055599 (Injury of multiple blood vessels at lower leg level),<br>198286 (Injury of heart with open wound into thorax),<br>4201174 (Injury of lung),<br>4079931 (Open fracture of upper limb),<br>4071588 (Tentorial tear due to birth trauma),<br>442005 (Full thickness burn of back of hand),<br>440890 (Cervical sympathetic nerve injury),<br>4102360 (Nerve root and plexus compressions in neoplastic disease),<br>4071595 (Fracture of femur due to birth trauma),<br>4059869 (Corrosion of third degree of wrist and hand),<br>4057032 (Injury of multiple nerves at lower leg level),<br>4054978 (Traumatic amputation of external genital organs),<br>194791 (Injury of uterus with open wound into abdominal cavity),<br>45770877 (Stress fracture of cervical vertebra),<br>4345918 (Wound myiasis),<br>4281541 (Open pertrochanteric fracture),<br>4108636 (Superficial injury of head and neck),<br>4016548 (Closed injury of pleura),<br>45767042 (Pathological fracture of humerus due to osteoporosis),<br>439211 (Injury of musculocutaneous nerve),<br>76595 (Full thickness burn of thumb),<br>43022030 (Epidermal burn of multiple sites of hand),<br>43021546 (Epidermal burn of multiple sites of wrist),<br>4309488 (Traumatic amputation of both feet),<br>4206874 (Intracranial injury with prolonged coma without open wound),<br>4115182 (Fracture malunion - ankle and/or foot),<br>4053591 (Injury of small intestine),<br>4003359 (Burn of cheek),<br>4001838 (Burn of forehead),<br>435641 (Birth trauma),<br>134248 (Partial thickness burn of thumb),<br>46284319 (Skull injury due to birth trauma),<br>4059857 (Superficial burn of more than one finger),<br>440260 (Pulmonary blood vessel injury),<br>4057031 (Injury of multiple nerves at hip and thigh level),<br>4056567 (Injury of lesser saphenous vein at lower leg level),<br>4056454 (Injury of deep palmar arch),<br>442553 (Injury of bladder),<br>134521 (Partial thickness burn of scalp),<br>4309484 (Dislocations, sprains and strains involving multiple regions of upper limb(s) and lower limb(s)),<br>4308854 (Injuries of muscles and tendons involving multiple body regions),

		4252893 (Open wound of multiple sites of one upper limb),<br>4220631 (Injury of kidney),<br>4134318 (Fractured nasal bones),<br>4105009 (Nerve root and plexus compressions in spondylosis),<br>4062393 (Multiple injuries of thorax),<br>4057179 (Multiple injuries of head),<br>4051440 (Traumatic amputation, through hip),<br>4048139 (Cerebral edema due to birth injury),<br>198030 (Burn of internal organ),<br>76573 (Full thickness burn of hand),<br>4209557 (Closed fractures involving head with neck),<br>4055326 (Corrosion of mouth and pharynx),<br>132771 (Burn any degree involving 90 percent OR more of body surface),<br>4309491 (Injuries of brain and cranial nerves with injuries of nerves and spinal cord at neck level),<br>4308551 (Dislocations, sprains and strains involving multiple regions of upper limb(s)),<br>4198759 (Bilateral traumatic amputation of upper limbs),<br>78574 (Open fracture of multiple ribs),<br>75053 (Fracture of bone),<br>4305587 (Epidermal burn of neck),<br>4220695 (Open fracture of vertebral column),<br>4206883 (Closed fractures involving multiple regions of both lower limbs),<br>4058509 (Traumatic amputation of part of thorax),<br>4309934 (Injuries of blood vessels involving multiple body regions),<br>4056722 (Open wounds involving thorax with abdomen, lower back and pelvis),<br>441479 (Injury of cutaneous sensory nerve of lower limb),<br>197089 (Peripheral nerve injury due to birth trauma),<br>195398 (Injury of blood vessels of head AND/OR neck),<br>142004 (Partial thickness burn of ear),<br>37116518 (Multiple traumatic dislocation of finger),<br>4215380 (Open fracture dislocation of joint of shoulder girdle),<br>4129393 (Fracture of cervical spine),<br>4059703 (Corrosion of head and neck),<br>4052969 (Open wound of pelvic region),<br>4020302 (Frostbite with tissue necrosis of wrist and hand),<br>40483816 (Bilateral traumatic amputation of lower limbs),<br>4278672 (Fracture of forearm),<br>4206871 (Open multiple fractures of clavicle and/or scapula and/or humerus),<br>4103235 (Phrenic nerve paralysis as birth trauma),<br>4054648 (Corrosion of wrist and hand),<br>4016552 (Multiple injuries of intrathoracic organs),<br>444105 (Burn any degree involving 70-79 percent of body surface),<br>436283 (Full thickness burn of palm),<br>315113 (Injury of multiple blood vessels of thorax),<br>197709 (Injury of uterus without open wound into abdominal cavity),<br>195978 (Burn of multiple sites of lower limb),<br>142033 (Injury of cutaneous sensory nerve of upper limb),<br>133914 (Epidermal burn of thumb),<br>37116504 (Multiple injuries of forearm),<br>36715634 (Traumatic dislocation of multiple cervical vertebra),<br>4308552 (Traumatic amputation of both hands),<br>4057180 (Multiple superficial injuries of neck),<br>4055726 (Multiple superficial injuries of thorax),<br>4052970 (Open wounds involving head with neck),<br>4016958 (Concussion with 1-24 hours loss of consciousness),<br>138622 (Burn of lip),<br>135695 (Partial thickness burn of chin),<br>42536875 (Injury of plantar artery of foot),<br>4166905 (Superficial injury of breast),<br>4152475 (Chemical burn of internal organ),<br>4140092 (Fractures of multiple bones of lower limb),<br>4047853 (Scalp bruising due to birth trauma),<br>440825 (Fracture of shaft of femur),<br>196475 (Damage to pelvic organs AND/OR tissues following molar AND/OR ectopic pregnancy),<br>135984 (Burn involving 20-29 percent of body surface, with 20-29 percent of body surface with full thickness burn),<br>27438 (Burn of esophagus),<br>45766971 (Pathological fracture of hand due to neoplastic disease),<br>40644369 (Full thickness burn of multiple sites of lower limb),<br>37110536 (Injury of cutaneous sensory nerve at lower leg level),<br>4206885 (Closed fracture involving thorax wth lower back and pelvis and limbs),<br>4113015 (Crush injury of respiratory structure),

		4055319 (Corrosion of 2nd degree of hip and lower limb, except ankle and foot),<br>4050681 (Injury of multiple blood vessels at ankle and foot level),<br>4048141 (Birth injury to face),<br>444403 (Injury of bronchus with open wound into thoracic cavity),<br>442612 (Burn any degree involving 50-59 percent of body surface),<br>259988 (Sinus barotrauma),<br>45771402 (Stress fracture of humerus),<br>44784364 (Spine injury due to birth trauma),<br>42539093 (Frostbite of multiple body regions),<br>40640002 (Epidermal burn of multiple sites of lower limb),<br>36675123 (Frostbite with tissue necrosis of upper limb),<br>4309486 (Crushing injuries involving multiple regions of upper limb(s) with lower limb(s)),<br>4268004 (Injury of pancreas),<br>4175616 (Fracture of shaft of humerus),<br>4054455 (Injury of intra-abdominal organ(s) with pelvic organ(s)),<br>443773 (Damage to pelvic joints AND/OR ligaments during delivery),<br>138312 (Burn of scalp),<br>79998 (Full thickness burn of the thumb and finger(s)),<br>26286 (Burn of neck),<br>43530815 (Traumatic injury by site),<br>4276036 (Fracture of acetabulum),<br>4210442 (Open fractures involving multiple regions of both lower limbs),<br>4174520 (Fracture of vertebral column),<br>4138414 (Fracture of calcaneus),<br>4110336 (Anterior spinal and vertebral artery compression syndromes),<br>4019264 (Concussion with more than 24 hours loss of consciousness and return to pre-existing conscious level),<br>200293 (Crushing injury of hip),<br>135710 (Burn involving 30-39 percent of body surface, with 20-29 percent of body surface with full thickness burn),<br>135414 (Epidermal burn of knee),<br>75942 (Open fracture of rib),<br>(),
Methylphenidate	RxNorm	705944 (methylphenidate),<br>42943598 (Methylphenidate 10 MG Oral Tablet [PENID]),<br>42943576 (Methylphenidate 18 MG Extended Release Oral Tablet [CONCERTA OROS]),<br>42943592 (Methylphenidate 27 MG Extended Release Oral Tablet [CONCERTA OROS]),<br>42943580 (Methylphenidate 20 MG Extended Release Oral Capsule [Metadate CD]),<br>42943600 (Methylphenidate 10 MG Extended Release Oral Capsule [Metadate CD]),<br>42943588 (Methylphenidate 36 MG Extended Release Oral Tablet [CONCERTA OROS]),<br>42943584 (Methylphenidate 30 MG Extended Release Oral Capsule [Metadate CD]),<br>42943578 (Methylphenidate 54 MG Extended Release Oral Tablet [CONCERTA OROS]),<br>40236419 (methylphenidate hydrochloride 10 MG Oral Tablet),<br>21119719 (Methylphenidate 20 MG Extended Release Oral Capsule [Medikinet]),<br>40843492 (Methylphenidate 10 MG Extended Release Oral Capsule [Medikinet]),<br>42943601 (Methylphenidate 10 MG Extended Release Oral Capsule [BISPHENTIN]),<br>21080489 (Methylphenidate 30 MG Extended Release Oral Capsule [Medikinet]),<br>42943585 (Methylphenidate 30 MG Extended Release Oral Capsule [BISPHENTIN]),<br>40843487 (Methylphenidate 5 MG Extended Release Oral Capsule [Medikinet]),<br>42943590 (Methylphenidate 60 MG Extended Release Oral Capsule [BISPHENTIN]),
Anti-ADHD drugs	RxNorm	40222068 (bupropion hydrochloride 150 MG Extended Release Oral Tablet [Wellbutrin]),<br>44048315 (Bupropion 300 MG Extended Release Oral Tablet [Wellbutrin XL]),<br>750982 (bupropion),<br>42707371 (clonidine hydrochloride 0.15 MG Oral Tablet),<br>40227886 (clonidine hydrochloride 0.1 MG Extended Release Oral Tablet [Kapvay]),<br>19098491 (atomoxetine 40 MG Oral Capsule [Strattera]),<br>742228 (atomoxetine 25 MG Oral Capsule [Strattera]),<br>44125936 (Bupropion 150 MG Extended Release Oral Tablet [Wellbutrin XL]),<br>742185 (atomoxetine),<br>742230 (atomoxetine 60 MG Oral Capsule [Strattera]),<br>1398937 (clonidine),<br>742227 (atomoxetine 18 MG Oral Capsule [Strattera]),<br>742226 (atomoxetine 10 MG Oral Capsule [Strattera]),<br>40169601 (clonidine hydrochloride 0.15 MG/ML Injectable Solution),<br>742266 (atomoxetine 80 MG Oral Capsule [Strattera])

SSRI	RxNorm	715939 (escitalopram),<br>42944898 (Escitalopram 10 MG Oral Tablet [ETALOP]),<br>715965 (escitalopram 10 MG Oral Tablet [Lexapro]),<br>715966 (escitalopram 20 MG Oral Tablet [Lexapro]),<br>19102734 (escitalopram 5 MG Oral Tablet [Lexapro]),<br>755695 (fluoxetine),<br>19077462 (fluoxetine 10 MG Oral Capsule),<br>42940706 (Fluoxetine 10 MG Oral Capsule [FROPINE]),<br>43204248 (Fluoxetine 20 MG Disintegrating Oral Tablet [Prozac]),<br>19004895 (fluoxetine 20 MG Oral Capsule [Prozac]),<br>2053758 (fluoxetine 90 MG Extended Release Oral Capsule [PROZAC WEEKLY] by Lilly),<br>739138 (sertraline),<br>19037642 (sertraline 100 MG Oral Tablet [Zoloft]),<br>19037684 (sertraline 50 MG Oral Tablet [Zoloft]),<br>722031 (paroxetine),<br>35604589 (paroxetine mesylate 10 MG Oral Tablet),<br>19115249 (paroxetine hydrochloride 12.5 MG Extended Release Oral Tablet),<br>35604576 (paroxetine hydrochloride 20 MG Oral Tablet),<br>722156 (paroxetine hydrochloride 25 MG Extended Release Oral Tablet)
Fluoxetine	RxNorm	755695 (fluoxetine),<br>19077462 (fluoxetine 10 MG Oral Capsule),<br>42940706 (Fluoxetine 10 MG Oral Capsule [FROPINE]),<br>43204248 (Fluoxetine 20 MG Disintegrating Oral Tablet [Prozac]),<br>19004895 (fluoxetine 20 MG Oral Capsule [Prozac]),<br>2053758 (fluoxetine 90 MG Extended Release Oral Capsule [PROZAC WEEKLY] by Lilly),
Escitalopram	RxNorm	715939 (escitalopram),<br>42944898 (Escitalopram 10 MG Oral Tablet [ETALOP]),<br>715965 (escitalopram 10 MG Oral Tablet [Lexapro]),<br>715966 (escitalopram 20 MG Oral Tablet [Lexapro]),<br>19102734 (escitalopram 5 MG Oral Tablet [Lexapro]),
Antidepressants	RxNorm	715939 (escitalopram),<br>42944898 (Escitalopram 10 MG Oral Tablet [ETALOP]),<br>715965 (escitalopram 10 MG Oral Tablet [Lexapro]),<br>715966 (escitalopram 20 MG Oral Tablet [Lexapro]),<br>19102734 (escitalopram 5 MG Oral Tablet [Lexapro]),<br>755695 (fluoxetine),<br>19077462 (fluoxetine 10 MG Oral Capsule),<br>42940706 (Fluoxetine 10 MG Oral Capsule [FROPINE]),<br>43204248 (Fluoxetine 20 MG Disintegrating Oral Tablet [Prozac]),<br>19004895 (fluoxetine 20 MG Oral Capsule [Prozac]),<br>2053758 (fluoxetine 90 MG Extended Release Oral Capsule [PROZAC WEEKLY] by Lilly),<br>739138 (sertraline),<br>19037642 (sertraline 100 MG Oral Tablet [Zoloft]),<br>19037684 (sertraline 50 MG Oral Tablet [Zoloft]),<br>722031 (paroxetine),<br>35604589 (paroxetine mesylate 10 MG Oral Tablet),<br>19115249 (paroxetine hydrochloride 12.5 MG Extended Release Oral Tablet),<br>35604576 (paroxetine hydrochloride 20 MG Oral Tablet),<br>722156 (paroxetine hydrochloride 25 MG Extended Release Oral Tablet)<br>743670 (venlafaxine),<br>19134632 (venlafaxine 37.5 MG Extended Release Oral Capsule),<br>19103819 (venlafaxine 75 MG Extended Release Oral Capsule),<br>717607 (desvenlafaxine),<br>1593114 (desvenlafaxine succinate 50 MG Extended Release Oral Tablet),<br>1593109 (desvenlafaxine) succinate 100 MG Extended Release Oral Tablet),<br>715259 (duloxetine),<br>19121375 (duloxetine 30 MG Extended Release Oral Capsule),<br>715292 (duloxetine hydrochloride 60 MG Extended Release Oral Capsule),<br>750982 (bupropion),<br>40222060 (bupropion hydrochloride 100 MG Oral Tablet),<br>40222065 (bupropion hydrochloride 150 MG Oral Tablet),<br>40222084 (bupropion hydrochloride 300 MG Oral Tablet),<br>725131 (mirtazapine),<br>19112586 (mirtazapine 7.5 MG Oral Tablet),<br>725178 (mirtazapine 15 MG Oral Tablet),<br>19070747 (mirtazapine 15 MG Disintegrating Oral Tablet),<br>725180 (mirtazapine 30 MG Oral Tablet),<br>19070748 (mirtazapine 30 MG Disintegrating Oral Tablet),<br>778268 (imipramine),<br>778296 (imipramine hydrochloride 25 MG Oral Tablet),<br>721724 (nortriptyline),

		721728 (nortriptyline 10 MG Oral Tablet),<br>19021720 (nortriptyline 25 MG Oral Tablet),<br>738156 (doxepin),<br>40173384 (doxepin 3 MG Oral Tablet),<br>40173388 (doxepin 6 MG Oral Tablet),<br>710062 (amitriptyline),<br>42931449 (Amitriptyline 5 MG Oral Tablet),<br>40162672 (amitriptyline hydrochloride 10 MG Oral Tablet),<br>40162717 (amitriptyline hydrochloride 25 MG Oral Tablet),

© 2024 Lee DY et al. *JAMA Network Open*

eMethods. Study design and database

The HIRA database contains complete health information on the South Korean population, including anonymized personal identifiers, demographics, diagnoses, and medical procedures and medications in the national reimbursement lists. The HIRA database was standardized with the Observational Medical Outcomes Partnership common data model (OMOP-CDM) version 5.3.[1, 2]

This database is the national health insurance claims data of the Republic of Korea. The Republic of Korea is implementing a single public health insurance system, so about 98% of the entire population is covered by the insurance. This database was created by extracting all patient data diagnosed with ADHD or prescribed methylphenidate from the National Health Insurance data warehouse of the Health Insurance Review and Assessment Service from January 2016 to February 2021. This database includes information on diagnosis, surgery and treatment history, lab exam history, prescription, and cost information that occurs when the patient uses medical care. Prescription includes information on the usage, dosage, and quantity of medications and other treatment materials. Prescription information does not include actual dispensing information in this database. This database was converted from the source format to the OMOP common data model version 5.3. It complies with the standards for each domain of OMOP standardized vocabulary, with diagnosis code mapped to SNOMED, drug to RxNorm, device to SNOMED, measurement to SNOMED or LOINC, and procedure to LOINC, CPT4, HCPCS, etc.

We pre-specified this study before execution and registered a protocol with the EU Post-Authorization Studies register under EUPAS103757 (see Supplement 1). According to this protocol, the study package for the entire analytical process was released in an online repository for transparency of analyses (https://github.com/ABMI/ASSURE-Extend).

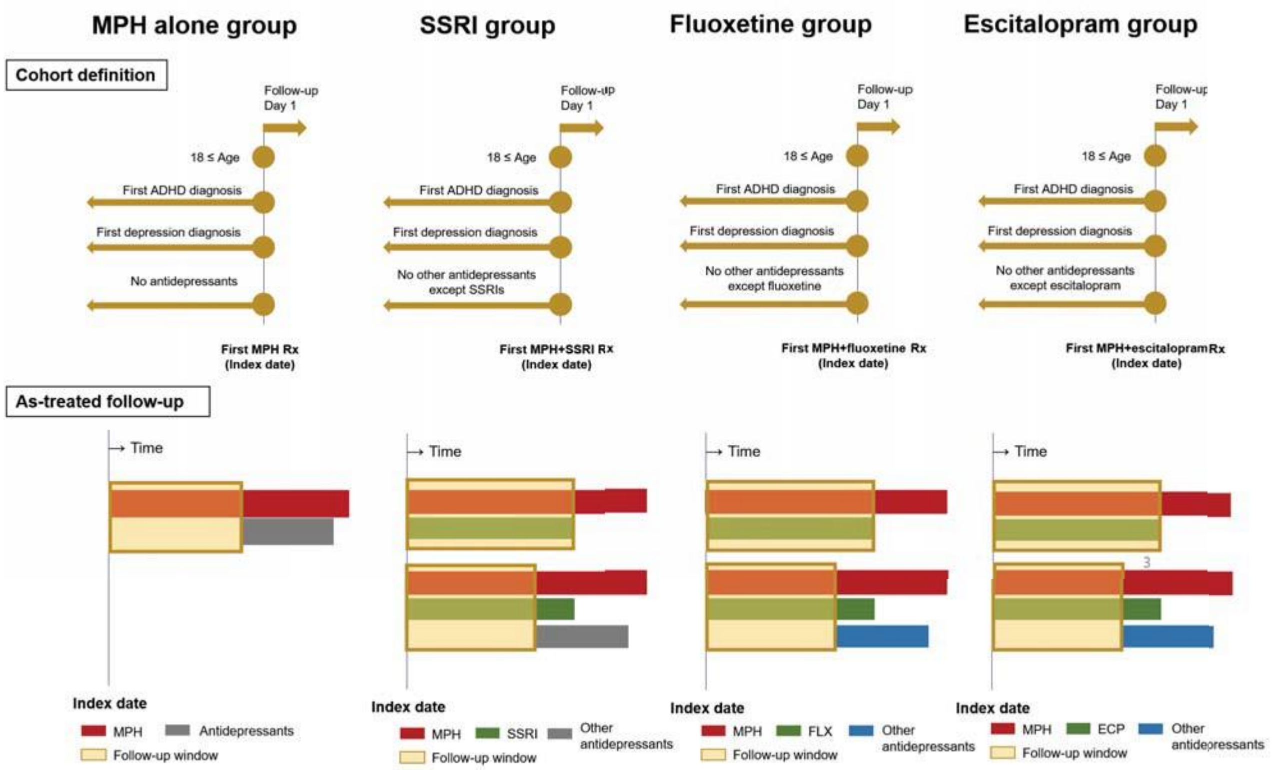

eFigure 1. Schematic visualization for the cohort definition and as-treated follow-up strategy

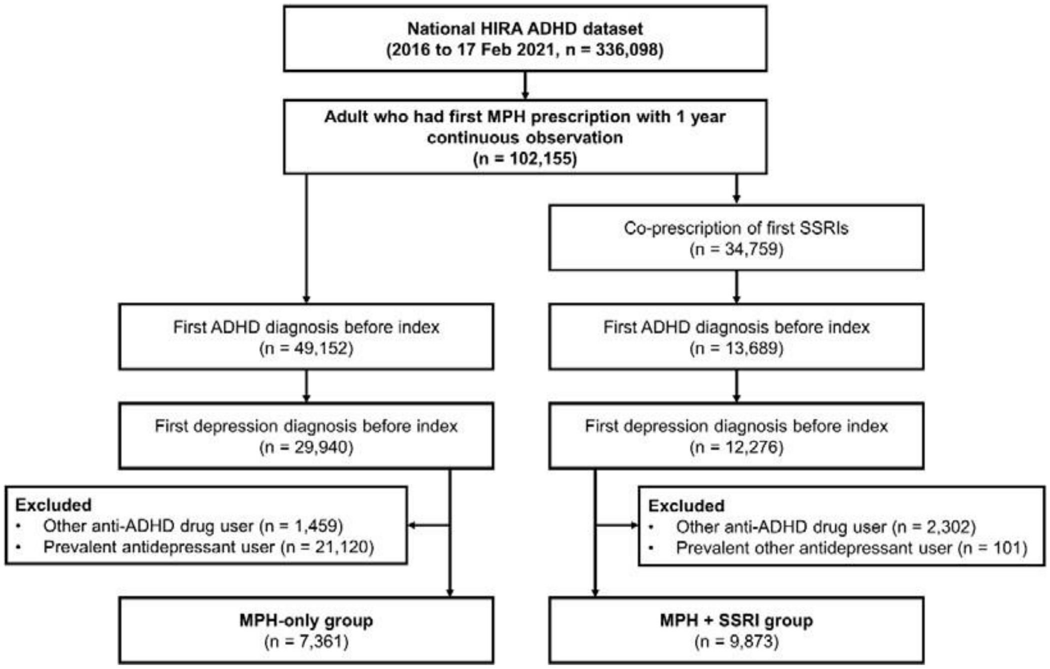

eFigure 2. Flow diagram between the SSRI group and the MPH-only group

eTable 1. Baseline characteristics, comorbidities, and concomitant drugs in adult ADHD patients with depression before the propensity score matching

Characteristics	SSRI (n=9,873), n (%)	MPH-only (n=7,361), n (%)	aSMD	Fluoxetine (n=2,791), n (%)	Escitalopram (n=5,150), n (%)	aSMD
**Socio-demographics**						
Male	4,364 (44.2)	3,791 (51.5)	0.15	1,027 (36.8)	2,436 (47.3)	0.21
Female	5,509 (55.8)	3570 (48.5)	0.15	1,764 (63.2)	2,714 (52.7)	0.21
18–39 years	8,352 (84.6)	6,279 (85.3)	0.01	2,400 (86.0)	4,331 (84.1)	0.06
40–64 years	1,353 (13.7)	957 (13.0)	0.04	380 (13.6)	695 (13.5)	0.02
≥65 years	168 (1.7)	125 (1.7)	0.00	11 (0.4)	124 (2.4)	0.30
Race, Korean	9,873 (100.0)	7,361 (100.0)	0.00	1,820 (100.0)	1,820 (100.0)	0.00
**Index year**						
2017	1,165 (11.8)	795 (10.8)	0.03	368 (13.2)	567 (11.0)	0.07
2018	1,925 (19.5)	1,303 (17.7)	0.05	548 (19.6)	968 (18.8)	0.02
2019	2,824 (28.6)	2,186 (29.7)	0.03	819 (29.3)	1,483 (28.8)	0.01
2020	3,959 (40.1)	3,077 (41.8)	0.03	1,056 (37.8)	2,132 (41.4)	0.07
**Psychiatric comorbidity**						
Substance use disorder	316 (3.2)	169 (2.3)	0.06	106 (3.8)	144 (2.8)	0.06
Conduct disorder	79 (0.8)	81 (1.1)	0.01	31 (1.1)	36 (0.7)	0.04
Personality disorder	257 (2.6)	118 (1.6)	0.07	87 (3.1)	98 (1.9)	0.08
Autism spectrum disorder	59 (0.6)	44 (0.6)	0.00	20 (0.7)	31 (0.6)	0.01
Intellectual disability	79 (0.8)	74 (1.0)	0.01	31 (1.1)	36 (0.7)	0.04
**Medication use**						
Anticholinergics	158 (1.6)	125 (1.7)	0.00	42 (1.5)	72 (1.4)	0.00
Antiepileptics	1,550 (15.7)	758 (10.3)	0.16	402 (14.4)	731 (14.2)	0.01
Antipsychotics	2,675 (27.1)	1,406 (19.1)	0.19	762 (27.3)	1,277 (24.8)	0.06
Anxiolytics	4,561 (46.2)	1,906 (25.9)	0.43	1,186 (42.5)	2,359 (45.8)	0.07

ADHD: attention-deficit/hyperactivity disorder; PS: propensity score; MPH: methylphenidate; SSRI: selective serotonin reuptake inhibitor; aSMD: absolute standardized mean difference; FLX: fluoxetine; ECP: escitalopram.

© 2024 Lee DY et al. *JAMA Network Open*

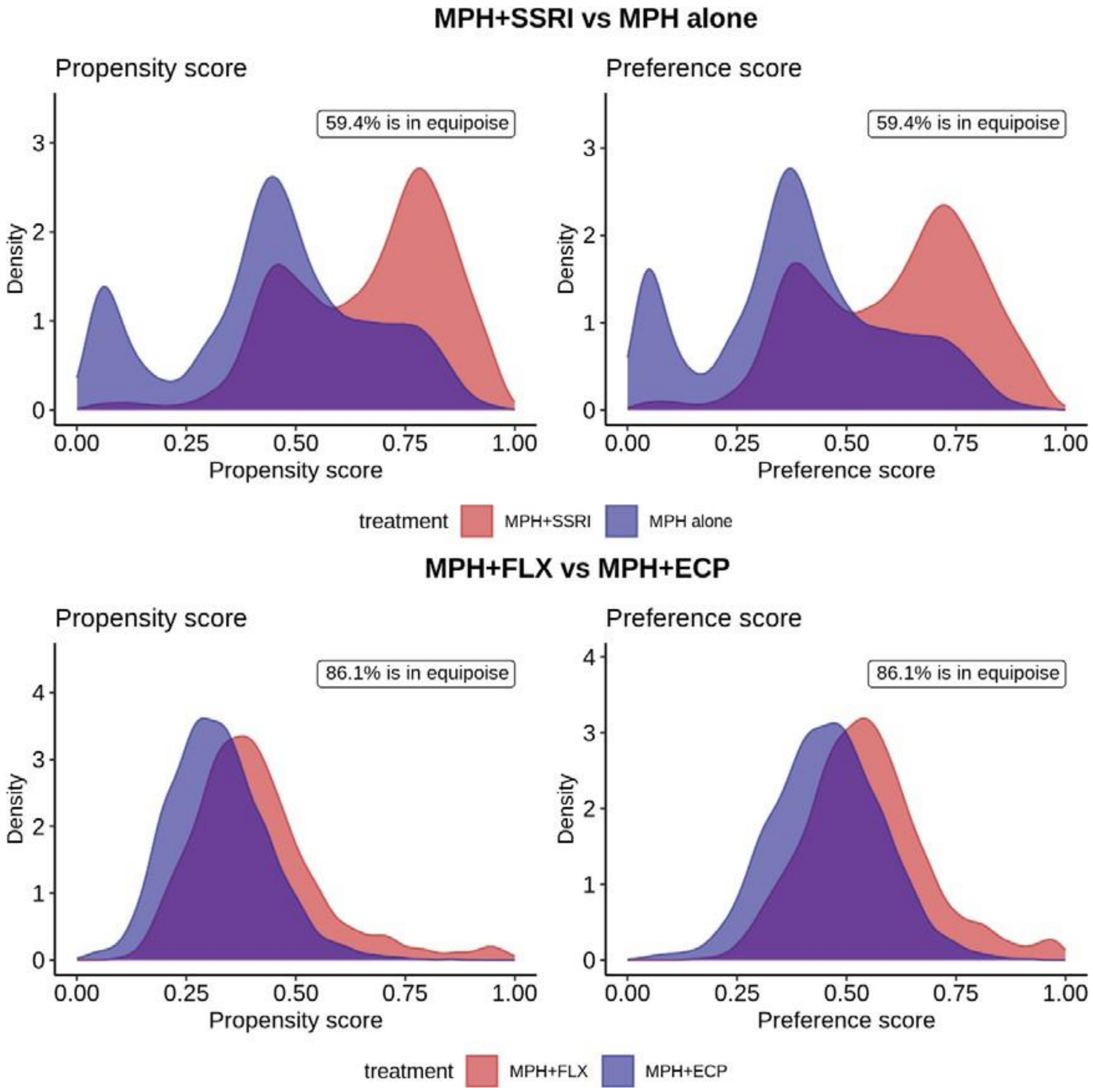

eFigure 3. Empirical equipoise between the comparison groups.
MPH: methylphenidate; SSRI: selective serotonin reuptake inhibitor; FLX: fluoxetine; ECP: escitalopram; SER: sertraline; Preference score: prevalence-adjusted propensity score[3]

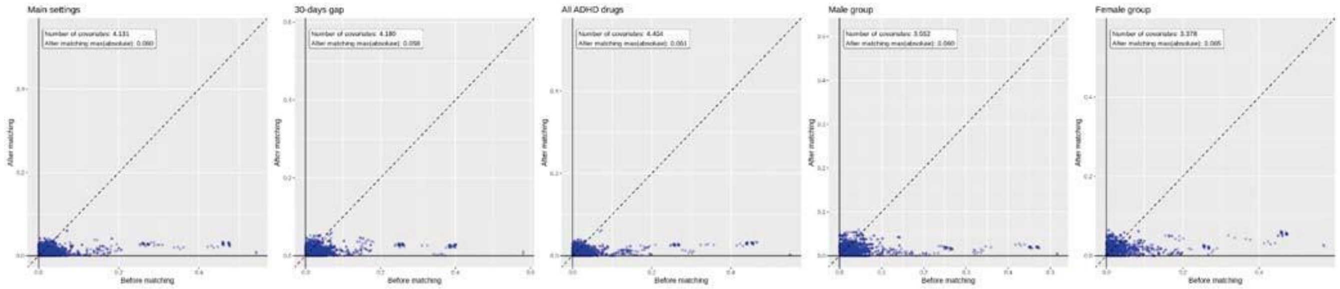

eFigure 4. Scatter plots between before and after the propensity score adjustment between the SSRI and MPH-only groups

© 2024 Lee DY et al. *JAMA Network Open*

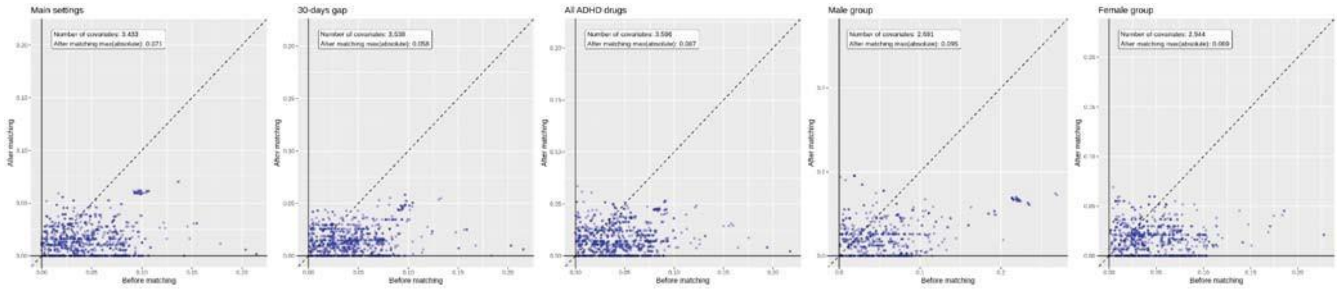

eFigure 5. Scatter plots between before and after the propensity score adjustment between the fluoxetine and escitalopram groups

© 2024 Lee DY et al. *JAMA Network Open*

eTable 2. Incidence of outcome events between the SSRI and MPH-only groups

Outcomes	SSRI group (n = 5,181)			MPH-only group (n = 5,181)		
	Number of events	Person-years	Incidence rate[§]	Number of events	Person-years	Incidence rate[§]
**Primary endpoint**						
Mania	7	2 250.7	3.11	10	1426.0	7.02
Anxiety disorder	351	1 371.8	256.05	275	923.2	298.09
Sleep disorder	350	1 692.9	206.89	268	1098.0	244.25
Tic disorder	41	2 215.7	18.52	29	1407.0	20.63
Hospitalization	34	2 256.6	15.08	35	1418.7	24.69
**Secondary endpoint**						
Tremor	20	1 371.8	8.95	22	923.2	15.52
Headache	14	2 245.7	6.24	19	1418.2	13.41
Seizure	50	2 185.3	22.90	33	1391.0	23.74
Dizziness	16	2 244.3	7.13	9	1 421.0	6.34
Arrhythmia	31	2 220.3	13.97	21	1 410.5	14.90
Hypertension	36	2 229.2	16.16	19	1418.3	13.41
Abdominal pain	6	2 265.8	2.65	9	1 428.2	6.31
Constipation	48	2 247.7	21.37	47	1 418.9	33.15
Nausea vomiting	55	2 245.6	24.51	37	1 424.0	26.00
Anemia	5	2 259.0	2.21	2	1 431.6	1.40
Hyperlipidemia	18	2 247.9	8.01	13	1 421.5	9.15
Traumatic injury	12	2 263.5	5.31	6	1 430.8	4.20
**Negative control outcome**	5	2 257.5	2.21	2	1 432.7	1.40

SSRI: selective serotonin reuptake inhibitor; MPH: methylphenidate; [§]Incidence rate was calculated as case per 1 000 person-years; Negative control outcome indicates respiratory tract infection.

© 2024 Lee DY et al. *JAMA Network Open*

eTable 3. Incidence of outcome events between the fluoxetine and escitalopram groups

Outcomes	Fluoxetine group (n = 2,577)			Escitalopram group (n = 2,577)		
	Number of events	Person-years	Incidence rate[§]	Number of events	Person-years	Incidence rate[§]
**Primary endpoint**						
Mania	4	966.7	4.14	5	991.9	5.04
Anxiety disorder	156	567.3	275.19	145	575.0	252.33
Sleep disorder	169	704.3	240.13	186	725.2	256.66
Tic disorder	22	944.2	23.32	25	966.8	25.88
Hospitalization	10	968.4	10.33	18	986.5	18.26
**Secondary endpoint**						
Tremor	11	956.6	11.51	6	991.5	6.06
Headache	7	961.0	7.29	8	985.5	8.12
Seizure	21	949.7	22.13	22	967.6	22.75
Dizziness	3	959.7	3.13	5	982.8	5.09
Arrhythmia	15	957.6	15.67	11	986.1	11.16
Hypertension	4	963.1	4.16	11	980.3	11.23
Abdominal pain	7	967.0	7.24	5	992.5	5.04
Constipation	26	963.4	27.01	23	983.7	23.40
Nausea vomiting	30	957.1	31.37	38	980.0	38.81
Anemia	1	969.1	1.03	3	991.2	3.03
Hyperlipidemia	2	968.0	2.07	5	989.3	5.06
Traumatic injury	3	969.6	3.10	3	993.3	3.02
**Negative control outcome**	2	969.4	2.06	3	991.4	3.03

[§]Incidence rate was calculated as case per 1 000 person-years; Negative control outcome indicates respiratory tract infection.

© 2024 Lee DY et al. *JAMA Network Open*

eTable 4. Risk of outcome events between the SSRI and MPH alone groups in the subgroup analysis by sex

Outcomes	Male subgroup			Female subgroup		
	Incidence Rate[§]			Incidence Rate[§]		
	SSRI (n = 2,509)	MPH–only (Reference, n = 2,509)	HR [95% CI]	SSRI (n = 2,623)	MPH–only (Reference, n = 2,623)	HR [95% CI]
**Primary endpoint**						
Mania	2.67	3.73	0.79 [0.73–1.15]	3.65	6.49	0.82 [0.19–3.47]
Anxiety disorder	230.41	271.48	0.92 [0.73–1.16]	260.80	308.29	0.99 [0.79–1.25]
Sleep disorder	181.49	216.12	0.91 [0.73–1.15]	240.29	268.61	1.07 [0.85–1.34]
Tic disorder	18.40	19.06	1.09 [0.56–2.17]	29.57	24.45	1.51 [0.82–2.87]
Hospitalization	17.00	25.05	0.78 [0.42–1.48]	12.72	17.82	0.85 [0.38–1.93]
**Secondary endpoint**						
Tremor	2.70	12.52	**0.23 [0.05–0.77]** ‡	9.08	17.84	0.48 [0.20–1.16]
Headache	5.37	10.03	0.56 [0.18–1.61]	7.27	22.85	**0.35 [0.14–0.83]** ‡
Seizure	23.00	24.44	1.03 [0.57–1.91]	16.53	22.91	0.84 [0.41–1.74]
Dizziness	5.37	7.54	0.74 [0.23–2.39]	5.49	4.86	1.27 [0.33–6.11]
Arrhythmia	5.44	7.55	0.78 [0.24–2.49]	17.42	17.98	1.13 [0.54–2.47]
Hypertension	10.81	15.01	0.76 [0.34–1.72]	19.44	8.12	**3.24 [1.31–9.73]** ‡
Abdominal pain	0.89	4.98	0.20 [0.01–1.37]	4.53	8.09	0.65 [0.18–2.39]
Constipation	8.95	21.24	0.50 [0.22–1.07]	24.77	38.95	0.96 [0.55–1.67]
Nausea vomiting	17.09	13.73	1.51 [0.73–3.29]	31.26	37.30	1.19 [0.70–2.05]
Anemia	0.00	0.00	NA	0.91	3.22	0.34 [0.02–3.64]
Hyperlipidemia	5.36	8.75	0.66 [0.21–1.98]	5.47	12.93	0.44 [0.14–1.27]
Traumatic injury	3.57	6.22	0.62 [0.15–2.35]	5.44	3.22	1.90 [0.43–13.09]

[§]Incidence rate was calculated as case per 1 000 person–years; MPH: methylphenidate; SSRI: selective serotonin reuptake inhibitor; ‡ statistically significant

© 2024 Lee DY et al. *JAMA Network Open*

eTable 5. Risk of outcome events between the fluoxetine and escitalopram groups in the subgroup analysis by sex

Outcomes	Male subgroup			Female subgroup		
	Incidence Rate[§]			Incidence Rate[§]		
	Fluoxetine (n = 1,000)	Escitalopram (Reference, n = 1,000)	HR [95% CI]	Fluoxetine (n = 1,502)	Escitalopram (Reference, n = 1,502)	HR [95% CI]
**Primary endpoint**						
Mania	2.50	4.74	0.52 [0.02–5.44]	3.72	3.54	1.01 [0.12–8.47]
Anxiety disorder	252.91	226.21	1.10 [0.76–1.58]	266.98	276.96	0.96 [0.71–1.29]
Sleep disorder	211.58	190.66	1.09 [0.77–1.55]	266.77	216.71	1.21 [0.91–1.62]
Tic disorder	17.96	14.58	1.23 [0.41–3.82]	22.60	28.90	0.78 [0.35–1.64]
Hospitalization	20.08	23.89	0.82 [0.31–2.08]	7.43	12.42	0.61 [0.16–2.03]
**Secondary endpoint**						
Tremor	5.02	4.77	1.03 [0.12–8.62]	16.78	5.32	3.27 [0.97–14.75]
Headache	2.51	7.14	0.36 [0.02–2.82]	9.34	5.34	1.68 [0.41–8.21]
Seizure	12.80	14.50	0.87 [0.25–2.87]	22.63	16.29	1.39 [0.59–3.40]
Dizziness	2.50	0.00	NA	3.76	8.94	0.42 [0.06–1.97]
Arrhythmia	10.15	11.97	0.83 [0.20–3.12]	16.83	14.32	1.17 [0.45–3.12]
Hypertension	2.50	11.94	0.21 [0.01–1.33]	7.51	23.44	**0.31 [0.09–0.88]**[‡]
Abdominal pain	7.55	4.73	1.61 [0.27–12.27]	7.42	5.30	1.34 [0.30–6.81]
Constipation	17.65	14.25	1.19 [0.40–3.71]	27.99	39.59	0.69 [0.35–1.32]
Nausea vomiting	27.75	38.36	0.71 [0.32–1.52]	37.85	39.25	0.92 [0.50–1.69]
Anemia	0.00	0.00	NA	1.86	8.88	0.20 [0.01–1.25]
Hyperlipidemia	0.00	11.90	NA	1.86	5.33	0.33 [0.02–2.62]
Traumatic injury	2.50	0.00	NA	5.57	3.54	1.53 [0.25–11.58]

[§]Incidence rate was calculated as case per 1 000 person–years; MPH: methylphenidate; SSRI: selective serotonin reuptake inhibitor; [‡] statistically significant

© 2024 Lee DY et al. *JAMA Network Open*

eTable 6. Comparisons of baseline characteristics, comorbidities, and concomitant drugs between the SSRI and MPH-only groups after propensity score matching in sensitivity analyses

Characteristics	As-treated 1:n			As-treated Stratification			ITT 1:1			ITT 1:n			ITT Stratification		
	SSRI (n=5,181), n (%)	MPH-only (n=7,341), n (%)	aSMD	SSRI (n=9,873), n (%)	MPH-only (n=7,361), n (%)	aSMD	SSRI (n=3,366), n (%)	MPH-only (n=3,366), n (%)	aSMD	SSRI (n=3,366), n (%)	MPH-only (n=4,568), n (%)	aSMD	SSRI (n=7,062), n (%)	MPH-only (n=4,573), n (%)	aSMD
**Socio-demographics**															
Male	2,534 (48.9)	3,582 (48.8)	0.00	4,818 (48.8)	3,526 (47.9)	0.02	1,716 (51.0)	1,733 (51.5)	0.01	1,716 (51.0)	2,330 (51.0)	0.00	3,531 (50.0)	2,218 (48.5)	0.03
18–39 years	4,461 (86.1)	6,306 (85.9)	0.01	8,501 (86.1)	6,286 (85.4)	0.01	2,868 (85.2)	2,848 (84.6)	0.01	2,868 (85.2)	3,882 (85.0)	0.01	5,960 (84.4)	3,841 (84.0)	0.01
40–64 years	637 (12.3)	925 (12.6)	0.01	1,135 (11.5)	869 (11.8)	0.01	441 (13.1)	454 (13.5)	0.01	441 (13.1)	617 (13.5)	0.01	982 (13.9)	631 (13.8)	0.01
≥ 65 years	83 (1.6)	110 (1.5)	0.01	237 (2.4)	206 (2.8)	0.02	57 (1.7)	64 (1.9)	0.01	57 (1.7)	69 (1.5)	0.01	120 (1.7)	101 (2.2)	0.02
Race, Korean	5,181 (100.0)	7,341 (100.0)	0.00	9,873 (100.0)	7,316 (100.0)	0.00	3,366 (100.0)	3,366 (100.0)	0.00	3,366 (100.0)	4,568 (100.0)	0.00	7,062 (100.0)	4,573 (100.0)	0.00
**Index year**															
2017	596 (11.5)	844 (11.5)	0.00	1,096 (11.1)	861 (11.7)	0.02	603 (17.9)	589 (17.5)	0.01	603 (17.9)	809 (17.7)	0.01	1,215 (17.2)	828 (18.1)	0.02
2018	1,016 (19.6)	1,402 (19.1)	0.02	1,856 (18.8)	1,384 (18.8)	0.00	983 (29.2)	993 (29.5)	0.01	983 (29.2)	1,352 (29.6)	0.01	2,090 (29.6)	1,367 (29.9)	0.01
2019	1,502 (29.0)	2,137 (29.1)	0.00	2,893 (29.3)	2,105 (28.6)	0.02	1,552 (46.1)	1,565 (46.5)	0.01	1,552 (46.1)	2,101 (46.0)	0.00	3,277 (46.4)	2,076 (45.4)	0.02
2020	2,067 (39.9)	2,958 (40.3)	0.01	4,028 (40.8)	3,011 (40.9)	0.00	228 (6.8)	219 (6.5)	0.01	228 (6.8)	306 (6.7)	0.00	480 (6.8)	302 (6.6)	0.01
**Medical history**															
Substance use disorder	114 (2.2)	154 (2.1)	0.01	247 (2.5)	184 (2.5)	0.00	71 (2.1)	77 (2.3)	0.02	71 (2.1)	105 (2.3)	0.02	191 (2.7)	128 (2.8)	0.00
Conduct disorder	52 (1.0)	73 (1.0)	0.00	99 (1.0)	73 (1.0)	0.00	34 (1.0)	34 (1.0)	0.00	34 (1.0)	59 (1.3)	0.01	92 (1.3)	55 (1.2)	0.01

© 2024 Lee DY et al. *JAMA Network Open*

Personality disorder	88 (1.7)	132 (1.8)	0.01	207 (2.1)	147 (2.0)	0.01	61 (1.8)	64 (1.9)	0.01	61 (1.8)	87 (1.9)	0.01	155 (2.2)	91 (2.0)	0.01
Autism spectrum disorder	26 (0.5)	37 (0.5)	0.01	59 (0.6)	41 (0.6)	0.00	20 (0.6)	24 (0.7)	0.00	20 (0.6)	27 (0.6)	0.00	42 (0.6)	27 (0.6)	0.00
Intellectual disability	52 (1.0)	74 (1.0)	0.01	79 (0.8)	66 (0.9)	0.00	40 (1.2)	44 (1.3)	0.01	40 (1.2)	59 (1.3)	0.01	71 (1.0)	55 (1.2)	0.02
**Medication use**															
Anticholinergics	41 (0.8)	73 (1.0)	0.02	79 (0.8)	66 (0.9)	0.02	27 (0.8)	34 (1.0)	0.02	27 (0.8)	46 (1.0)	0.02	63 (0.9)	50 (1.1)	0.01
Antiepileptics	497 (9.6)	712 (9.7)	0.00	1,175 (11.9)	890 (12.1)	0.00	296 (8.8)	303 (9.0)	0.01	296 (8.8)	416 (9.1)	0.01	876 (12.4)	572 (12.5)	0.00
Antipsychotics	855 (16.5)	1,240 (16.9)	0.01	1,925 (19.5)	1,524 (20.7)	0.03	515 (15.3)	512 (15.2)	0.00	515 (15.3)	699 (15.3)	0.00	1,370 (19.4)	937 (20.5)	0.03
Anxiolytics	1,191 (23.0)	1,776 (24.2)	0.03	3,307 (33.5)	2,620 (35.6)	0.05	710 (21.1)	730 (21.7)	0.01	710 (21.1)	1,005 (22.0)	0.02	2,436 (34.5)	1,633 (35.7)	0.03

ADHD: attention-deficit/hyperactivity disorder; PS: propensity score; MPH: methylphenidate; SSRI: selective serotonin reuptake inhibitor; aSMD: absolute standardized mean difference;

© 2024 Lee DY et al. *JAMA Network Open*

eTable 7. Comparisons of baseline characteristics, comorbidities, and concomitant drugs between the Fluoxetine and Escitalopram groups after propensity score matching in sensitivity analyses

Characteristics	As-treated 1:n			As-treated Stratification			ITT 1:1			ITT 1:n			ITT Stratification		
	Fluoxetine (n=2,577), n (%)	Escitalopram (n=5,105), n (%)	aSMD	Fluoxetine (n=2,791), n (%)	Escitalopram (n=5,150), n (%)	aSMD	Fluoxetine (n=1,915), n (%)	Escitalopram (n=1,915), n (%)	aSMD	Fluoxetine (n=1,915), n (%)	Escitalopram (n=3,552), n (%)	aSMD	Fluoxetine (n=2,118), n (%)	Escitalopram (n=3,592), n (%)	aSMD
**Socio-demographics**															
Male	1,023 (39.7)	1,940 (38.0)	0.04	1,256 (45.0)	2,266 (44.0)	0.02	795 (41.5)	777 (40.6)	0.02	795 (41.5)	1,399 (39.4)	0.04	985 (46.5)	1,609 (44.8)	0.03
18–39 years	2,237 (86.8)	4,446 (87.1)	0.00	2,437 (87.3)	4,434 (86.1)	0.01	1,651 (86.2)	1,672 (87.3)	0.01	1,651 (86.2)	3,065 (86.3)	0.01	1,819 (85.9)	3,032 (84.4)	0.01
40–64 years	319 (12.4)	628 (12.3)	0.00	332 (11.9)	639 (12.4)	0.01	255 (13.3)	230 (12.0)	0.02	255 (13.3)	465 (13.1)	0.01	286 (13.5)	478 (13.3)	0.01
≥ 65 years	21 (0.8)	31 (0.6)	0.02	22 (0.8)	77 (1.5)	0.05	9 (0.5)	13 (0.7)	0.04	9 (0.5)	22 (0.6)	0.02	13 (0.6)	82 (2.3)	0.06
Race, Korean	2,577 (100.0)	5,105 (100.0)	0.00	2,791 (100.0)	5,150 (100.0)	0.00	1,915 (100.0)	1,915 (100.0)	0.00	1,915 (100.0)	3,552 (100.0)	0.00	2,118 (100.0)	3,592 (100.0)	0.00
**Index year**															
2017	333 (12.9)	699 (13.7)	0.02	341 (12.2)	613 (11.9)	0.01	377 (19.7)	370 (19.3)	0.01	377 (19.7)	692 (19.5)	0.01	398 (18.8)	647 (18.0)	0.02
2018	528 (20.5)	1,047 (20.5)	0.00	539 (19.3)	1,009 (19.6)	0.01	582 (30.4)	584 (30.5)	0.00	582 (30.4)	1,073 (30.2)	0.01	635 (30.0)	1,085 (30.2)	0.01
2019	760 (29.5)	1,521 (29.8)	0.01	790 (28.3)	1,509 (29.3)	0.02	835 (43.6)	823 (43.0)	0.01	835 (43.6)	1,556 (43.8)	0.00	934 (44.1)	1,616 (45.0)	0.02
2020	956 (37.1)	1,838 (36.0)	0.02	1,121 (40.2)	2,019 (39.2)	0.02	121 (6.3)	138 (7.2)	0.03	121 (6.3)	231 (6.5)	0.01	151 (7.1)	244 (6.8)	0.01
**Medical history**															
Substance use disorder	72 (2.8)	133 (2.6)	0.01	75 (2.7)	129 (2.5)	0.01	59 (3.1)	55 (2.9)	0.01	59 (3.1)	107 (3.0)	0.01	70 (3.3)	108 (3.0)	0.02
Conduct disorder	23 (0.9)	41 (0.8)	0.00	25 (0.9)	41 (0.8)	0.01	21 (1.1)	19 (1.0)	0.01	21 (1.1)	39 (1.1)	0.00	23 (1.1)	36 (1.0)	0.01
Personality disorder	70 (2.7)	123 (2.4)	0.02	64 (2.3)	108 (2.1)	0.02	38 (2.0)	42 (2.2)	0.02	38 (2.0)	82 (2.3)	0.03	49 (2.3)	72 (2.0)	0.02
Autism spectrum disorder	18 (0.7)	36 (0.7)	0.01	17 (0.6)	31 (0.6)	0.00	17 (0.9)	15 (0.8)	0.00	17 (0.9)	28 (0.8)	0.01	15 (0.7)	29 (0.8)	0.01

© 2024 Lee DY et al. *JAMA Network Open*

Intellectual disability	21 (0.8)	41 (0.8)	0.00	22 (0.8)	41 (0.8)	0.00	21 (1.1)	20 (1.1)	0.01	21 (1.1)	39 (1.1)	0.00	21 (1.0)	32 (0.9)	0.00
**Medication use**															
Anticholinergics	18 (0.7)	41 (0.8)	0.01	25 (0.9)	41 (0.8)	0.01	13 (0.7)	19 (1.0)	0.03	13 (0.7)	36 (1.0)	0.03	19 (0.9)	32 (0.9)	0.01
Antiepileptics	260 (10.1)	521 (10.2)	0.00	338 (12.1)	597 (11.6)	0.02	211 (11.0)	213 (11.1)	0.00	211 (11.0)	394 (11.1)	0.00	263 (12.4)	445 (12.4)	0.00
Antipsychotics	526 (20.4)	1,031 (20.2)	0.01	583 (20.9)	1,061 (20.6)	0.01	396 (20.7)	402 (21.0)	0.01	396 (20.7)	742 (20.9)	0.01	460 (21.7)	776 (21.6)	0.00
Anxiolytics	959 (37.2)	1,828 (35.8)	0.03	1,186 (42.5)	2,086 (40.5)	0.04	722 (37.7)	686 (35.8)	0.04	722 (37.7)	1,296 (36.5)	0.03	911 (43.0)	1,487 (41.4)	0.03

ADHD: attention-deficit/hyperactivity disorder; PS: propensity score; MPH: methylphenidate; SSRI: selective serotonin reuptake inhibitor; aSMD: absolute standardized mean difference;

© 2024 Lee DY et al. *JAMA Network Open*

eTable 8. Results of sensitivity analyses between the SSRI and MPH–only groups

Analysis description	Mania	Anxiety disorder	Sleep disorder	Tic disorder	Hospitalization	Tremor	Headache	Seizure	Dizziness	Arrhythmia	Hypertension	Abdominal pain	Constipation	Nausea vomiting	Anemia	Hyperlipidemia	Traumatic injury	NC
**Main setting**	0.53 [0.19–1.40]	0.97 [0.83–1.13]	0.96 [0.82–1.13]	1.08 [0.67–1.75]	0.75 [0.47–1.21]	0.62 [0.33–1.14]	0.50 [0.24-0.99]‡	1.11 [0.72–1.75]	1.19 [0.53–2.84]	1.04 [0.60–1.85]	1.38 [0.80–2.47]	0.48 [0.16–1.35]	0.85 [0.57–1.27]	1.24 [0.82–1.90]	1.75 [0.37–12.30]	0.92 [0.45–1.93]	1.44 [0.55–4.16]	1.61 [0.34–11.27]
**AT PS matched (1: n)**	0.78 [0.28–2.04]	1.03 [0.89–1.19]	0.92 [0.80–1.06]	1.27 [0.81–2.00]	0.76 [0.49–1.18]	0.76 [0.42–1.37]	0.55 [0.28–1.03]	1.24 [0.83–1.88]	1.17 [0.57–2.44]	1.03 [0.63–1.67]	1.49 [0.90–2.50]	0.34 [0.12–0.81]‡	0.79 [0.55–1.14]	1.18 [0.81–1.72]	1.78 [0.43–8.75]	0.91 [0.48–1.73]	1.25 [0.54–2.98]	1.57 [0.38–7.74]
**AT PS stratified**	0.67 [0.30–1.61]	0.95 [0.82–1.09]	0.94 [0.82–1.08]	0.97 [0.64–1.50]	0.72 [0.47–1.10]	0.59 [0.34–1.06]	0.45 [0.24–0.83]‡	1.10 [0.74–1.65]	1.18 [0.58–2.48]	1.00 [0.61–1.68]	1.24 [0.75–2.10]	0.57 [0.27–1.22]	0.88 [0.63–1.24]	1.34 [0.94–1.93]	0.96 [0.26–4.86]	0.97 [0.51–1.89]	1.03 [0.43–2.60]	0.81 [0.21–3.80]
**ITT PS matched (1: 1)**	1.17 [0.39–3.62]	1.14 [0.98–1.33]	0.97 [0.84–1.24]	1.02 [0.66–1.61]	1.05 [0.69–1.60]	0.42 [0.22–0.77]‡	0.53 [0.27–1.04]	1.04 [0.69–1.58]	0.94 [0.47–1.87]	0.74 [0.39–1.38]	1.77 [0.99–3.27]	0.50 [0.13–1.59]	1.15 [0.76–1.75]	1.12 [0.70–1.78]	0.80 [0.20–3.02]	1.06 [0.54–2.07]	1.18 [0.53–2.69]	2.00 [0.53–9.48]
**ITT PS matched (1: n)**	1.06 [0.37–2.84]	1.13 [0.97–1.30]	0.93 [0.81–1.06]	1.21 [0.78–1.86]	0.86 [0.59–1.26]	0.51 [0.27–0.92]‡	0.56 [0.29–1.02]	1.03 [0.70–1.52]	0.91 [0.47–1.69]	0.72 [0.39–1.28]	1.77 [1.03–3.08]‡	0.39 [0.11–1.08]	0.89 [0.61–1.28]	0.89 [0.59–1.33]	0.68 [0.18–2.15]	1.11 [0.59–2.01]	0.58 [0.47–1.99]	1.36 [0.43–4.35]
**ITT PS stratified**	1.01 [0.41–2.66]	1.01 [0.89–1.16]	0.96 [0.85–1.09]	1.04 [0.71–1.57]	0.97 [0.69–1.37]	0.56 [0.35–0.90]‡	0.49 [0.29–0.84]‡	1.06 [0.75–1.52]	0.75 [0.42–1.37]	0.92 [0.56–1.55]	1.64 [0.97–2.83]	0.52 [0.22–1.23]	1.15 [0.81–1.55]	1.33 [0.79–1.64]	0.88 [0.37–2.32]	1.12 [0.62–2.06]	0.87 [0.42–1.82]	1.18 [0.39–3.89]
**30–days gap**	1.11 [0.41–3.11]	1.06 [0.89–1.26]	1.14 [0.96–1.34]	1.33 [0.82–2.18]	1.08 [0.66–1.76]	0.79 [0.41–1.50]	0.39 [0.17–0.84]‡	1.66 [1.04–2.71]‡	1.07 [0.49–2.41]	1.24 [0.68–2.31]	1.74 [0.91–3.49]	0.46 [0.14–1.34]	0.89 [0.57–1.38]	1.56 [1.00–2.46]	1.85 [0.39–12.97]	1.02 [0.52–2.04]	1.66 [0.59–5.35]	0.51 [0.07–3.16]
**All ADHD drugs**	0.85 [0.37–1.97]	0.90 [0.79–1.03]	0.99 [0.86–1.13]	1.42 [1.00–2.04]	0.90 [0.61–1.35]	0.61 [0.38–0.97]‡	0.69 [0.38–1.26]	0.82 [0.56–1.19]	1.18 [0.56–2.60]	0.92 [0.91–1.62]	1.64 [0.32–11.82]	0.72 [0.29–1.76]	1.19 [0.29–1.76]	1.06 [0.74–1.52]	1.19 [0.62–2.35]	0.91 [0.48–1.73]	1.43 [0.62–3.54]	2.11 [0.83–6.02]

© 2024 Lee DY et al. *JAMA Network Open*

eTable 9. Results of sensitivity analyses between the fluoxetine and the escitalopram groups

Analysis description	Mania	Anxiety disorder	Sleep disorder	Tic disorder	Hospitalization	Tremor	Headache	Seizure	Dizziness
Main setting	0.81 [0.20–3.07]	1.09 [0.87–1.37]	0.92 [0.50–1.60]	0.90 [0.50–1.60]	0.57 [0.25–1.21]	1.88 [0.72–5.47]	0.90 [0.32–2.51]	0.97 [0.53–1.77]	0.61 [0.13–2.51]
AT PS matched (1: n)	0.99 [0.26–3.14]	1.05 [0.86–1.27]	0.93 [0.77–1.12]	1.05 [0.62–1.75]	0.56 [0.26–1.08]	1.55 [0.69–3.40]	0.99 [0.37–2.37]	0.93 [0.54–1.54]	0.42 [0.10–1.30]
AT PS stratified	0.69 [0.18–2.30]	1.03 [0.84–1.25]	1.03 [0.85–1.24]	1.01 [0.60–1.69]	0.69 [0.35–1.28]	1.71 [0.75–3.88]	1.16 [0.46–2.75]	0.90 [0.52–1.51]	0.32 [0.07–1.01]
ITT PS matched (1: 1)	1.25 [0.33–5.05]	1.02 [0.83–1.26]	1.00 [0.83–1.22]	1.32 [0.76–2.32]	0.70 [0.39–1.21]	1.43 [0.55–3.93]	1.67 [0.62–4.90]	0.75 [0.42–1.31]	0.75 [0.25–2.15]
ITT PS matched (1: n)	0.84 [0.27–2.32]	0.98 [0.82–1.18]	0.90 [0.76–1.06]	1.29 [0.79–2.06]	0.68 [0.41–1.11]	0.81 [0.37–1.65]	1.32 [0.57–2.96]	0.68 [0.40–1.10]	0.56 [0.20–1.31]
ITT PS stratified	0.70 [0.21–2.04]	1.00 [0.84–1.21]	1.02 [0.86–1.20]	1.40 [0.85–2.27]	0.86 [0.53–1.36]	1.04 [0.48–2.17]	1.57 [0.70–3.54]	0.74 [0.45–1.21]	0.72 [0.29–1.67]
30–days gap	1.00 [0.24–4.23]	1.15 [0.94–1.43]	1.22 [0.99–1.50]	1.01 [0.57–1.79]	1.19 [0.55–2.61]	1.79 [0.77–4.49]	1.50 [0.54–4.48]	0.88 [0.49–1.57]	0.71 [0.21–2.23]
All ADHD drugs	0.84 [0.24–2.80]	1.16 [0.95–1.41]	0.99 [0.83–1.20]	0.98 [0.65–1.46]	0.80 [0.43–1.48]	1.26 [0.60–2.77]	2.24 [0.82–7.11]	0.79 [0.46–1.34]	0.51 [0.14–1.61]

Analysis description	Arrhythmia	Hypertension	Abdominal pain	Constipation	Nausea vomiting	Anemia	Hyperlipidemia	Traumatic injury	NC
Main setting	1.41 [0.65–3.14]	0.37 [0.10–1.09]	1.44 [0.46–4.85]	1.14 [0.65–2.02]	0.80 [0.49–1.29]	0.34 [0.02–2.63]	0.41 [0.06–1.91]	1.01 [0.19–5.47]	0.75 [0.10–4.53]
AT PS matched (1: n)	1.24 [0.64–2.34]	0.26 [0.08–0.67]‡	1.99 [0.68–5.83]	0.87 [0.54–1.35]	0.77 [0.50–1.15]	0.25 [0.01–1.34]	0.23 [0.04–0.81]‡	0.74 [0.16–2.55]	0.83 [0.12–3.84]
AT PS stratified	1.29 [0.66–2.45]	0.38 [0.15–0.82]‡	1.47 [0.53–4.32]	1.00 [0.63–1.56]	0.75 [0.49–1.15]	0.27 [0.01–1.59]	0.22 [0.03–0.82]‡	1.08 [0.27–3.74]	0.56 [0.08–2.75]
ITT PS matched (1: 1)	1.90 [0.90–4.26]	0.39 [0.15–0.89]‡	1.00 [0.24–4.23]	1.00 [0.61–1.64]	0.77 [0.46–1.27]	0.33 [0.05–1.45]	0.18 [0.03–0.68]‡	1.25 [0.33–5.06]	1.00 [0.19–5.40]
ITT PS matched (1: n)	2.08 [1.08–4.04]‡	0.37 [0.15–0.78]‡	1.23 [0.32–4.34]	0.79 [0.52–1.18]	0.72 [0.45–1.10]	0.34 [0.05–1.26]	0.15 [0.02–0.50]‡	1.55 [0.45–5.15]	1.11 [0.09–2.69]
ITT PS stratified	2.48 [1.24–5.01]‡	0.41 [0.17–0.88]‡	1.36 [0.38–4.65]	1.06 [0.71–1.58]	0.75 [0.47–1.15]	0.42 [0.09–1.49]	0.32 [0.09–0.88]‡	1.24 [0.34–4.10]	1.20 [0.24–4.99]
30–days gap	0.95 [0.48–1.89]	0.44 [0.17–1.03]	2.28 [0.74–8.42]	0.86 [0.49–1.50]	0.93 [0.58–1.47]	0.49 [0.02–5.19]	0.29 [0.04–1.20]	4.03 [0.60–78.72]	1.08 [0.13–9.01]
All ADHD drugs	1.39 [0.74–2.63]	0.45 [0.17–1.04]	1.18 [0.39–3.67]	1.12 [0.72–1.74]	0.95 [0.64–1.43]	0.68 [0.09–4.10]	NA	0.51 [0.11–1.92]	1.05 [0.19–5.67]

© 2024 Lee DY et al. *JAMA Network Open*

eTable 10. Comparisons of baseline characteristics, comorbidities, and concomitant drugs between the Antidepressants and MPH-only groups after propensity score matching in sensitivity analyses

Characteristics	AD (n=5,809), n (%)	MPH-only (n=5,809), n (%)	aSMD
**Socio-demographics**			
Male	2,901 (50.0)	2,906 (50.0)	0.00
18–39 years	4,990 (85.9)	4,996 (86.0)	0.00
40–64 years	732 (12.6)	720 (12.4)	0.00
≥ 65 years	87 (1.5)	93 (1.6)	0.00
Race, Korean	5,809 (100.0)	5,809 (100.0)	0.00
**Index year**			
2017	627 (10.8)	639 (11.0)	0.01
2018	1,110 (19.1)	1,063 (18.3)	0.02
2019	1,719 (29.6)	1,690 (29.1)	0.01
2020	2,353 (40.5)	2,417 (41.6)	0.02
**Psychiatric comorbidity**			
Substance use disorder	122 (2.1)	120 (2.1)	0.00
Conduct disorder	64 (1.1)	52 (0.9)	0.02
Personality disorder	99 (1.7)	97 (1.7)	0.00
Autism spectrum disorder	29 (0.5)	28 (0.5)	0.00
Intellectual disability	52 (0.9)	50 (0.9)	0.01
**Medication use**			
Anticholinergics	41 (0.7)	58 (1.0)	0.03
Antiepileptics	529 (9.1)	563 (9.7)	0.02
Antipsychotics	912 (15.7)	976 (16.8)	0.03
Anxiolytics	1,301 (22.4)	1,359 (23.4)	0.02

ADHD: attention-deficit/hyperactivity disorder; PS: propensity score; MPH: methylphenidate; AD: All types of antidepressants; aSMD: absolute standardized mean difference.

© 2024 Lee DY et al. *JAMA Network Open*

eTable 11. Results of sensitivity analyses between the Antidepressants and MPH–only groups

Outcomes	Incidence Rate[§]		HR [95% CI]
	AD (n=5 809)	MPH-only (n=5 809)	
**Primary endpoints**			
Mania	4.42	5.61	0.97 [0.41–2.37]
Anxiety disorder	239.18	286.39	0.97 [0.83–1.13]
Sleep disorder	218.88	237.50	1.07 [0.92–1.24]
Tic disorder	20.45	20.87	1.22 [0.80–1.90]
Hospitalization[†]	17.69	25.09	0.92 [0.61–1.40]
**Secondary endpoints**			
Tremor	7.52	15.09	**0.51 [0.28–0.92]**[‡]
Headache	6.17	13.85	**0.51 [0.27-0.95]**[‡]
Seizure	24.68	22.52	1.31 [0.87–1.99]
Dizziness	6.48	7.55	1.01 [0.49–2.17]
Arrhythmia	8.22	13.83	0.72 [0.40–1.30]
Hypertension	12.04	13.19	1.05 [0.61–1.84]
Abdominal pain	3.38	8.11	0.54 [0.23–1.23]
Constipation	20.50	35.76	0.83 [0.58–1.20]
Nausea vomiting	20.88	25.64	1.15 [0.77–1.72]
Anemia	1.02	1.24	0.87 [0.14–6.76]
Hyperlipidemia	7.51	10.05	0.78 [0.41–1.54]
Traumatic injury	4.74	4.36	1.20 [0.49–3.21]
**Negative control outcome**	1.02	1.24	0.93 [0.15–7.16]

MPH: methylphenidate; AD: All types of antidepressants;

[§]Incidence rate were calculated as case per 1 000 person-years; HR: hazard ratio; CI: 95% confidence interval;

[‡]statistically significant; [†]Hospitalization indicates a hospitalization with the presence of an ADHD diagnosis; Negative control outcome indicates respiratory tract infection.

© 2024 Lee DY et al. *JAMA Network Open*

eReferences

1. Kim C, Yu DH, Baek H, Cho J, You SC, Park RW. Data Resource Profile: Health Insurance Review and Assessment Service Covid-19 Observational Medical Outcomes Partnership (HIRA Covid-19 OMOP) database in South Korea. International Journal of Epidemiology. 2024;53(3):dyae062.

2. Kim J-W, Kim C, Kim K-H, et al. Scalable infrastructure supporting reproducible nationwide healthcare data analysis toward FAIR stewardship. Scientific Data. 2023;10(1):674.

3. Yoshida K, Solomon DH, Haneuse S, et al. A tool for empirical equipoise assessment in multigroup comparative effectiveness research. Pharmacoepidemiology and drug safety. 2019;28(7):934-941. doi:https://doi.org/10.1002/pds.4767
