# Supplementary material for: Combined Methylphenidate and Selective Serotonin Reuptake Inhibitors in Adults With Attention-Deficit/Hyperactivity Disorder
Source: JAMA Netw Open. 2024 Oct 9;7(10):e2438398. doi: 10.1001/jamanetworkopen.2024.38398 (PMC11581539; doi:10.1001/jamanetworkopen.2024.38398)
Supplement: Supplement 2. — Data Sharing Statement [file jamanetwopen-e2438398-s002.pdf]

Data Sharing Statement

Lee. Combined Methylphenidate and Selective Serotonin Reuptake Inhibitors in Adults With Attention-Deficit/Hyperactivity Disorder. *JAMA Netw Open*. Published October 09, 2024. doi:10.1001/jamanetworkopen.2024.38398

Data
Data available: No

Additional Information
Explanation for why data not available: Data are available from the corresponding authors upon reasonable request and with permission of HIRA
